# Selective C‐7 Functionalization of Phenanthridines by Microwave‐Assisted Claisen Rearrangements of 8‐Allyloxyphenanthridines

**DOI:** 10.1002/open.202300095

**Published:** 2023-07-21

**Authors:** Mathias Ryslett Lepsøe, Aleksander Granum Dalevold, Lise‐Lotte Gundersen

**Affiliations:** ^1^ Department of Chemistry University of Oslo P.O.Box 1033, Blindern 0315 Oslo Norway

**Keywords:** Claisen rearrangement, DFT calculations, microwave chemistry, nitrogen heterocycles, phenanthridines

## Abstract

Carbon‐carbon bond formation in the phenanthridine 7‐position was achieved by microwave‐assisted Claisen rearrangement of 8‐allyloxyphenanthridines. The reactions took place with excellent regioselectivity and high chemical yields. If the 7‐position was substituted, rearrangement to C‐9 took place, but the reaction occurred less readily. Rearrangements of 8‐allyloxy‐5,6‐dihydrophenanthridines (phenanthridines with a saturated B‐ring) gave a mixture of 7‐ and 9‐substituted products. The experimental results were supported by DFT (density functional theory) calculations.

## Graphical abstract

The phenanthridine skeleton is found in a wide variety of interesting compounds. Methods for CH functionalization and C−C bond formation in the phenanthridine C‐ring are almost non‐existing. We show that C−C bond formation in the 7‐position can be efficiently achieved through regioselective microwave‐assisted Claisen rearrangements of 8‐allyloxyphenanthridines.

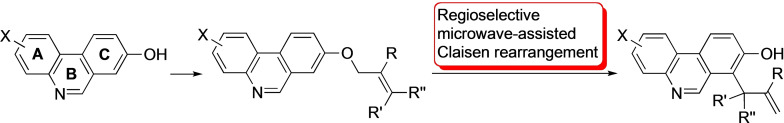

## Introduction

**Figure 1:**
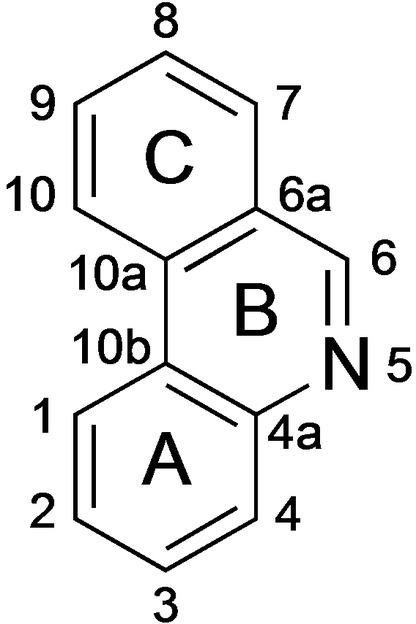
Numbering of the phenanthridine ring system.

The phenanthridine skeleton (Figure 1) is found in a wide variety of interesting compounds including natural products, (especially *Amaryllidaceae* alkaloids^[1]^) and synthetic molecules.^[2]^ Several phenanthridines are of medicinal interest, for instance as anticancer agents,^[3]^ anti‐infectives,^[4]^ anti‐diabetes‐^[5]^ and anti‐vitiligo compounds^[6]^ as well as PET tracers.^[7]^

There are many examples of late stage C−H functionalization and C−C bond formation reactions in the phenanthridine 6‐position, which resembles the 2‐position in quinolines,^[8]^ and to some extent also in the phenanthridine 4‐position (equivalent to C‐8 in quinoline).[^8^, ^9^] Examples of such reactions elsewhere in the phenanthridine A‐ring^[10]^ or in the C‐ring^[11]^ are, however, quite limited. The only reports on CH functionalization and C−C bond formation in the phenanthridine 7‐position, to date, are two examples of transition‐metal‐catalyzed phenylation of phenanthridin‐6(5*H*)‐one,[^11a^, ^11b^] and, in the 9‐position, some carbon substituents (*tert*‐butyl, cyclohexyl and 1‐adamantyl) have been introduced, generally in moderate yields, by a Minisci‐type reaction.^[11c]^ The latter reactions also required a rather bulky substituent at C‐6.

We have developed a synthetic route to phenanthridines with a microwave‐mediated IMDAF reaction of allylamino‐*ortho*‐furylarenes as a key‐step.^[12]^ Later, we showed that propargylamino‐*ortho*‐furylarenes cyclized to 8‐hydroxyphenanthridines.^[13]^ The hydroxyl group in the 8‐position offers possibilities for further functionalization in the C‐ring. We have previously demonstrated that the phenanthridinols can be transformed into the corresponding triflates and C‐substituents can be introduced at C‐8 by subsequent Suzuki cross‐couplings.^[13]^ An oxygen substituent also opens the possibility for late‐stage functionalization of the neighboring unsubstituted positions. We herein report on the regioselective Claisen rearrangement^[14]^ of 8‐allyloxyphenanthridines.

## Results and Discussion

**Scheme 1:**
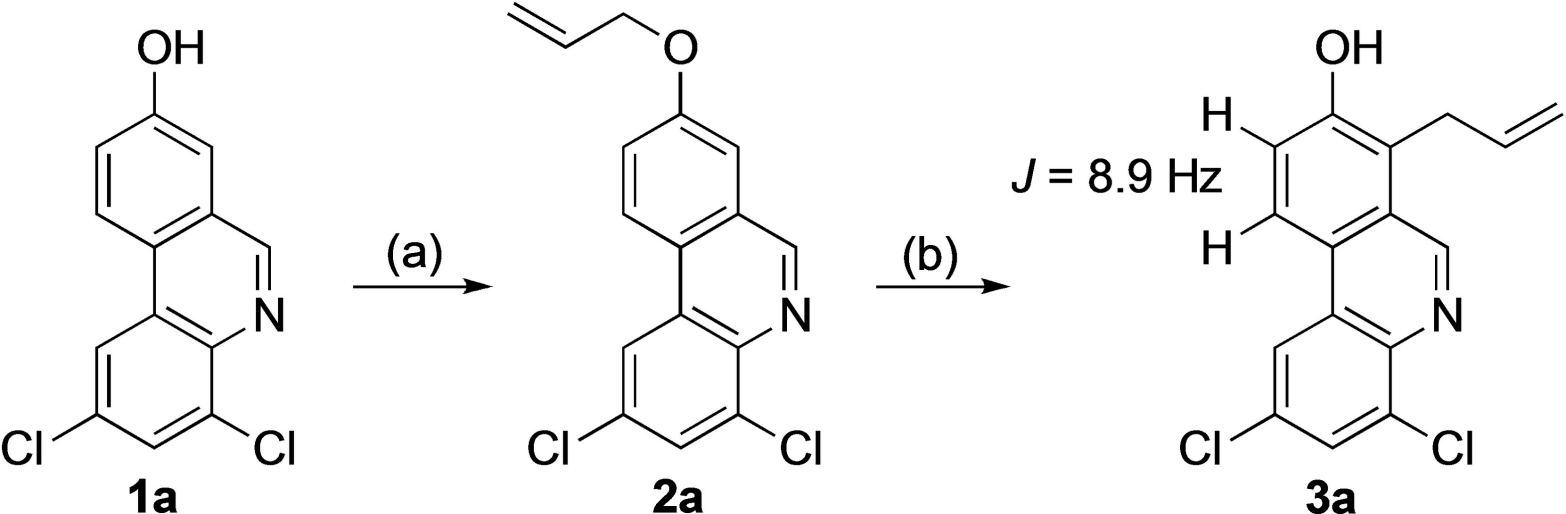
(a) BrCH_2_CH=CH_2_, K_2_CO_3_, DMF. (b) μW, see also Table 1.

Uncatalyzed Claisen rearrangements often require high reaction temperatures and extended reaction times. The use of microwave irradiation may shorten the reaction times substantially and thus eliminates problems with decomposition of starting material and/or product.^[14c]^ We first synthesized the allyloxyphenanthridine **2 a** from the phenanthridinol **1 a** ^[13]^ and subjected it to a microwave‐assisted Claisen rearrangement in *N,N*‐diethylaniline following a procedure for rearrangement of simpler allyloxyarenes^[15]^ (Scheme 1, Table 1, entries 1–3).

**Table 1:** Initial microwave‐assisted Claisen rearrangement of compound **2 a**.

Entry	Solvent	Temp. (°C)	Time (min)	Ratio **3 a** : **2 a** ^[a]^	Yield **3 a** (%)^[b]^
1	PhNEt_2_	200	60	45 : 55	18
2	PhNEt_2_	250	30	86 : 14	40
3	PhNEt_2_	250	60	>99 : 1	47
4	PhMe	250	30	83 : 17	70
5	PhMe	250	45	>99 : 1	95

[a] From the ^1^H NMR spectrum of the crude product. [b] Yield of isolated product.

Even though full conversion of the starting material was achieved (Table 1, entry 3), the yield of the isolated product was modest, most probably due to loss of the weakly basic compound **3 a** during extractions with acid in order to remove the basic and high‐boiling solvent. When the solvent was changed to toluene, the work‐up could be simplified to removal of the solvent and compound **3 a** was isolated in an excellent yield.

The Claisen rearrangement could, in theory, have proceeded to give the 7‐allylphenanthridine **3 a** or the 9‐substituted isomer, but the reaction turned out to be highly regioselective. The only allyl derivative identified in the crude product NMR spectrum was the 7‐substituted compound **3 a**. One characteristic feature in the ^1^H NMR spectrum of **3 a** was the relatively large coupling (*J*=8.9 Hz) between two aromatic protons; the vicinal hydrogens in the phenanthridine 9‐ and 10‐position (Scheme 1). The position of the allylic substituent in compound **3 a** was also supported by HMBC spectroscopy.

**Scheme 2:**
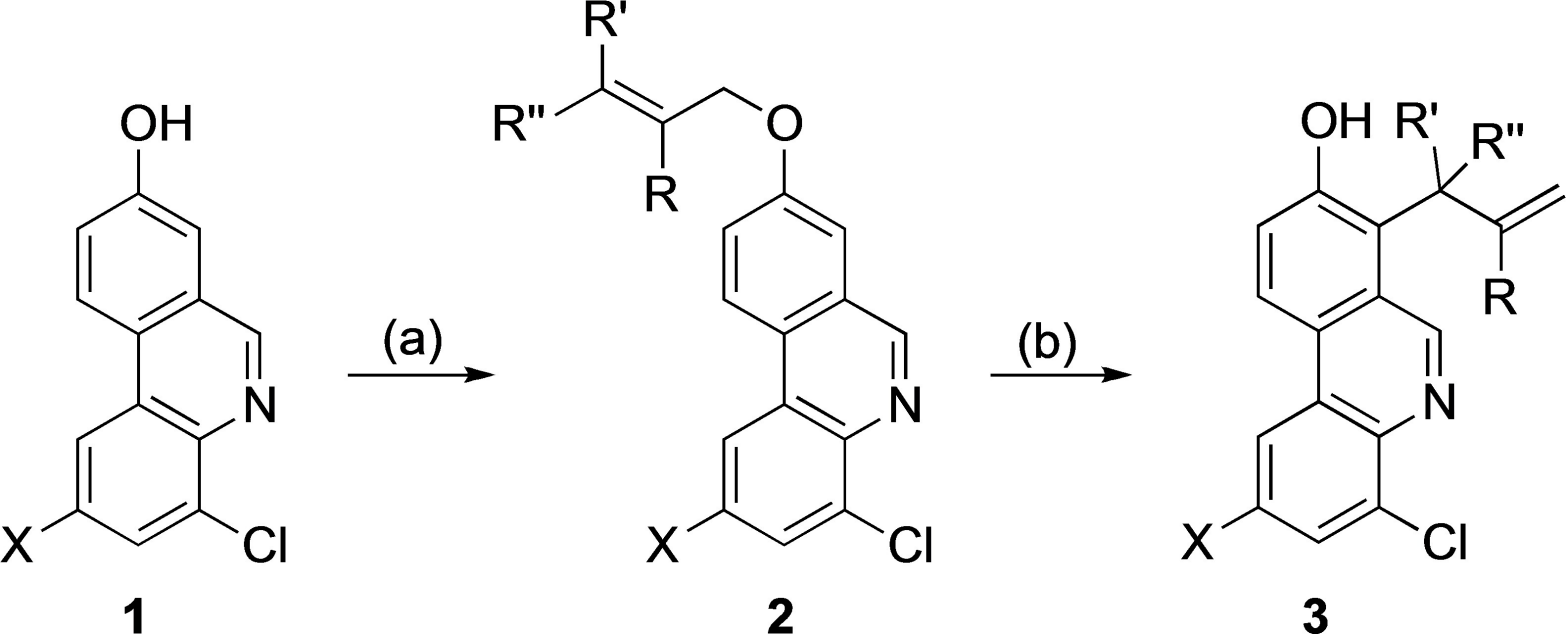
(a) Allylic bromide, K_2_CO_3_, DMF. (b) μW, see also Table 2.

Having found efficient conditions for regioselective rearrangement of the allyl ether **2 a**, we synthesized a series of 8‐allyloxyphenanthridines **2** and subjected them to the microwave‐assisted Claisen rearrangement (Scheme 2, Table 2).

**Table 2:** Microwave‐assisted Claisen rearrangements of compounds **2**.

Entry	Comp. **1**	R	R′	R′′	Yield **2** (%)^[a]^	Solv‐ent	Temp. (°C)	Time (min)	Yield **3** (%)^[a]^
1	X=Cl, **1 a**	H	H	H	98, **2 a**	PhMe	250	45	95, **3 a**
2	X=H, **1 b**	H	H	H	85, **2 b**	PhMe	250	45	86, **3 b**
3	X=NO_2_, **1 c**	H	H	H	78, **2 c**	PhMe	250	45	63, **3 c**
4	X=Cl, **1 a**	H	Me	H	86, **2 d** ^[b]^	PhMe	250	60	78, **3 d**
5	X=Cl, **1 a**	H	Me	Me	97, **2 e**	PhMe	250	45	–^[c]^
6						PhNEt_2_	250	45	–^[c]^
7						MeCN	220	60	–^[c]^
8						PhMe	220	25	–^[d]^
9						PhMe	180	30	–^[d]^
10						MeCN	180	20	–^[d]^
11						MeCN	180→200	40	–^[d]^
12						PhMe	210→220	25	–^[d]^
13	X=Cl, **1 a**	Me	H	H	96, **2 f**	PhMe	250	45	89, **3 f** ^[e]^
14	X=Cl, **1 a**	Cl	H	H	86, **2 g**	PhMe	250	45	83, **3 g**

**Figure 2:**
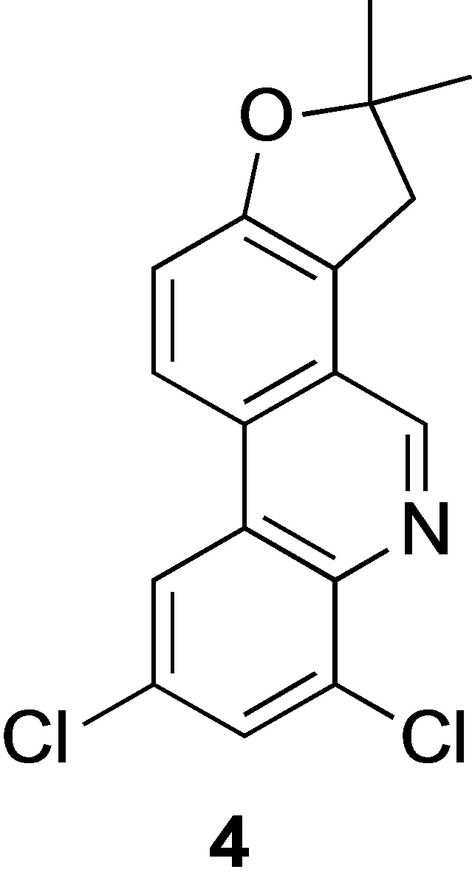
Structure of compound **4**.

[a] Yield of isolated product. [b] Formed as a 1 : 5 *Z/E* mixture. [c] Only compound **1 a** was observed (isolated in 85 % yield in entry 5). [d] No reaction. [e] Compound **4** (Figure 2) was also isolated (5 %).

The ethers **2** were formed in high yields and they generally selectively rearranged to give the corresponding 7‐allylphenanthridin‐8‐ols **3** (Table 2, entries 1–4 and 14). However, no rearrangement product was formed when the dimethylallyl ether **2 e** was subjected to the same reaction conditions. Here, extensive deallylation took place and the phenol **1 a** was isolated in 85 % yield (Table 2, entry 5). The choice of solvent (PhMe or MeCN, Table 2, entries 6 and 7) did not seem to have a significant impact on the outcome. When the reaction temperature was lowered and/or the reaction time shortened, no reaction took place at all (Table 2, entries 7–12). A similar phenomenon has been observed earlier during Claisen rearrangements of prenyloxycoumarins.^[16]^ Rearrangement products of such dimethylallyl ethers tended to quickly undergo a consecutive Cope rearrangement to the *para*‐position after the initial Claisen reaction. If the *para*‐position was blocked, like in our desired product **3 e**, elimination of the isoprene fragment from the Claisen product was observed. This was attributed to steric congestion.^[16]^

In the rearrangement of compound **2 f**, minor amounts (5 %) of the cyclized product **4** (Figure 2) were also isolated. The ^1^H NMR spectrum of the crude product showed that the cyclization took place during the reaction and not on the weakly acidic silica flash chromatography column. Cyclization of Claisen rearrangement products from β‐methylallyl aryl ethers has been observed before.^[17]^

**Scheme 3:**
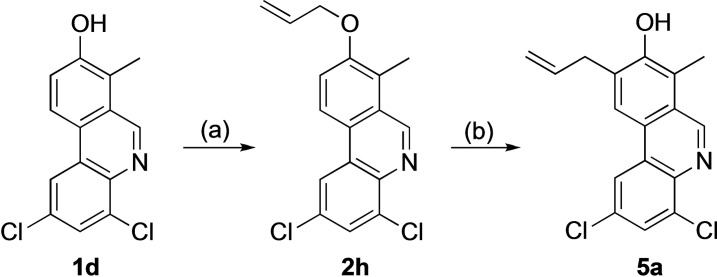
(a) BrCH_2_CH=CH_2_, K_2_CO_3_, DMF. (b) PhMe, μW, 250 °C, 240 min.

The Claisen rearrangements described above took place with excellent regioselectivity and only formation of 7‐allyl phenanthridinols **3** could be observed. Thus, we synthesized the 8‐allyloxyphenanthridine **2 h** where the 7‐position was occupied by a methyl substituent and subjected this compound to the same set of rearrangement conditions (Scheme 3). The reaction was substantially slower, and even after 240 min, 18 % unconverted starting material **2 h** were recovered. Furthermore, significant deallylation took place to give compound **1 d** (36 %) and the Claisen rearrangement product **5 a** was isolated in ca. 40 % yield together with minor amounts of an unidentified side product. However, it is worth remembering that other methods for late‐stage CH functionalization at C‐9 are very limited.^[11c]^

**Scheme 4:**
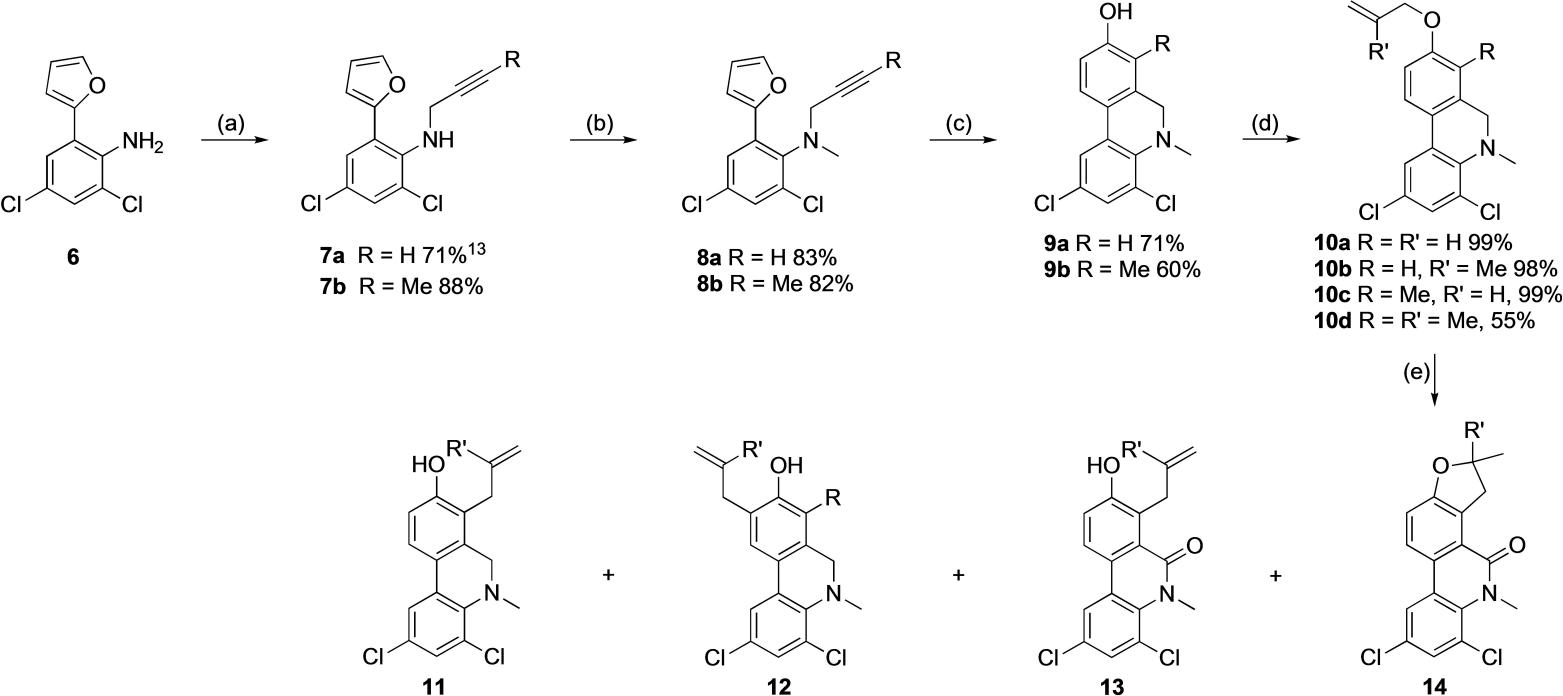
(a) NaH, TBAB, BrCH_2_C≡CR, THF, 45 °C. (b) NaH, MeI, DMF, 35 °C. (c) cat. HCl (aq.), MeCN, 180 °C, μW. (d) BrCHR′CH=CH_2_, K_2_CO_3_, DMF. (e) PhMe, μW, 250 °C, 45 min.

Finally, we wanted to examine the reactivity and regioselectivity of Claisen rearrangements of phenanthridines with a non‐aromatic B‐ring (for definition of B‐ring, see Figure 1). 5,6‐Dihydrophenanthridines with a free NH group are prone to oxidation to the corresponding fully aromatic phenanthridines.^[12b]^ Therefore, we synthesized the more stable *N*‐methyldihydrophenanthridines **10** from the known compound **6** ^[12b]^ (Scheme 4). When the allyloxydihydrophenanthridines **10 a** and **10 b** were heated in the microwave oven, the starting materials were consumed, but the Claisen rearrangements were rather unselective, leading to the formation of a substantial amount of both isomers **11** and **12**. (Scheme 4, Table 3, entries 1 and 2). Furthermore, oxidation in the B‐ring, leading to the phenanthridinones **13** and/or **14**, also took place. It is worth nothing that C‐6 oxidized side products were observed only from C‐7 substituted products **11**. As discussed below, an electron‐withdrawing “*meta*” substituent favors rearrangement to C‐7 over C‐9, and the C‐6 oxidation may have taken place before the rearrangement. In case of the starting material **10 b**, both phenanthridinones **13 b** and **14 b** were most probably observed, but, what was believed to be compound **13 b** was never isolated in pure form. Why apparently only the phenanthridinone **13 b**, but not the dihydrophenanthridines **11 b** or **12 b**, cyclized is somewhat difficult to explain, but may be attributed to a through‐space stabilization of the carbocation required for cyclization by the lone pair of electrons of the C‐6 carbonyl moiety.^[18]^

**Table 3:** Microwave‐assisted Claisen rearrangement of compounds **9**.

Entry	R	R′	Product distribution (**11** : **12** : **13** : **14**)^[a]^	Yield (%) **11** ^[b]^	Yield (%) **12** ^[b]^	Yield (%) **13** ^[b]^	Yield (%) **14** ^[b]^
1	H	H	53 : 41 : 6 : –^[c]^	44 (**11 a**)	33 (**12 a**)	ca. 5 (**13 a**)	–^[c]^
2	H	Me	47 : 34 : 5 : 14	30 (**11 b**)	24 (**12 b**)	–^[d]^	11 (**14 b**)
3	Me	H	Complex mixture	–	–	–	–
4	Me	Me	Complex mixture	–	–	–	–

[a] From the ^1^H NMR spectrum of the crude product. [b] Yield of isolated product. [c] Not observed or isolated. [d] Not isolated in pure form.

**Figure 3:**
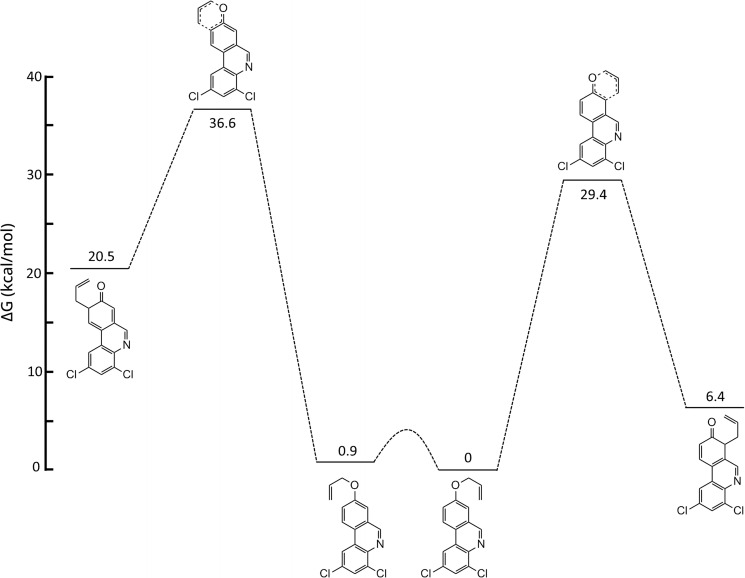
Reaction coordinate diagram showing relative Gibbs free energy in kcal/mol calculated using DFT at 298 K and 1 atm for the first step of the Claisen rearrangement of compound **2 a**.

**Figure 4:**
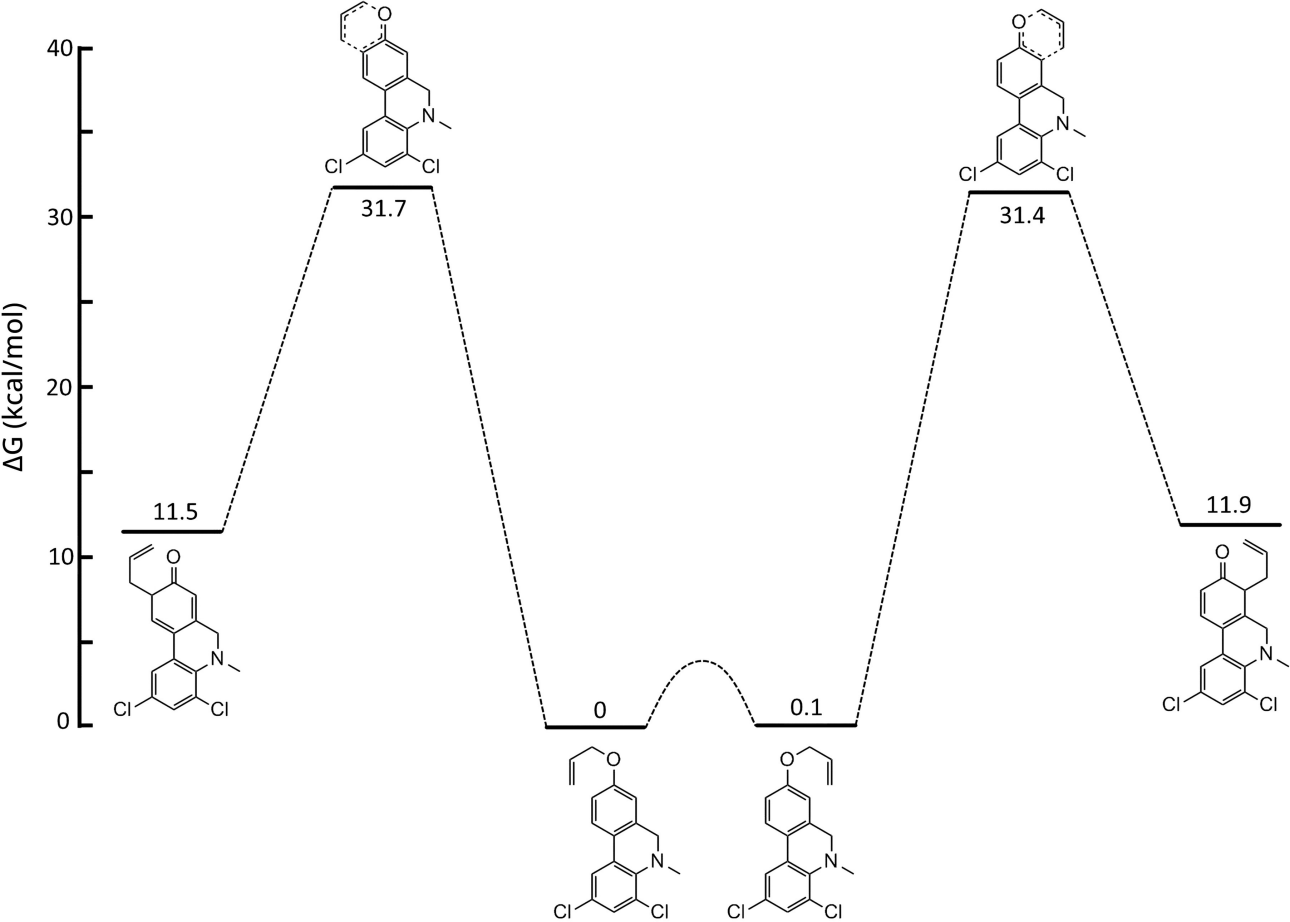
Reaction coordinate diagram showing relative Gibbs free energy in kcal/mol calculated using DFT at 298 K and 1 atm for the first step of the Claisen rearrangement of compound **10 a**.

It has been reported that the regiochemical outcome from Claisen rearrangements of *meta*‐substituted allyloxybenzene derivatives can be predicted from the preferential reactant ground state conformation or the less energetic transition state, or both.^[19]^ In order to shed more light on our experimental findings, DFT calculations at the B3LYP/def2‐TZVP level of starting materials, transition states and intermediates level were performed. These calculations showed that, for both the allyloxyphenanthridine **2 a** and the dihydro analog **10 a**, the conformations leading to the two possible rearrangement products were very close in energy (Figures 3 and 4). However, in case of the fully aromatic substrate **2 a** the transition state leading to the observed 7‐substituted phenanthridinol **3 a** was considerably lower in energy compared to the transition state leading to the not‐observed 9‐substituted isomer (Figure 3). On the other hand, the dihydro analog **10 a** rearranged with relatively low selectivity and here the energy difference between the two transition states was found to be small (Figure 4). The aromatic B‐ring in compounds **2** resembles an electron‐withdrawing (C=N) *meta* substituent relative to the allyloxy side chain. It has been reported earlier that electron‐withdrawing *meta* substituents on simple allyloxyarenes tend to favor Claisen rearrangements towards the *ortho* position, relative to the *meta* substituent, whereas electron‐donating *meta* substituents favor rearrangements in the opposite direction.^[20]^

## Conclusions

We have shown that readily available 8‐allyloxyphenanthridines undergo a Claisen rearrangement to give the corresponding 7‐allyl‐8‐hydroxy isomers with excellent regioselectivity. If C‐7 was substituted, rearrangement to C‐9 took place, but the reaction was considerably slower. Rearrangements of 8‐allyloxy‐5,6‐dihydrophenanthridines gave a mixture of 7‐ and 9‐substituted dihydrophenanthridin‐8‐ols. The experimental results were supported by DFT calculations and are in line with prior observations for Claisen rearrangements of simple allyloxyarenes. The chemistry described herein provides an efficient methodology for C−C bond formation of phenanthridines unsubstituted at the C‐7 position. As the only reported examples of C−H functionalization and C−C bond formation in the phenanthridine 7‐position are two examples of transition‐metal‐catalyzed phenylation of phenanthridin‐6(5*H*)‐one,[^11a^, ^11b^] our findings expand the synthetic toolbox for this interesting compound class.

## Experimental Section

**General procedure for the synthesis of 8‐allyloxyphenanthridines 2**. Compound **1** (0.19 mmol) and K_2_CO_3_ (53 mg, 0.38 mmol) in dry DMF (10 mL) was stirred under argon for 15 min, before the allylic bromide (0.38 mmol) was added. After stirring for additional 75 min, water (25 mL) was added and the resulting mixture was extracted with EtOAc (3×25 mL). The combined organic phases were dried (MgSO_4_), filtered through a plug of silica gel, eluting with hexanes (200 mL) and then EtOAc (250 mL) and the eluents was evaporated *in vacuo*.

**General procedure for the synthesis of 7‐allylphenanthridin‐8‐ols 3**. A 0.2–0.3 m solution of compound **2** in toluene under argon was stirred at 250 °C for 45 min in the microwave reactor. The mixture was evaporated *in vacuo* and the product was purified by flash chromatography on silica gel eluting with CH_2_Cl_2_−EtOAc‐hexanes (1 : 3 : 36).

## Supporting Information Summary

Additional references are cited within the Supporting Information.[^21^, ^22^, ^23^, ^24^, ^25^, ^26^, ^27^] The Supporting Information contains description of DFT calculations and procedures and spectral data for all novel compounds as well as copies of ^1^H and ^13^C NMR spectra of all novel compounds.

## Conflict of interest

The authors declare no conflict of interest.

1

## Supporting information

As a service to our authors and readers, this journal provides supporting information supplied by the authors. Such materials are peer reviewed and may be re‐organized for online delivery, but are not copy‐edited or typeset. Technical support issues arising from supporting information (other than missing files) should be addressed to the authors.

Supporting Information

## Data Availability

The data that support the findings of this study are available in the supplementary material of this article.
