# Supplementary material for: Selective C‐7 Functionalization of Phenanthridines by Microwave‐Assisted Claisen Rearrangements of 8‐Allyloxyphenanthridines
Source: ChemistryOpen. 2023 Jul 21;12(7):e202300095. doi: 10.1002/open.202300095 (PMC10362114; doi:10.1002/open.202300095)

ChemistryOpen

Supporting Information

Selective C-7 Functionalization of Phenanthridines by Microwave-Assisted Claisen Rearrangements of 8-Allyloxyphenanthridines

Mathias Ryslett Lepsøe, Aleksander Granum Dalevold, and Lise-Lotte Gundersen*

Table of content

General Information	2
DFT Calculations	2
Synthesis and Characterization of Novel Compounds	3
References	12
^{1}H NMR and ^{13}C NMR Spectra of Novel Compounds	13

General Information

^{1}H NMR spectra were recorded at 600 MHz on a Bruker AV 600 or Bruker AVII 600 instrument, at 400 MHz on a Bruker AVII 400 instrument or at 300 MHz on a Bruker DPX 300 instrument. The decoupled ^{13}C NMR spectra were recorded at 150 or 100 MHz using instruments mentioned above. High resolution mass spectra under under electron-spray (ESI) condition were recorded on a Bruker Maxis II ETD or a Micromass Q-Tof-2 instrument. Melting points were determined on a Büchi Melting Point B-545 apparatus and are uncorrected. Microwave experiments were carried out in sealed vessels in a synthesis reactor (Monowave 300, Anton Paar GmbH) equipped with an internal IR probe calibrated with a Ruby thermometer. HPLC grade acetonitrile was degassed by freeze-pump-thaw cycling using N_2(l) and flushed with Ar before use in microwave reactions. Dry DMF and THF were obtained from the solvent purification system MB SPS-800 from MBraun. Toluene and *N,N*-diethylaniline were dried over 4Å molecular sieves (4Å MS) for at least one day, followed by distillation under vacuum from fresh 4Å MS. All other reagents were commercially available and used as received. Starting materials available by literature methods: 2-Chloro-6-(furan-2-yl)aniline[12a] 2,4-dichlorophenanthridin-8-ol (**1a**),[13] 4-chloro-2-nitrophenanthridin-8-ol (**1c**)[13] 2,4-dichloro-7-methylphenanthridin-8-ol (**1d**).[12c] 2,4-dichloro-6-(furan-2-yl)aniline (**6**),[12b] 2,4-dichloro-6-(furan-2-yl)-*N*-(prop-2-yn-1-yl)aniline (**7a**).[13]

DFT Calculations

Geometry optimizations were performed using density functional theory (DFT) in ORCA 4.2.116[21] at the B3LYP[22] level of theory with D4 dispersion corrections.[23] The def2-TZV[24] basis set with default grid settings was used for all calculations. The calculations were performed on an AMD Ryzen 7 1700 processor utilizing 6 cores at 3.7 GHz, far from the processing capabilities commonly used for these applications. To speed up calculations, resolution of identity (RI) approximation together with the def2/J auxiliary basis set[25] was used for the calculation of the Coulomb integrals and the chain-of-spheres exchange (COSX)[26] approximation for the Hartree-Fock exchange. These approximations leads to approximately 25 times faster calculations of the Couloumb integrals and up to two orders of magnitude speed up of the exchange at the cost of very small errors in the optimized geometry and the final energy. Harmonic vibrational frequency calculations were performed on the optimized geometries to confirm that a local minimum was reached. A few optimized geometries, when using default optimization thresholds, had imaginary frequencies in the range of 1-20 wave numbers, which was not removed by perturbing the geometry along the offending mode and doing a reoptimization. While strictly not necessary for such low wave numbers, using very tight optimization thresholds solved the problem completely. The transition states were found by using the nudged elastic band (NEB-TS)[27] method implemented in ORCA, using the optimized allyl aryl ether and intermediate as input structures. Transition state geometries were confirmed to have a single imaginary vibrational frequency.

Synthesis and Characterization of Novel Compounds

4-Chlorophenanthridin-8-ol (1b). Sodium hydride (ca. 60 % in mineral oil, 352 mg, ca. 8.80 mmol) was added to a stirring solution of tetrabutylammonium bromide (2.12 g, 6.58 mmol) and 2-chloro-6-(furan-2-yl)aniline[13] (602 mg, 3.11 mmol) in dry THF (50 mL) at ambient temperature under Ar. The mixture was stirred for 15 min at 45 °C before dropwise addition of propargyl bromide (ca. 80 % in toluene, 0.38 mL, ca. 4.0 mmol). After 22 h, the reaction was quenched with water (25 mL), extracted with CH_2Cl_2 (5×25 mL), dried ($MgSO_4$) and evaporated *in vacuo*. The product was purified by flash chromatography on SiO_2 eluting with CH_2Cl_2-hexane (1:3) followed by acetone-hexane (1:49); yield 528 mg (73%) 2-chloro-6-(furan-2-yl)-*N*-(prop-2-ynyl)aniline, colorless oil. ^{1}H NMR (600 MHz, $CDCl_3$) δ 7.49 (dd, *J* = 7.8, 1.5 Hz, 1H, H-5), 7.48 (dd, *J* = 1.8, 0.8 Hz, 1H, H-5 in furyl), 7.28 (dd, *J* = 7.8, 1.5 Hz, 1H, H-3), 6.96 (t, *J* = 7.8 Hz, 1H, H-5), 6.84 (dd, *J* = 3.4, 0.8 Hz, 1H, H-3 in furyl), 6.49 (dd, *J* = 3.4, 1.8 Hz, 1H, H-4 in furyl), 4.39 (br s, 1H, NH), 3.73 (d, *J* = 2.6 Hz, 2H, NCH_2), 2.15 (t, *J* = 2.6 Hz, 1H, ≡CH); ^{13}C NMR (150 MHz, $CDCl_3$) δ 151.4 (C-2 in furyl), 142.2 (C-5 in furyl), 141.1 (C-1), 129.1 (C-3), 128.1 (C-2 or C-6), 127.5 (C-5), 124.9 (C-2 or C-6), 123.4 (C-4), 11.9 (C-4 in furyl), 109.3 (C-3 in furyl), 81.3 (C≡), 72.1 (≡CH), 36.8 (NCH_2); HRMS (ESI) calcd. for $C_{13}H_9ClNO$ $[M{+}H]^+$ 232.0524, found 232.0524. A stirring solution of 2-chloro-6-(furan-2-yl)-*N*-(prop-2-ynyl)aniline (74 mg, 0.32 mmol) and aq. HCl (0.2 M, 2 drops) in degassed CH_3CN (7 mL) was flushed with Ar for 15 min in a reactor tube and heated in the microwave oven at 180 °C for 150 min. The cooled mixture was transferred to a quartz tube and the microwave tube washed with CH_3CN (15 mL) subsequently transferred to the quartz tube. Air was bubbled through the mixture while exposed to UV light (315–400 nm) at ambient temperature for 1 h. The solvent was removed *in vacuo* and the product purified by flash chromatography on SiO_2 eluting with EtOAc-hexane (2:3); yield 52 mg (72%), off-white solid, mp 219-220 °C. ^{1}H-NMR (400 MHz, DMSO-d_6) δ 10.41 (s, 1H, OH), 9.32 (s, 1H, H-6), 8.71 (d, *J* = 9.6 Hz, 1H, H-10), 8.65 (d, *J* = 7.9 Hz, 1H, H-3), 7.83 (d, *J* = 7.9 Hz, 1H, H-1), 7.64 (apparent t, *J* = 7.9 Hz, 1H, H-2), 7.49 (m, 2H, H-9 and H-7); ^{13}C-NMR (100 MHz, DMSO-d_6) δ 157.6 (C-10a), 153.6 (C-6), 138.8 (C-4), 133.2 (C-10b), 127.9 (C-6a), 127.6 (C-4a), 127.4 (C-1), 126.0 (C-2), 124.6 (C-8), 124.5 (C-9), 122.7 (C-7), 121.5 (C-3), 111.1 (C-10); HRMS (ESI) calcd. for $C_{13}H_8ClNO$ $[M{+}Na]^+$ 252.0187, found 252.0187.

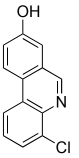

General procedure for the synthesis of 8-allyloxyphenanthridines 2.

Compound **1** (0.19 mmol) and K_2CO_3 (53 mg, 0.38 mmol) in dry DMF (10 mL) was stirred under Ar for 15 min, before the allylic bromide (0.38 mmol) was added. After stirring for additional 75 min, water (25 mL) was added and the resulting mixture was extracted with EtOAc (3×25 mL). The combined organic phases were dried ($MgSO_4$), filtered through a plug of SiO_2, eluting with hexanes (200 mL) followed by EtOAc (250 mL) and the eluents was evaporated *in vacuo.*

8-(Allyloxy)-2,4-dichlorophenanthridine (2a). The reaction was run in 1.14 mmol scale. Yield 340 mg (98%), colorless solid, mp 152-153 °C. ^{1}H NMR (600 MHz, acetone-d_6) δ 9.37 (s, 1H, H-6), 8.80 (d, *J* = 8.7 Hz, 1H, H-10), 8.71 (d, *J* = 2.2 Hz, 1H, H-1), 7.88 (d, *J* = 2.2 Hz, 1H, H-3), 7.77 (d, *J* = 2.6 Hz, 1H, H-7), 7.66 (dd, *J* = 8.7, 2.6 Hz, 1H, H-9), 6.23-6.17 (m, 1H, CH=), 5.56-5.53 (m, 1H, H_A in =CH_2), 5.37-5.35 (m, 1H, H_B in =CH_2), 4.86-4.87 (m, 2H, OCH_2); ^{13}C NMR (150 MHz, acetone-d_6) δ 159.1 (C-8), 153.7 (C-6), 138.6 (C-4a), 135.4 (C-4), 133.2 (=CH), 132.0 (C-2), 128.5 (C-6a), 127.8 (C-3), 126.8 (C-10b), 125.4 (C-10a), 124.6 (C-10), 123.0 (C-9), 121.0 (C-1), 117.3 (=CH_2), 109.5 (C-7), 69.0 (OCH_2); HRMS

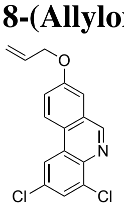

(ESI) calcd. for $C_{16}H_{11}Cl_2NO$ $[M]^+$ 303.0218, found 303.0212.

8-(Allyloxy)-4-chlorophenanthridine (2b). The reaction was run in 0.260 mmol scale. Yield 63 mg (85%), colorless solid, mp 99-100 °C. ^{1}H NMR (600 MHz, $CDCl_3$) δ 9.31 (s, 1H, H-6), 8.50 (d, J = 9.0 Hz, 1H, H-10), 8.41 (dd, J = 8.3, 0.8 Hz, 1H, H-1), 7.78 (dd, J = 7.6, 1.2 Hz, 1H, H-3), 7.55 (t, J = 8.0 Hz, 1H, H-2), 7.53 (dd, J = 9.0, 2.6 Hz, 1H, H-9), 7.41 (d, J = 2.6 Hz, 1H, H-7), 6.16-6.09 (m, 1H, CH=), 5.51-5.48 (m, 1H, H_A in $=CH_2$), 5.37-5.35 (m, 1H, H_B in $=CH_2$), 4.73-4.72 (m, 2H, OCH_2); ^{13}C NMR (150 MHz, $CDCl_3$) δ 158.6 (C-8), 153.5 (C-6), 140.0 (C-4a), 134.4 (C-4), 132.7 (=CH), 128.4 (C-3), 128.0 (C-10a), 127.4 (C-2), 127.1 (C-6a), 126.3 (C-10b), 124.2 (C-10), 123.3 (C-9), 121.0 (C-1), 118.6 ($=CH_2$), 109.4 (C-7), 69.4 (OCH_2); HRMS (ESI) calcd. for $C_{16}H_{12}ClNO$ $[M]^+$ 269.0607, found 269.0602.

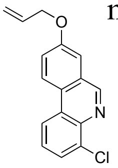

8-(Allyloxy)-4-chloro-2-nitrophenanthridine (2c). The reaction was run in 0.370 mmol scale. Yield 91 mg (78%), colorless solid, mp 180-181 °C. ^{1}H NMR (400 MHz, $CDCl_3$) δ 9.42 (s, 1H, H-6), 9.30 (d, J = 2.4 Hz, 1H, H-1), 8.58 (d, J = 9.3 Hz, 1H, H-10), 8.57 (d, J = 2.4 Hz, 1H, H-3), 7.64 (dd, J = 9.0, 2.6 Hz, 1H, H-9), 7.47 (d, J = 2.6 Hz, 1H, H-7), 6.18-6.08 (m, 1H, =CH), 5.53-5.48 (m, 1H, H_A in $=CH_2$), 5.40-5.37 (m, 1H, H_B in $=CH_2$), 4.77-4.75 (m, 2H, OCH_2); ^{13}C NMR (100 MHz, $CDCl_3$) δ 159.7 (C-8), 156.8 (C-6), 145.5 (C-2), 143.0 (C-4a), 136.2 (C-4), 132.3 (=CH), 128.5 (C-6a), 127.0 (C-10a), 125.7 (C-10b), 124.5 (C-9 or C-10), 124.4 (C-9 or C-10), 122.1 (C-3), 118.9 ($=CH_2$), 117.2 (C-1), 109.9 (C-7), 69.6 (OCH_2); HRMS (ESI) calcd. for $C_{16}H_{11}ClN_2O_3$ $[M]^+$ 314.0458, found 314.0452.

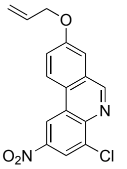

8-(But-2-en-1-yloxy)-2,4-dichlorophenanthridine (2d). The reaction was run in 0.19 mmol scale. Yield 52 mg (86%) as a 1:5 Z/E-mixture, colorless solid, mp 140-141 °C. Only NMR data for the major E-isomer: ^{1}H NMR (600 MHz, acetone-d_6) δ 9.35 (s, 1H, H-6), 8.77 (d, J = 9.0 Hz, 1H, H-10), 8.69 (d, J = 2.2 Hz, 1H, H-1), 7.85 (d, J = 2.2 Hz, 1H, H-3), 7.74 (d, J = 2.7 Hz, 1H, H-7), 7.61 (dd, J = 9.0, 2.7 Hz, 1H, H-9), 6.03-5.97 (m, 1H, $CH_2CH=$), 5.86-5.79 (m, 1H, $CH_3CH=$), 4.75 (d, J = 6.1 Hz, 2H, OCH_2), 1.77 (dd, J = 6.5, 1.2 Hz, 3H, CH_3); ^{13}C NMR (150 MHz, acetone-d_6) δ 160.3 (C-8), 154.7 (C-6), 139.5 (C-4a), 136.4 (C-4), 133.0 (C-2), 131.4 ($CH_2CH=$), 129.5 (C-6a), 128.7 (C-3), 127.8 (C-10b), 126.9 ($CH_3CH=$), 126.3 (C-10a), 125.5 (C-10), 124.1 (C-9), 121.9 (C-1), 110.4 (C-7), 69.8 (OCH_2), 18.0 (CH_3). Partial NMR data for the minor Z-isomer: ^{1}H NMR (600 MHz, acetone-d_6) δ 7.77 (d, J = 2.7 Hz, 1H, H-7), 4.98 (d, J 5.0 Hz, 2H, OCH_2), 1.81 (m, 3H CH_3), other phenanthridine signals were overlapping with phenanthridine signals from the E-isomer and both CH= signals were overlapping with $CH_3CH=$ from the E-isomer. HRMS (ESI) calcd. for $C_{17}H_{13}Cl_2NO$ $[M]^+$ 317.0374, found 317.0364.

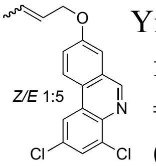

2,4-Dichloro-8-[(3-methylbut-2-en-1-yl)oxy]phenanthridine (2e). The reaction was run in 0.19 mmol scale. Yield 61 mg (97%), colorless solid, mp 165-166 °C. ^{1}H NMR (600 MHz, $CDCl_3$) δ 9.29 (s, 1H, H-6), 8.39 (d, J = 9.0 Hz, 1H, H-10), 8.35 (d, J = 2.1 Hz, 1H, H-1), 7.75 (d, J = 2.1 Hz, 1H, H-3), 7.52 (dd, J = 9.0, 2.5 Hz, 1H, H-9), 7.41 (d, J = 2.5 Hz, 1H, H-7), 5.57-5.54 (m, 1H, =CH), 4.70 (d, J = 6.8 Hz, 2H, OCH_2), 1.83 (s, 3H, CH_3), 1.80 (s, 3H, CH_3); ^{13}C NMR (150 MHz, $CDCl_3$) δ 159.4 (C-8), 153.6 (C-6), 139.6 (=C), 138.3 (C-4a), 135.2 (C-4), 132.9 (C-2), 128.6 (C-3), 128.3 (C-6a), 126.9 (C-10b), 125.9 (C-10a), 124.2 (C-10), 123.9 (C-9), 120.6 (C-1), 118.9 (=CH), 109.3 (C-7), 65.6 (OCH_2), 26.1 (CH_3), 18.6 (CH_3); HRMS (ESI) calcd. for $C_{18}H_{15}Cl_2NO$ $[M]^+$ 331.0531, found 331.0524.

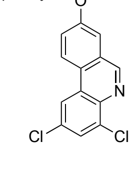

2,4-Dichloro-8-[(2-methylallyl)oxy]phenanthridine (2f). The reaction was run in 0.19 mmol scale. Yield 58 mg (96%), colorless solid, mp 154-155 °C. ^{1}H NMR (600 MHz, $CDCl_3$) δ 9.27 (s, 1H, H-6), 8.41 (d, J = 9.1 Hz, 1H, H-10), 8.36 (d, J = 2.1 Hz, 1H, H-1), 7.75 (d, J = 2.1 Hz, 1H, H-3), 7.54 (dd, J = 9.1, 2.6 Hz, 1H, H-9), 7.41 (d, J = 2.6 Hz, 1H, H-7), 5.17 (s, 1H, H_A in $=CH_2$), 5.06 (s, 1H, H_B in $=CH_2$), 4.62 (s, 2H, OCH_2), 1.89 (s, 3H, CH_3); ^{13}C NMR (150 MHz, $CDCl_3$) δ 159.2 (C-8), 153.7 (C-6), 140.2 (=C), 138.7 (C-4a), 135.5 (C-4), 132.8 (C-2), 128.5 (C-3), 128.3 (C-6a), 126.8 (C-10b), 126.0 (C-10a), 124.2 (C-10), 123.5 (C-9), 120.6 (C-1), 113.7 ($=CH_2$), 109.6 (C-7), 72.4 (OCH_2), 19.7 (CH_3); HRMS (ESI) calcd. for $C_{17}H_{13}Cl_2NO$ $[M]^+$ 317.0374, found 317.0368.

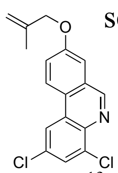

2,4-Dichloro-8-[(2-chloroallyl)oxy]phenanthridine (2g). The reaction was run in 0.19 mmol scale. Yield 55 mg (86%), colorless solid, mp 169-170 °C. ^{1}H NMR (600 MHz, $CDCl_3$) δ 9.27 (s, 1H, H-6), 8.44 (d, J = 9.1 Hz, 1H, H-10), 8.36 (d, J = 2.2 Hz, 1H, H-1), 7.77 (d, J = 2.2 Hz, 1H, H-3), 7.56 (dd, J = 9.1, 2.6 Hz, 1H, H-9), 7.41 (d, J = 2.6 Hz, 1H, H-7), 5.63 (d, J = 1.7 Hz, 1H, H_A in $=CH_2$), 5.51 (d, J = 1.7 Hz, 1H, H_B in $=CH_2$), 4.77 (s, 2H, OCH_2); ^{13}C NMR (150 MHz, $CDCl_3$) δ 158.2 (C-8), 153.5 (C-6), 138.9 (C-4a), 135.7 (C-4), 135.6 (=C), 132.9 (C-2), 128.8 (C-3), 128.2 (C-6a), 126.7 (C-10b), 126.5 (C-10a), 124.5 (C-10), 123.1 (C-9), 120.7 (C-1), 114.7 ($=CH_2$), 109.9 (C-7), 70.6 (OCH_2); HRMS (ESI) calcd. for $C_{16}H_{10}Cl_3NO$ $[M]^+$ 336.9828, found 336.9822.

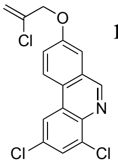

8-(Allyloxy)-2,4-dichloro-7-methylphenanthridine (2h). The reaction was run in 0.37 mmol scale. Yield 53 mg (93%), colorless solid, mp 167-168 °C. ^{1}H NMR (600 MHz, $CDCl_3$) δ 9.62 (s, 1H, H-6), 8.34 (d, J = 2.1 Hz, 1H, H-1), 8.32 (d, J = 9.1 Hz, 1H, H-10), 7.73 (d, J = 2.1 Hz, 1H, H-3), 7.49 (d, J = 9.1 Hz, 1H, H-9), 6.14-6.08 (m, 1H, =CH), 5.49-5.46 (m, 1H, H_A in $=CH_2$), 5.34-5.33 (m, 1H, H_B in $=CH_2$), 4.73-4.72 (m, 2H, OCH_2), 2.71 (s, 3H, CH_3); ^{13}C NMR (150 MHz, $CDCl_3$) δ 156.2 (C-8), 151.2 (C-6), 138.1 (C-4a), 135.4 (C-4), 133.1 (=CH), 132.6 (C-2), 128.4 (C-3), 126.9 (C-6a), 126.6 (C-10b), 125.7 (C-10a), 123.7 (C-7), 121.4 (C-10), 120.7 (C-1), 118.3 (C-9), 118.1 ($=CH_2$), 70.1 (OCH_2), 10.5 (CH_3); HRMS (ESI) calcd. for $C_{17}H_{13}Cl_2NO$ $[M]^+$ 317.0374, found 317.0368.

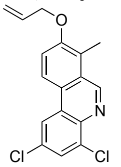

General procedure for for the synthesis of 7-allylphenanthridin-8-ols 3.

A 0.2-0.3 M solution of compound **2** in toluene under Ar was stirred at 250 °C for 45 min in the microwave reactor. The mixture was evaporated *in vacuo* and the product was purified by flash chromatography on SiO_2 eluting with CH_2Cl_2-EtOAc-hexanes (1:3:36).

7-Allyl-2,4-dichlorophenanthridin-8-ol (3a). The reaction was run in 0.13 mmol scale; yield 38 mg (95%), colorless solid, mp 250-251 °C. ^{1}H NMR (600 MHz, acetone-d_6) δ 9.57 (s, 1H, H-6), 9.34 (br d, 1H, OH), 8.65 (d, J = 8.8 Hz, 1H, H-10), 8.64 (d, J = 2.2 Hz, 1H, H-1), 7.83 (d, J = 2.2 Hz, 1H, H-3), 7.66 (d, J = 8.9 Hz, 1H, H-9), 6.19-6.12 (m, 1H, =CH), 5.09-5.07 (m, 1H, H_A in $=CH_2$), 5.06-5.05 (m, 1H, H_B in $=CH_2$), 4.08 (d, J = 5.9 Hz, 2H, CH_2); ^{13}C NMR (150 MHz, acetone-d_6) δ 156.2 (C-8), 151.9 (C-6), 138.7 (C-4a), 137.6 (=CH), 136.3 (C-4), 132.8 (C-2), 128.4 (C-3), 128.1 (C-6a or C-10b), 127.5 (C-6a or C-10b), 126.0 (C-10a), 123.5 (C-10), 122.8 (C-9), 122.2 (C-7), 121.7 (C-1), 116.0 ($=CH_2$), 29.0 (CH_2); HRMS (ESI) calcd. for $C_{16}H_{11}Cl_2NO$ $[M]^+$ 303.0218, found 303.0212.

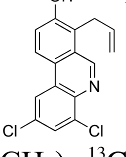

7-Allyl-4-chlorophenanthridin-8-ol (3b). The reaction was run in 0.13 mmol scale; yield 30 mg (86%), colorless solid, mp 245-246 °C. ^{1}H NMR (400 MHz, acetone-d_6) δ 9.57 (s, 1H, H-6), 9.17 (br s, 1H, OH), 8.64-8.61 (m, 2H, H-1 and H-10), 7.81 (dd, J = 7.6, 1.2 Hz, 1H, H-3), 7.64-7.61 (m, 2H, H-2 and H-9), 6.20-6.10 (m, 1H, =CH), 5.09-5.05 (m, 1H, H_A in =CH_2), 5.05-5.03 (m, 1H, H_B in =CH_2), 4.08-4.06 (m, 2H, CH_2); ^{13}C NMR (100 MHz, acetone-d_6) δ 155.5 (C-8), 151.4 (C-6), 140.1 (C-4a), 137.7 (=CH), 135.1 (C-4), 128.6 (C-3), 128.0 (C-2), 127.3 (C-10a), 127.2 (C-10b), 127.0 (C-6a), 123.2 (C-10), 122.6 (C-9), 122.1 (C-1), 122.0 (C-7), 115.9 (=CH_2), 29.0 (CH_2); HRMS (ESI) calcd. for $C_{16}H_{12}ClNO$ [M]$^+$ 269.0607, found 269.0602.

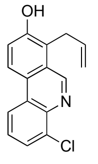

7-Allyl-4-chloro-2-nitrophenanthridin-8-ol (3c). The reaction was run in 0.11 mmol scale; yield 22 mg (63%), colorless solid, mp 249-250 °C. ^{1}H NMR (400 MHz, acetone-d_6) δ 9.69 (s, 1H, H-6), ca. 9.4 (br s, 1H, OH), 9.39 (d, J = 2.4 Hz, 1H, H-1), 8.76 (d, J = 8.9 Hz, 1H, H-10), 8.49 (d, J = 2.4 Hz, 1H, H-3), 7.73 (d, J = 8.9 Hz, 1H, H-9), 6.20-6.10 (m, 1H, =CH), 5.09-5.07 (m, 1H, H_A in =CH_2), 5.05-5.04 (m, 1H, H_B in =CH_2), 4.09-4.07 (m, 2H, CH_2); ^{13}C NMR (100 MHz, acetone-d_6) δ 156.7 (C-8), 155.0 (C-6), 146.3 (C-2), 142.7 (C-4a), 137.5 (=CH), 136.4 (C-4), 127.6 (C-6a), 126.9 (C-10a), 126.7 (C-10b), 123.7 (C-10), 123.6 (C-9), 122.7 (C-7), 122.0 (C-3), 118.3 (C-1), 116.1 (=CH_2), 28.9 (CH_2); HRMS (ESI) calcd. for $C_{16}H_{11}ClN_2O_3$ [M]$^+$ 314.0458, found 314.0452.

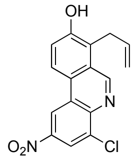

7-(But-3-en-2-yl)-2,4-dichlorophenanthridin-8-ol (3d). The reaction was run in 0.090 mmol scale; yield 23 mg (78%), colorless solid, mp 240-241 °C. ^{1}H NMR (400 MHz, acetone-d_6) δ 9.76 (s, 1H, H-6), 8.67-8.65 (m, 2H, H-1 and H-10) 7.83 (d, J = 2.1 Hz, 1H, H-3), 7.66 (d, J = 9.0 Hz, 1H, H-9), 6.52-6.44 (m, 1H, =CH), 5.27-5.21 (m, 1H, H_A in =CH_2), 5.17-5.14 (m, 1H, H_B in =CH_2), 4.85-4-78 (m, 1H, CH), 1.68 (d, J = 7.2 Hz, 3H, CH_3); ^{13}C NMR (100 MHz, acetone-d_6) δ 156.0 (C-8), 152.1 (C-6), 143.6 (=CH), 138.5 (C-4a), 136.2 (C-4), 132.7 (C-2), 128.4 (C-3), 128.2 (C-10a), 127.7 (C-6a), 127.3 (C-7), 126.6 (C-10b), 123.7 (C-10), 123.2 (C-9), 121.8 (C-1), 114.2 (=CH_2), 34.8 (CH), 19.6 (CH_3); HRMS (ESI) calcd. for $C_{17}H_{13}Cl_2NO$ [M]$^+$ 317.0374, found 317.0369.

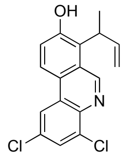

2,4-Dichloro-7-(2-methylallyl)phenanthridin-8-ol (3f) and 6,8-dichloro-2,2-dimethyl-2,3-dihydrofuro[3,2-*i*]phenanthridine (4). The reaction was run in 0.13 mmol scale; yield 36 mg (89%) **3f** and 2 mg (5%) **4**.

3f: Mp 242-243 °C, colorless solid. ^{1}H NMR (400 MHz, DMSO-d_6) δ 10.35 (s, 1H, OH), 9.43 (s, 1H, H-6), 8.73 (d, J = 2.0 Hz, 1H, H-1), 8.66 (d, J = 9.0 Hz, 1H, H-10), 7.91 (d, J = 2.0 Hz, 1H, H-3), 7.56 (d, J = 9.0 Hz, 1H, H-9), 4.73 (s, 1H, H_A in =CH_2), 4.34 (s, 1H, H_B in =CH_2), 3.87 (s, 2H, CH_2), 1.81 (s, 3H, CH_3); ^{13}C NMR (100 MHz, DMSO-d_6) δ 155.7 (C-8), 151.4 (C-6), 144.7 (C=), 136.9 (C-4a), 134.4 (C-2), 131.4 (C-4), 127.3 (C-3), 126.9 (C-10b), 126.5 (C-6a), 124.1 (C-10a), 122.9 (C-10), 122.0 (C-9), 121.1 (C-1), 120.5 (C-7), 110.9 (=CH_2), 31.5 (CH_2), 22.7 (CH_3); HRMS (ESI) calcd. for $C_{17}H_{13}Cl_2NNaO$ [M+Na]$^+$ 340.0266, found 340.0267.

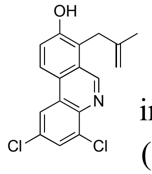

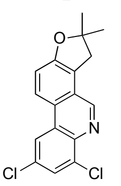

4: Mp 204-205 °C, colorless solid. ^{1}H NMR (400 MHz, DMSO-d_6) δ 9.31 (s, 1H, H-6[§]), 8.79 (d, J = 2.1 Hz, 1H, H-1), 8.73 (d, J = 8.8 Hz, 1H, H-9), 7.95 (d, J = 2.1 Hz, 1H, H-3), 7.49 (d, J = 8.8 Hz, 1H, H-10), 3.54 (s, 2H, CH_2), 1.56 (s, 6H, 2×CH_3); ^{13}C NMR (100 MHz, DMSO-d_6) δ 159.4 (C-8), 151.8 (C-6), 137.8 (C-4a), 135.1 (C-4), 132.1 (C-2), 127.9 (C-3), 127.6 (C-10a), 124.9 (C-7 or C-10b), 124.7 (C-9), 124.3 (C-7 or C-10b), 123.3 (C-6a), 121.8 (C-1), 116.5 (C-10), 89.8 (C), 28.6 (2×CH_3), the CH_2 signal was hidden under the solvent peaks. HRMS (ESI) calcd. for $C_{17}H_{13}Cl_2NO$ $[M]^+$ 317.0374, found 317.0368.

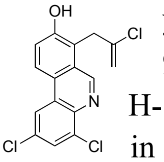

2,4-Dichloro-7-(2-chloroallyl)phenanthridin-8-ol (3g) The reaction was run in 0.10 mmol scale; yield 29 mg (83%), colorless solid, mp 264-265 °C. ^{1}H NMR (400 MHz, acetone-d_6) δ 9.57 (br s, 2H, H-6 and OH), 8.74 (d, J = 8.9 Hz, 1H, H-10), 8.68 (d, J = 2.2 Hz, 1H, H-1), 7.85 (d, J = 2.2 Hz, 1H, H-3), 7.69 (d, J = 8.9 Hz, 1H, H-9), 5.28-5.27 (m, 1H, H_A in =CH_2), 5.05-5.04 (m, 1H, H_B in =CH_2), 4.38 (s, 2H, CH_2); ^{13}C NMR (100 MHz, acetone-d_6) δ 156.8 (C-8), 151.5 (C-6), 141.71 (=C), 138.8 (C-4a), 136.3 (C-4), 133.0 (C-2), 128.6 (C-3), 127.9 (C-6a), 127.7 (C-10b), 126.1 (C-10a), 124.7 (C-10), 122.9 (C-9), 121.8 (C-1), 119.4 (C-7), 114.0 (=CH_2), 34.3 (CH_2); HRMS (ESI) calcd. for $C_{16}H_{10}Cl_3NO$ $[M]^+$ 336.9828, found 336.9822.

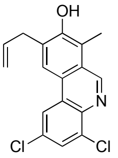

9-Allyl-2,4-dichloro-7-methylphenanthridin-8-ol (5a). The reaction was run in 0.17 mmol scale; yield 17 mg (36%) compound **1d**, 9.5 mg (18%) recovered compound **2h** and 23 mg (ca. 40%) compound **5a**, not pure. The NMR and MS data listed, strongly support the structure **5a**. ^{1}H NMR (400 MHz, acetone-d_6) δ 9.53 (s, 1H), 8.61 (d, J = 2.0 Hz, 1H), 8.46 (s, 1H), 7.79 (d, J = 2.0 Hz, 1H), 6.23-6.13 (m, 1H), 5.21-5.11 (m, 2H), 3.74 (d, J = 6.4 Hz, 2H), 2.76 (s, 3H); HRMS (ESI) calcd. for $C_{17}H_{13}Cl_2NO$ $[M+H]^+$ 318.0447, found 318.0447.

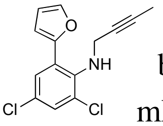

***N*-(But-2-ynyl)-2,4-dichloro-6-(furan-2-yl)aniline (7b).** Sodium hydride (ca. 60 % in mineral oil, 144 mg, ca. 3.61 mmol) was added to a stirring solution of tetrabutylammonium bromide (1.35 g, 4.07 mmol) and compound **6** (453 mg, 1.99 mmol) in dry THF (35 mL) at ambient temperature under Ar. The mixture was stirred for 15 min at 45 °C before dropwise addition of 1-bromobut-2-yne (0.22 mL, 2.6 mmol). After 23 h, the reaction was quenched with water (40 mL), extracted with CH_2Cl_2 (3×30 mL), dried ($MgSO_4$) and evaporated *in vacuo*. The product was purified by flash chromatography on SiO_2 eluting with CH_2Cl_2-hexane (1:3) followed by acetone-hexane (1:29); yield 488 mg (88%), yellow crystals, mp 65-66 °C. ^{1}H NMR (600 MHz, $CDCl_3$) δ 7.52 (d, J = 2.4 Hz, 1H, H-5), 7.48 (dd, J = 1.7, 0.5 Hz, 1H, H-5 in furyl), 7.27 (d, J = 2.4 Hz, 1H, H-3), 6.91 (dd, J = 3.4, 0.5 Hz, 1H, H-3 in furyl), 6.50 (dd, J = 3.4, 1.7 Hz, 1H, H-4 in furyl), 4.03 (br s, 1H, NH), 3.63 (q, J = 2.4 Hz, 2H, NCH_2), 1.74 (t, J = 2.4 Hz, 3H, CH_3); ^{13}C NMR (150 MHz, $CDCl_3$) δ 150.2 (C-2 in furyl), 142.5 (C-5 in furyl), 140.2 (C-1), 128.9 (C-4 or C-6), 128.3 (C-3), 127.8 (C-4 or C-6), 126.8 (C-2), 125.9 (C-5), 112.1 (C-4 in furyl), 110.1 (C-3 in furyl), 80.2 (≡$\underline{C}CH_3$), 76.3 ($CH_2\underline{C}$≡), 37.4 (NCH_2), 3.7 (CH_3); HRMS (ESI) calcd. for $C_{14}H_{11}Cl_2NO$ $[M+Na]^+$ 302.0110, found 302.0110.

[§] The same numbering system as for the simple phenanthridines is used.

2,4-Dichloro-6-(furan-2-yl)-*N*-methyl-*N*-(prop-2-ynyl)aniline (8a). Sodium hydride (ca. 60% in 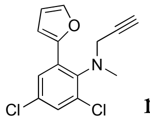 mineral oil, 133 mg, ca. 3.33 mmol) was added to a stirring solution of compound **7a** (328 mg, 1.23 mmol) in dry DMF (25 mL) at ambient temperature under Ar. The resulting mixture was stirred for 15 min at 35 °C, before dropwise addition of methyl iodide (0.25 mL, 4.0 mmol). The reaction mixture was stirred at 35 °C for 17 h quenched with sat. aq. NaCl (15 mL) and the aqueous phase was extracted with CH_2Cl_2 (3×30 mL). The combined organic phases were dried ($MgSO_4$) and evaporated *in vacuo*. The product was purified by flash chromatography on SiO_2 eluting with CH_2Cl_2-hexane (1:39); yield 286 mg (83%), colorless oil. ^{1}H NMR (300 MHz, $CDCl_3$) δ 7.73 (d, *J* = 2.5 Hz, 1H, H-3 or H-5), 7.48 (dd, *J* = 1.8, 0.6 Hz, 1H, H-5 in furyl), 7.29 (dd, *J* = 3.4, 0.6 Hz, 1H, H-3 in furyl), 7.23 (d, *J* = 2.5 Hz, 1H, H-3 or H-5), 6.51 (dd, *J* = 3.4, 1.8 Hz, 1H, H-4 in furyl), 3.89 (br s, 2H, NCH_2), 2.85 (s, 3H, NCH_3), 2.25 (t, *J* = 2.5 Hz, 1H, ≡CH); ^{13}C NMR (100 MHz, $CDCl_3$) δ 149.8 (C-2 in furyl), 142.5 (C-5 in furyl), 142.1 (C-6), 136.7 (C-2), 133.4 (C-5), 132.2 (C-4), 128.7 (C-3), 125.4 (C-1), 112.5 (C-4 in furyl), 111.7 (C-3 in furyl), 80.8 (C≡), 72.3 (≡CH), 43.8 (NCH_2), 38.6 (NCH_3); HRMS (ESI) calcd. for $C_{14}H_{11}Cl_2NO$ $[M+H]^+$ 280.0290, found 280.0291.

***N*-(But-2-ynyl)-2,4-dichloro-6-(furan-2-yl)-*N*-methylaniline (8b).** Sodium hydride (ca. 60% in 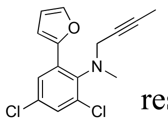 mineral oil, 177 mg, ca. 4.42 mmol) was added to a stirring solution of compound **7b** (454 mg, 1.62 mmol) in dry DMF (30 mL) at ambient temperature under Ar. The resulting mixture was stirred for 15 min at 35 °C before dropwise addition of methyl iodide (0.30 mL, 4.9 mmol). The reaction mixture was stirred at 35 °C for 18 h quenched with sat. aq. NaCl (20 mL) and the aqueous phase was extracted with CH_2Cl_2 (3×50 mL). The combined organic phases were dried ($MgSO_4$) and evaporated *in vacuo*. The product was purified by flash chromatography on SiO_2 eluting with CH_2Cl_2-hexane (1:19); yield 390 mg (82%), yellow oil. ^{1}H NMR (600 MHz, $CDCl_3$) δ 7.70 (d, *J* = 2.5 Hz, 1H, H-5), 7.46 (dd, *J* = 1.8, 0.7 Hz, 1H, H-5 in furyl), 7.27 (dd, *J* = 3.5, 0.7 Hz, 1H, H-3 in furyl), 7.20 (d, *J* = 2.5 Hz, 1H, H-3), 6.49 (dd, *J* = 3.5, 1.8 Hz, 1H, H-4 in furyl), 3.80 (br s, 2H, NCH_2), 2.81 (s, 3H, NCH_3), 1.79 (t, *J* = 2.5 Hz, 3H, CH_3); ^{13}C NMR (150 MHz, $CDCl_3$) δ 150.0 (C-2 in furyl), 142.5 (C-5 or C-5 in furyl), 142.4 (C-5 or C-5 in furyl), 136.7 (C-2 or C-6), 133.4 (C-2 or C-6), 131.8 (C-4), 128.8 (C-3), 125.4 (C-1), 112.4 (C-4 in furyl), 111.6 (C-3 in furyl), 79.9 (≡$\underline{C}CH_3$), 76.0 ($CH_2\underline{C}$≡), 44.1 (NCH_2), 38.5 (NCH_3), 3.7 (CH_3); HRMS (ESI) calcd. for $C_{15}H_{14}Cl_2NO$ $[M+H]^+$ 294.0447, found 294.0447.

2,4-Dichloro-5-methyl-5,6-dihydrophenanthridin-8-ol (9a). A stirring solution of compound **8a** 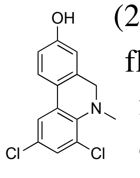 (237 mg, 0.847 mmol) and aq. HCl (0.2 M, 5 drops) in degassed CH_3CN (20 mL) was flushed with Ar for 15 min in a reactor tube and heated in the microwave oven at 180 °C for 180 min. The solvent was removed *in vacuo* and the product was purified by flash chromatography on SiO_2 eluting with CH_3OH-CH_2Cl_2 (1:49); yield 169 mg (71%), yellow waxy material. ^{1}H NMR (400 MHz, DMSO-d_6) δ 9.81 (s, 1H, OH), 7.79 (d, *J* = 2.3 Hz, 1H, H-1), 7.72 (d, *J* = 8.4 Hz, 1H, H-10), 7.40 (d, *J* = 2.3 Hz, 1H, H-3), 6.77 (dd, *J* = 8.4, 2.5 Hz, 1H, H-9), 6.71 (d, *J* = 2.5 Hz, 1H, H-7), 4.00 (s, 2H, NCH_2), 2.51 (s, 3H, NCH_3); ^{13}C NMR (100 MHz, DMSO-d_6) δ 158.6 (C-8), 142.4 (C-4a), 134.2 (C-6a), 132.6 (C-10b), 129.7 (C-2), 128.6 (C-4), 126.9 (C-3), 125.2 (C-10), 121.6 (C-1), 120.7 (C-10a), 114.9 (C-9), 113.5 (C-7), 53.7 (NCH_2), 40.1(NCH_3); HRMS (ESI) calcd. for $C_{14}H_{12}Cl_2NO$ $[M+H]^+$ 280.0290, found 280.0291.

2,4-Dichloro-5,7-dimethyl-5,6-dihydrophenanthridin-8-ol (9b). A stirring solution of compound

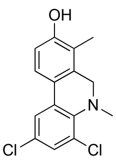

8b (196 mg, 0.666 mmol) and aq. HCl (0.2 M, 2 drops) in degassed CH_3CN (13 mL) was flushed with Ar for 15 min in a reactor tube and heated in the microwave oven at 200 °C for 7 h. The solvent was removed *in vacuo* and the product was purified by flash chromatography on SiO_2 eluting with CH_3OH-CH_2Cl_2 (1:199); yield 118 mg (60%), orange crystals, mp 148-151 °C. ^{1}H NMR (400 MHz, DMSO-d_6) δ 9.70 (s, 1H, OH), 7.75 (d, *J* = 2.4 Hz, 1H, H-1 or H-3), 7.56 (d, *J* = 8.4 Hz, 1H, H-10), 7.39 (d, *J* = 2.4 Hz, 1H, H-1 or H-3), 6.83 (d, *J* = 8.4 Hz, 1H, H-9), 4.08 (s, 2H, NCH_2), 2.52 (s, 3H, NCH_3), 2.13 (s, 3H, CH_3); ^{13}C NMR (100 MHz, DMSO-d_6) δ 156.5 (C-8), 142.1 (C-4), 133.1 (C-6a or C10a), 132.9 (C-6a or C10a), 129.5 (C-4a), 128.4 (C-10b), 126.7 (C-1 or C-3), 122.2 (C-9), 121.7 (C-1 or C-3), 120.9 (C-2 or C-7), 120.7 (C-2 or C-7), 113.8 (C-10), 50.7 (NCH_2), 40.1 (NCH_3), 10.7 (CH_3); HRMS (ESI) calcd. for $C_{15}H_{14}Cl_2NO$ [*M*+H]$^+$ 294.0447, found 294.0446.

General procedure for the synthesis of 8-allyloxydihydrophenanthridines 10.
Compound **9** (0.16 mmol) and K_2CO_3 (44 mg, 0.32 mmol) in dry DMF (10 mL) was stirred under Ar for 15 min, before the allylic bromide (0.32 mmol) was added. After stirring for additional 90 min, water (25 mL) was added and the resulting mixture was extracted with EtOAc (3×25 mL). The combined organic phases were dried ($MgSO_4$), filtered through a plug of SiO_2, eluting with several portions of EtOAc and the solvent was evaporated *in vacuo.* The product was stored under inert atmosphere to prevent oxidation at C-6.

8-(Allyloxy)-2,4-dichloro-5-methyl-5,6-dihydrophenanthridine (10a). Yield 51 mg (99%),

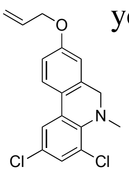

yellow oil. ^{1}H NMR (400 MHz, $CDCl_3$) δ 7.60 (d, *J* = 8.5 Hz, 1H, H-10), 7.59 (d, *J* = 2.2 Hz, 1H, H-1), 7.28 (d, *J* = 2.2 Hz, 1H, H-3), 6.93 (dd, *J* = 8.5, 2.6 Hz, 1H, H-9), 6.80 (d, *J* = 2.6, 1H, H-7), 6.08 (ddt, *J* = 17.2, 10.5, 5.3 Hz, 1H, CH=), 5.44 (ddt, *J* = 17.2, 1.5, 1.5 Hz, 1H, H_A in =CH_2), 5.32 (ddt, *J* = 10.5, 1.5, 1.5 Hz, 1H, H_B in =CH_2), 4.59 (dt, *J* = 5.3, 1.5 Hz, 2H, CH_2), 4.09 (s, 2H, H-6), 2.61 (s, 3H, CH_3); ^{13}C NMR (100 MHz, $CDCl_3$) δ 159.6 (C-8), 142.8 (C-4a), 134.4 (C-10a), 133.1 (CH=), 132.4 (C-2), 130.7 (C-4), 129.9 (C-10b), 128.1 (C-3), 124.8 (C-10), 123.4 (C-6a), 122.0 (C-1), 118.1 (=CH_2), 114.4 (C-9), 113.2 (C-7), 69.1 (OCH_2), 55.2 (C-6), 40.5 (CH_3); HRMS (ESI) calcd. for $C_{17}H_{16}Cl_2NO$ [*M*+H]$^+$ 320.0603, found 320.0603.

2,4-Dichloro-5-methyl-8-[(2-methylallyl)oxy]-5,6-dihydrophenanthridine (10b). The reaction

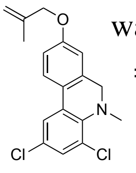

was run for 2.5 h; yield 52 mg (98%), yellow oil. ^{1}H NMR (400 MHz, $CDCl_3$) δ 7.59 (d, *J* = 8.6 Hz, 1H, H-10), 7.58 (d, *J* = 2.3 Hz, 1H, H-1), 7.28 (d, *J* = 2.3 Hz, 1H, H-3), 6.93 (dd, *J* = 8.6, 2.6 Hz, 1H, H-9), 6.80 (d, *J* = 2.6, 1H, H-7), 5.12 (s, 1H, H_A in =CH_2), 5.02 (s, 1H, H_B in =CH_2), 4.48 (s, 2H, OCH_2), 4.08 (s, 2H, H-6), 2.61 (s, 3H, NCH_3), 1.85 (s, 3H, CH_3); ^{13}C NMR (100 MHz, $CDCl_3$) δ 159.8 (C-8), 142.8 (C-4a), 140.7 (C=), 134.4 (C-10a), 132.4 (C-2), 130.7 (C-4), 129.9 (C-10b), 128.1 (C-3), 124.8 (C-10), 123.3 (C-6a), 122.0 (C-1), 114.4 (C-9), 113.2 (C-7), 113.1 (=CH_2), 72.0 (OCH_2), 55.2 (C-6), 40.5 (NCH_3), 19.6 (CH_3); HRMS (ESI) calcd. for $C_{18}H_{18}Cl_2NO$ [*M*+H]$^+$ 334.0760, found 334.0760.

8-(Allyloxy)-2,4-dichloro-5,7-dimethyl-5,6-dihydrophenanthridine (10c). The reaction was run for 2 h in 0.26 mmol scale; yield 85 mg (99%), amber oil.

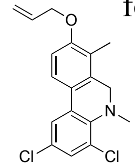

^{1}H NMR (400 MHz, $CDCl_3$) δ δ 7.58 (d, *J* = 2.3 Hz, 1H, H-1), 7.49 (d, *J* = 8. 6 Hz, 1H, H-10), 7.28 (d, *J* = 2.3 Hz, 1H, H-3), 6.85 (d, *J* = 8.6, 1H, H-9), 6.10 (ddt, *J* = 17.4, 10.4, 5.1 Hz, 1H, CH=), 5.46 (ddt, *J* = 17.4, 1.5, 1.5 Hz, 1H, H_A in $=CH_2$), 5.31 (ddt, *J* = 10.4, 1.5, 1.5 Hz, 1H, H_B in $=CH_2$), 4.60 (dt, *J* = 5.1, 1.5 Hz, 2H, OCH_2), 4.17 (s, 2H, H-6), 2.61 (s, 3H, NCH_3), 2.26 (s, 3H, CH_3); ^{13}C NMR (100 MHz, $CDCl_3$) *δ* 157.6 (C-8), 142.6 (C-4a), 133.4 (CH=), 133.2 (C-10a), 133.0 (C-2), 130.5 (C-4), 129.7 (C-10b), 127.9 (C-3), 124.0 (C-7), 123.3 (C-6a), 122.2 (C-1), 121.9 (C-10), 117.5 ($=CH_2$), 110.6 (C-9), 69.2 (OCH_2), 51.7 (C-6), 40.6 (NCH_3), 11.2 (CH_3); HRMS (ESI) calcd. for $C_{18}H_{18}Cl_2NO$ $[M+H]^+$ 334.0760, found 334.0759.

2,4-Dichloro-5,7-dimethyl-8-[(2-methylallyl)oxy]-5,6-dihydrophenanthridine (10d). The reaction was run for 2 h; yield 30 mg (55%), colorless wax.

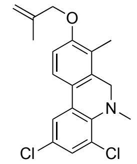

^{1}H NMR (400 MHz, $CDCl_3$) *δ* 7.58 (d, *J* = 2.0 Hz, 1H, H-1), 7.48 (d, *J* = 8.5 Hz, 1H, H-10), 7.26 (d, *J* = 2.0 Hz, 1H, H-3), 6.85 (d, *J* = 8.5, 1H, H-9), 5.14 (s, 1H, H_A in $=CH_2$), 5.02 (s, 1H, H_B in $=CH_2$), 4.48 (s, 2H, OCH_2=), 4.17 (s, 2H, H-6), 2.61 (s, 3H, NCH_3), 2.27 (s, 3H, $ArCH_3$), 1.87 (s, 3H, $=CCH_3$); ^{13}C NMR (100 MHz, $CDCl_3$) *δ* 157.7 (C-8), 142.6 (C-4a), 141.0 (C=), 133.1 (C-10a), 133.0 (C-2), 130.5 (C-4), 129.7 (C-10b), 127.9 (C-3), 123.9 (C-7), 123.2 (C-6a), 122.1 (C-1), 121.9 (C-10), 112.7 ($=CH_2$), 110.5 (C-9), 72.0 (O), 51.7 (C-6), 40.7 (NCH_3), 19.6 ($=C\underline{C}H_3$), 11.2 ($ArCH_3$); HRMS (ESI) calcd. for $C_{19}H_{20}Cl_2NO$ $[M+H]^+$ 348.0916, found 348.0916.

7-Allyl-2,4-dichloro-5-methyl-5,6-dihydrophenanthridin-8-ol (11a), 9-allyl-2,4-dichloro-5-methyl-5,6-dihydrophenanthridin-8-ol (12a) and 7-allyl-2,4-dichloro-8-hydroxy-5-methylphenanthridin-6(5*H*)-one (13a). A solution of compound **10a** (143 mg, 0.45 mmol) in toluene (6 mL) under Ar was stirred at 250 °C for 45 min in the microwave reactor. The mixture was evaporated *in vacuo* and the products were separated by flash chromatography on SiO_2 eluting with EtOAc-hexane (1:19), compound **13a** was purified further by flash chromatography on SiO_2 eluting with acetone-hexane (1:6).

11a: Yield 63 mg (44%), yellow solid, mp 161-162 °C.

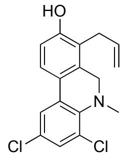

^{1}H NMR (400 MHz, acetone-d_6) δ 7.70 (d, *J* = 2.0 Hz, 1H, H-1), 7.60 (d, *J* = 8.5 Hz, 1H, H-10), 7.29 (d, *J* = 2.0 Hz, 1H, H-3), 6.96 (d, *J* = 8.5 Hz, 1H, H-9), 5.98 (ddt, *J* = 16.7, 10.0, 5.9 Hz, 1H, CH=), 5.02 (dd, *J* = 16.7, 1.6 Hz, 1H, H_A in $=CH_2$), 4.99 (dd, *J* = 10.0, 1.6 Hz, H_B in $=CH_2$), 4.12 (s, H-6), 3.54 (d, *J* = 5.9 Hz, 2H, CH_2), 2.56 (s, 3H, CH_3); ^{13}C NMR (100 MHz, acetone-d_6) δ 157.2 (C-8), 143.5 (C-4a), 137.4 (CH=), 134.6 (C-10a), 134.3 (C-2), 131.0 (C-4), 129.8 (C-10b), 127.9 (C-3), 124.1 (C-7), 123.8 (C-10), 122.9 (C-6a), 122.6 (C-1), 115.2 (C-9), 115.1 ($=CH_2$), 51.7 (C-6), 40.4 (CH_3), 29.9 (CH_2); HRMS (ESI) calcd. for $C_{17}H_{16}Cl_2NO$ $[M+H]^+$ 320.0603, found 320.0603.

12a: Yield 48 mg (33%), orange solid, mp 124-126 °C.

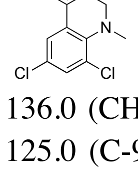

^{1}H NMR (400 MHz, $CDCl_3$) δ 7.58 (d, *J* = 2.3 Hz, 1H, H-1), 7.43 (s, 1H, H-10), 7.27 (d, *J* = 2.3 Hz, 1H, H-3), 6.70 (s, 1H, H-7), 6.06 (ddt, *J* = 16.2, 10.4, 6.2 Hz, 1H, CH=), 5.22 (ddt, *J* = 16.2, 2.0, 1.5 Hz, 1H, H_A in $=CH_2$), 5.21 (ddt, *J* = 10.4, 2.0, 1.5 Hz, H_B in $=CH_2$), 4.04 (s, 2H, H-6), 3.47 (br d, *J* = 6.2 Hz, 2H, CH_2), 2.60 (s, 3H, CH_3); ^{13}C NMR (100 MHz, $CDCl_3$) δ 155.1 (C-8), 142.6 (C-4a), 136.0 (CH=), 132.7 (C-10a), 132.2 (C-2), 130.6 (C-4), 129.7 (C-10b), 127.9 (C-3), 125.5 (C-10), 125.0 (C-9), 123.2 (C-6a), 121.8 (C-1), 116.9 ($=CH_2$), 114.2 (C-7), 54.4 (C-6), 40.4 (CH_3), 35.1 (CH_2); HRMS (ESI) calcd. for $C_{17}H_{16}Cl_2NO$ $[M+H]^+$ 320.0603, found 320.0603.

13a: Yield 6.8 mg (5%), yellow solid, mp 168-170 °C. ^{1}H NMR (400 MHz, acetone-d_6) δ ca. 9.2 (rr s, 1H, OH), 8.24 (d, J = 8.9 Hz, 1H, H-10), 8.22 (d, J = 2.3 Hz, 1H, H-1), 7.51 (d, J = 2.3 Hz, 1H, H-3), 7.41 (d, J = 8.9 Hz, 1H, H-9), 6.12 (ddt, J = 17.2, 10.1, 6.4 Hz, 1H, CH=), 5.07 (ddt, J = 17.2, 1.4, 1.4 Hz, 1H, H_A in =CH_2), 4.89 (ddt, J = 10.04, 1.4, 1.4 Hz, H_B in =CH_2), 4.23 (ddd, J = 6.4, 1.4, 1.4 Hz, 2H, OCH_2), 3.77 (s, 3H, CH_3); ^{13}C NMR (100 MHz, acetone-d_6) δ 163.9 (C-6), 157.9 (C-8), 138.5 (CH=), 135.9 (C-4a), 130.5 (C-3), 129.3 (C-10a), 128.6 (C-10b), 126.6 (C-7), 126.0 (C-2), 125.8 (C-6a), 123.2 (C-10), 122.7 (C-4), 122.3 (C-1), 121.6 (C-9), 114.7 (=CH_2), 38.9 (CH_3), 31.5 (CH_2); HRMS (ESI) calcd. for $C_{17}H_{13}Cl_2NNaO_2$ $[M+Na]^+$ 356.0216, found 356.0215.

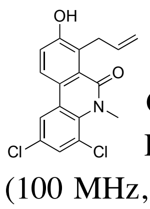

2,4-Dichloro-5-methyl-7-(2-methylallyl)-5,6-dihydrophenanthridin-8-ol (11b), 2,4-dichloro-5-methyl-9-(2-methylallyl)-5,6-dihydrophenanthridin-8-ol (12b) and 6,8-dichloro-2,2,5-trimethyl-2,3,4,5-tetrahydrofuro[3,2-*i*]phenanthridine (14b). A solution of compound **10b** (50 mg, 0.15 mmol) in toluene (6 mL) under Ar was stirred at 250 °C for 45 min in the microwave reactor. The mixture was evaporated *in vacuo* and the products were separated by flash chromatography on SiO_2 eluting with EtOAc-hexane (1:19 → 4:1).

11b: Yield 15 mg (30%), orange solid, mp 104-105 °C. ^{1}H NMR (400 MHz, $CDCl_3$) δ 7.57 (d, J = 2.3 Hz, 1H, H-1), 7.48 (d, J = 8.4 Hz, 1H, H-10), 7.27 (d, J = 2.3 Hz, 1H, H-3), 6.85 (d, J = 8.4 Hz, 1H, H-9), 4.88 (s, 1H, H_A in =CH_2), 4.63 (s, 1H, H_B in =CH_2), 4.11 (s, 2H, H-6), 3.43 (s, 2H, CH_2), 2.56 (s, 3H, NCH_3), 1.82 (s, 3H, CH_3); ^{13}C NMR (100 MHz, $CDCl_3$) δ 155.3 (C-8), 143.6 (C=), 142.3 (C-4a), 133.9 (C-10a), 132.8 (C-2), 130.4 (C-4), 129.7 (C-10b), 127.9 (C-3), 123.6 (C-6a), 123.1 (C-10), 122.9 (C-7), 122.0 (C-1), 115.0 (C-9), 111.8 (=CH_2), 51.4 (C-6), 40.2 (NCH_3), 33.7 (CH_2), 22.6 (CH_3); HRMS (ESI) calcd. for $C_{18}H_{18}Cl_2NO$ $[M+H]^+$ 334.0760, found 334.0760.

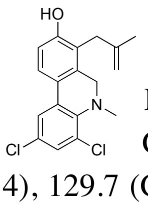

12b: Yield 12 mg (24%), orange solid, mp 122-124 °C. ^{1}H NMR (400 MHz, $CDCl_3$) δ 7.58 (d, J = 2.3 Hz, 1H, H-1), 7.41 (s, 1H, H-10), 7.27 (d, J = 2.3 Hz, 1H, H-3), 6.72 (s, 1H, H-7), 4.97 (s, 1H, H_A in =CH_2), 4.90 (s, 1H, H_B in =CH_2), 4.05 (s, 2H, H-6), 3.44 (s, 2H, CH_2), 2.61 (s, 3H, NCH_3), 1.78 (s, 3H, CH_3); ^{13}C NMR (100 MHz, $CDCl_3$) δ 155.7 (C-8), 144.4 (C=), 142.7 (C-4a), 132.9 (C-10a), 132.2 (C-2), 130.6 (C-4), 129.7 (C-10b), 127.9 (C-3), 126.0 (C-10), 124.4 (C-9), 123.1 (C-6a), 121.7 (C-1), 114.5 (C-7), 112.8 (=CH_2), 54.5 (C-6), 40.4 (CH_3), 40.0 (CH_2), 22.1 (CH_3); HRMS (ESI) calcd. for $C_{18}H_{18}Cl_2NO$ $[M+H]^+$ 334.0760, found 334.0759.

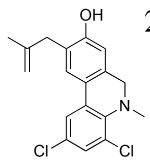

14b: Yield 5.9 mg (11%), off-white solid, mp 99-101 °C. ^{1}H NMR (400 MHz, $CDCl_3$) δ 7.98 (d, J = 2.4 Hz, 1H, H-1), 7.95 (d, J = 8.7 Hz, 1H, H-10), 7.41 (d, J = 2.4 Hz, 1H, H-3), 7.14 (d, J = 8.7 Hz, 1H, H-9), 3.85 (s, 3H, NCH_3), 3.68 (s, 2H, CH_2), 1.53 (s, 6H, 2×CH_3); ^{13}C NMR (100 MHz, $CDCl_3$) δ 163.6 (C-6), 160.5 (C-8), 134.7 (C-4a), 130.1 (C-3), 128.38 (C-2), 128.36 (C-10a), 125.1 (C-7), 125.0 (C-10b), 123.5 (C-6a), 123.0 (C-10), 122.5 (C-4), 121.1 (C-1), 115.1 (C-9), 88.9 ($\underline{C}(CH_3)_2$), 44.7 (CH_2), 38.3 (NCH_3), 28.3 (2×CH_3); HRMS (ESI) calcd. for $C_{18}H_{15}Cl_2NNaO_2$ $[M+Na]^+$ 370.0378, found 370.0378.

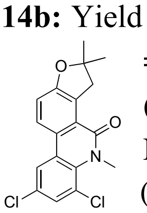

References

12. a) M. L. Read, A. Krapp, P. O. Miranda, L.-L. Gundersen, *Tetrahedron* **2012**, *68*, 1869-1885; b) M. L. Read, L.-L. Gundersen, *J. Org. Chem.* **2013**, *78*, 1311-1316; c) H. S. Gulbrandsen, M. Hennum, M. Osheka, M. L. Read, L.-L. Gundersen, *Eur. J. Org. Chem.* **2014**, 8182-8190.
13. H. S. Gulbrandsen, H. Serigstad, M. L. Read, I. Joos, L.-L. Gundersen, *Eur. J. Org. Chem.* **2019**, 6044-6052.
21. a) F. Neese, *WIREs Computational Molecular Science* **2012**, *2*, 73-78; b) F. Neese, F. Wennmohs, U. Becker, C. Riplinger, *J. Chem. Phys.* **2020**, *152*, e224108.
22. a) A. Becke, *J. Chem. Phys.* **1993**, 98, 5648-5652; b) C. Lee, W. Yang, R. Parr, *Phys. Rev. B* **1988**, *37*, 785-789.
23. E. Caldeweyher, C. Bannwarth, S. Grimme, *J. Chem. Phys*. **2017**, *147*, e034112.
24. F. Weigend, R. Ahlrichs, *Phys. Chem. Chem. Phys.* **2005**, *7*, 3297-3305.
25. F. Weigend, *Phys. Chem. Chem. Phys.* **2006**, *8*, 1057-1065.
26. F. Neese, Wennmohs, A. Hansen, U. Becker, *Chem. Phys* **2009**, *356*, 98-109.
27. a) G. Henkelman, B. Uberuaga, H. Jónsson, *J. Chem. Phys.* **2000**, *113*, 9901-9904; b) G. Henkelman, H. Jónsson, *J. Chem. Phys.* **2000**, *113*, 9978-9985; c) S. Smidstrup, A. Pedersen, K. Stokbro, H. Jónsson, *J. Chem. Phys.* 2**014**, *140*, e214106.

^{1}H NMR and ^{13}C NMR Spectra of Novel Compounds

4-Chlorophenanthridin-8-ol (1b).

^{1}H NMR (400 MHz, DMSO-d_6)

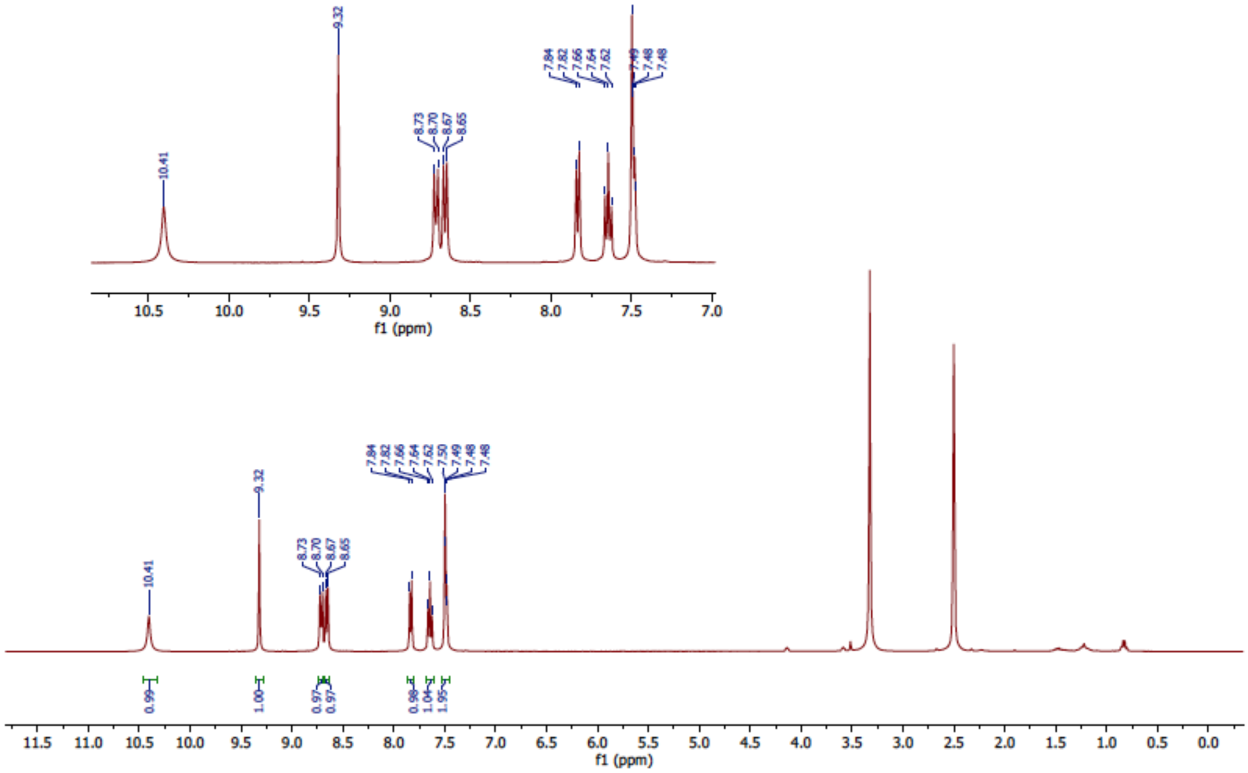

^{13}C NMR (100 MHz, DMSO-d_6)

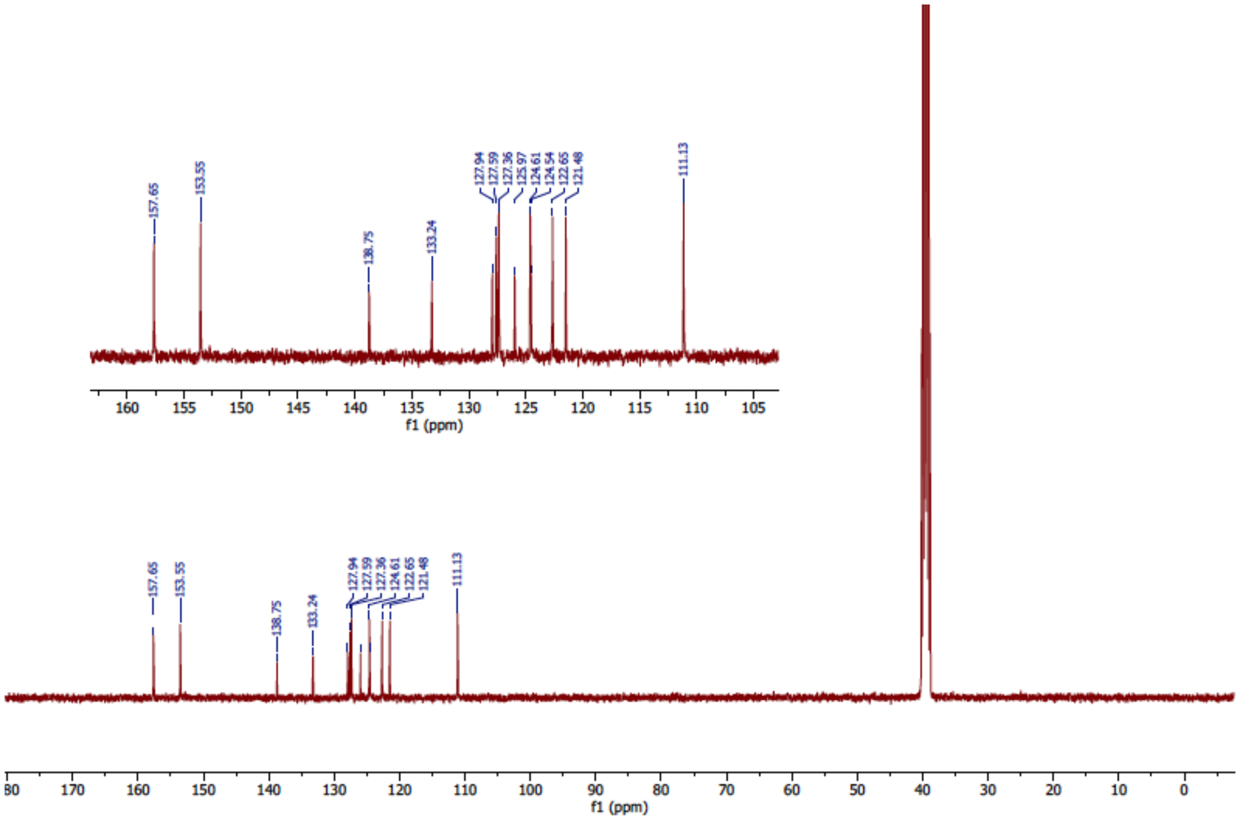

8-(Allyloxy)-2,4-dichlorophenanthridine (2a).

^{1}H NMR (600 MHz, acetone-d_6)

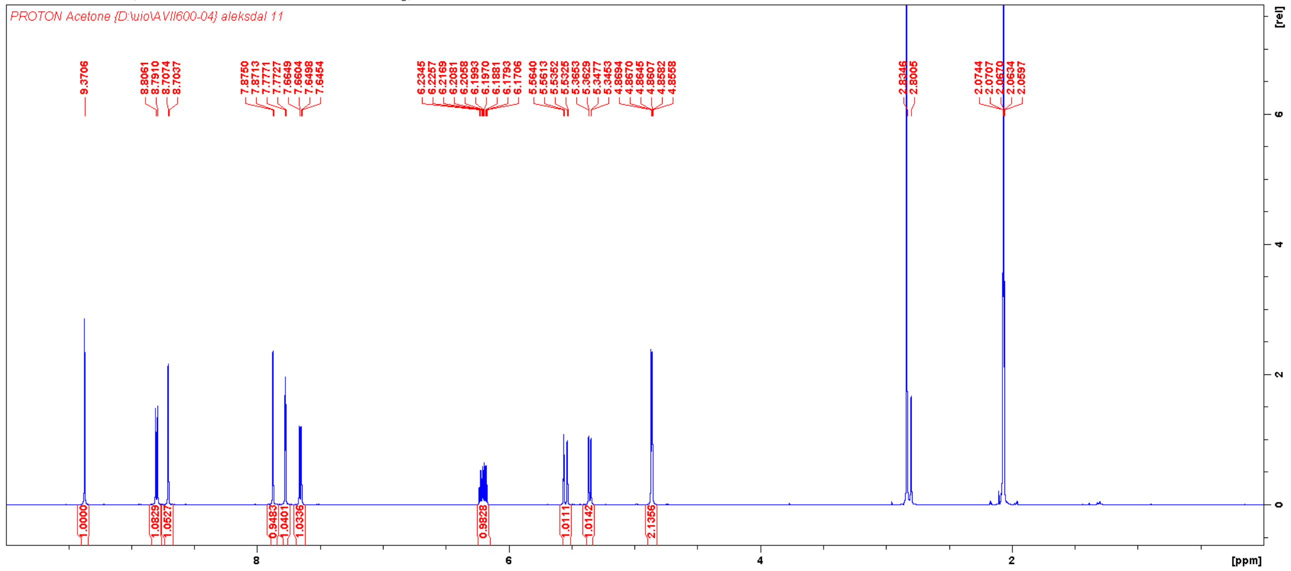

^{13}C NMR (150 MHz, acetone-d_6)

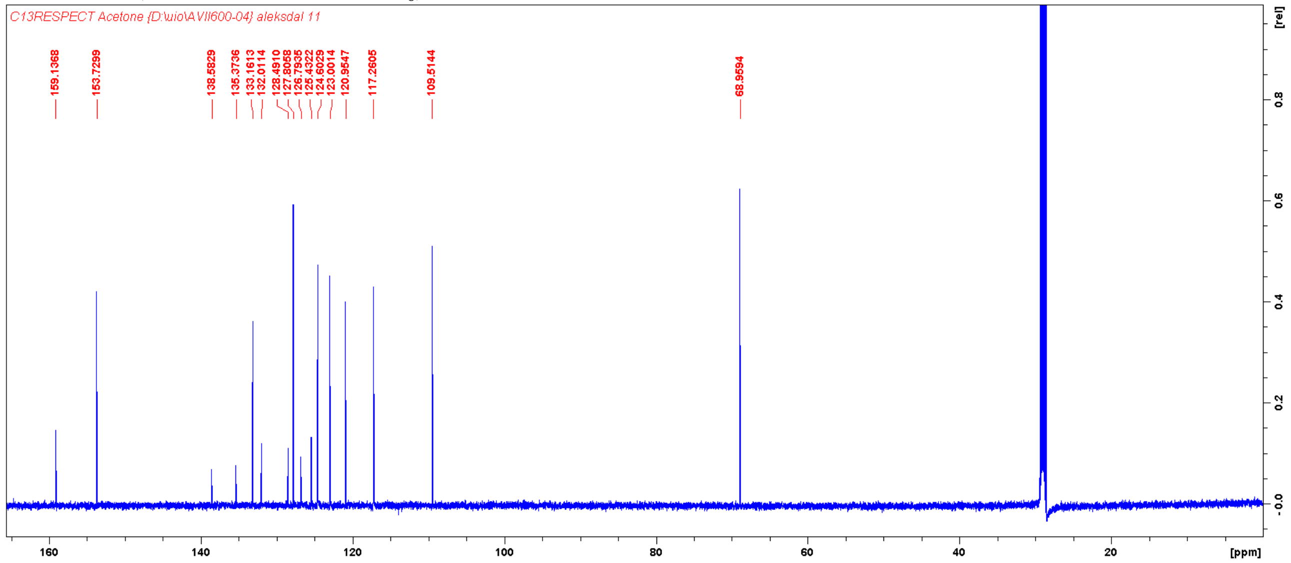

8-(Allyloxy)-4-chlorophenanthridine (2b).

^{1}H NMR (600 MHz, $CDCl_3$)

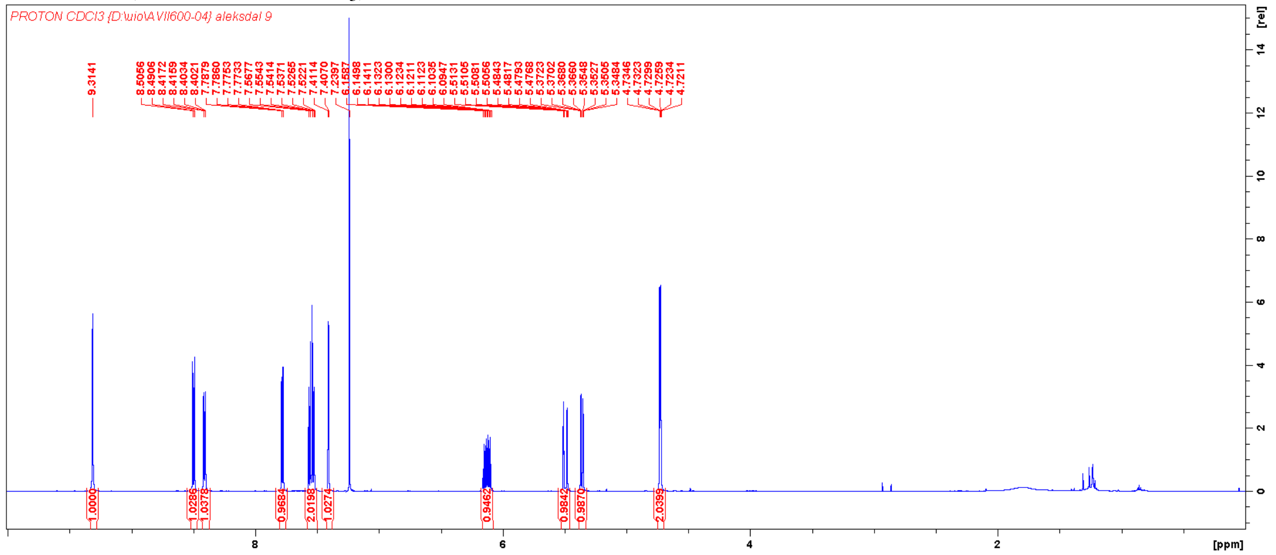

^{13}C NMR (150 MHz, $CDCl_3$)

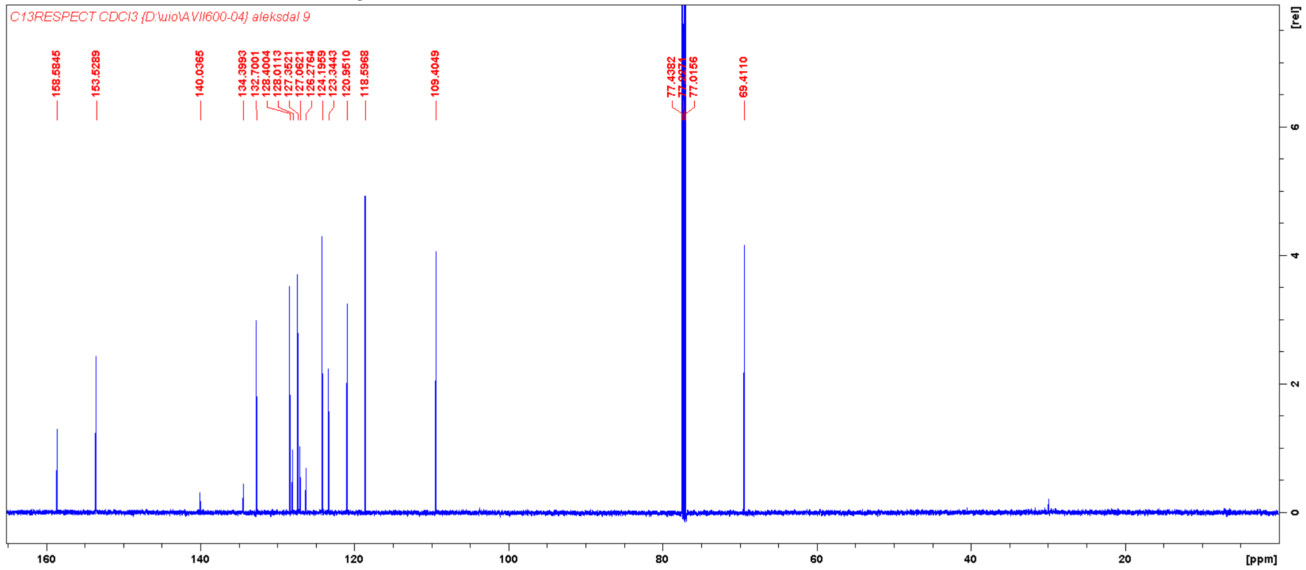

8-(Allyloxy)-4-chloro-2-nitrophenanthridine (2c).

^{1}H NMR (400 MHz, $CDCl_3$)

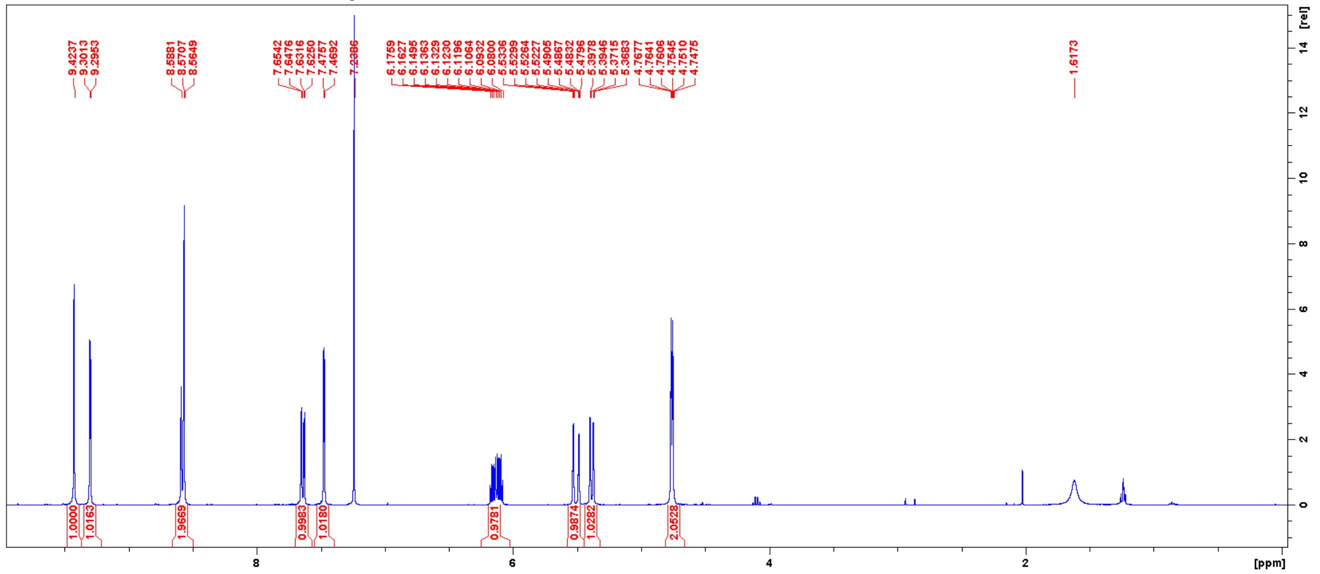

^{13}C NMR (100 MHz, $CDCl_3$)

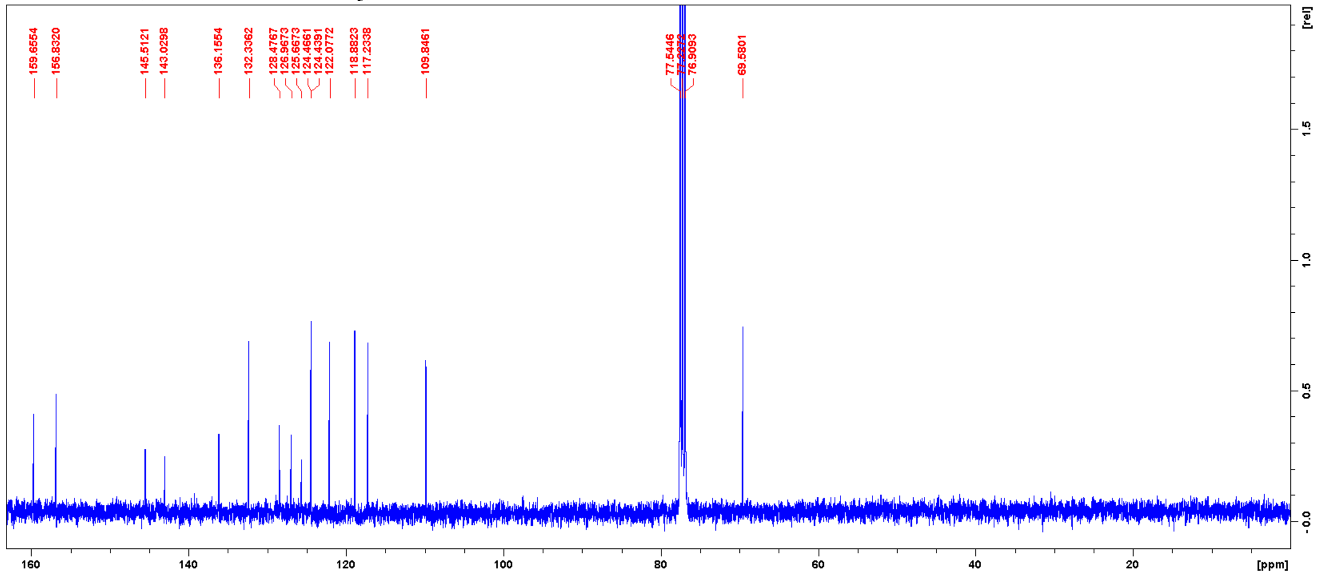

8-(But-2-en-1-yloxy)-2,4-dichlorophenanthridine (2d).

^{1}H NMR (600 MHz, acetone-d_6)

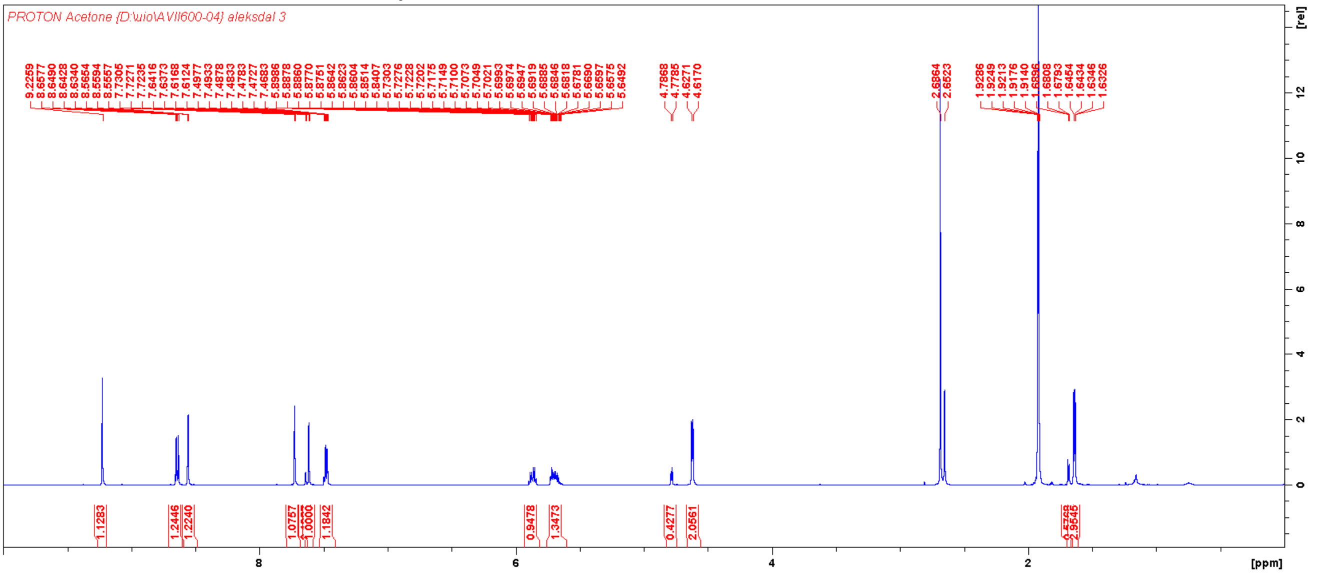

^{13}C NMR (150 MHz, acetone-d_6)

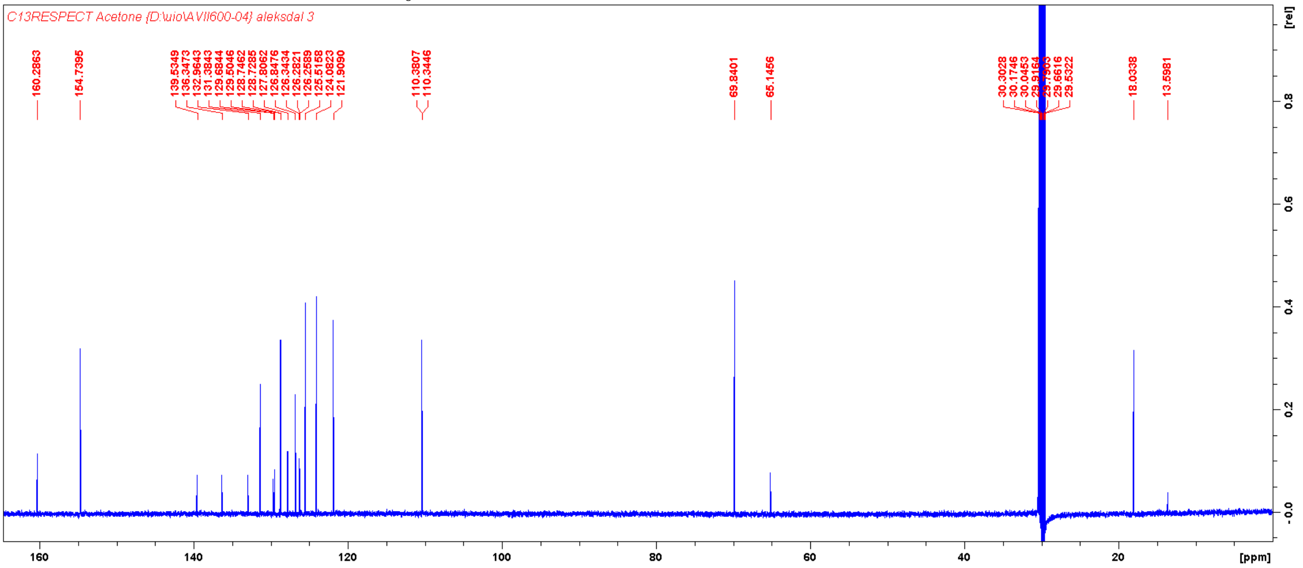

2,4-Dichloro-8-[(3-methylbut-2-en-1-yl)oxy]phenanthridine (2e).

^{1}H NMR (600 MHz, $CDCl_3$)

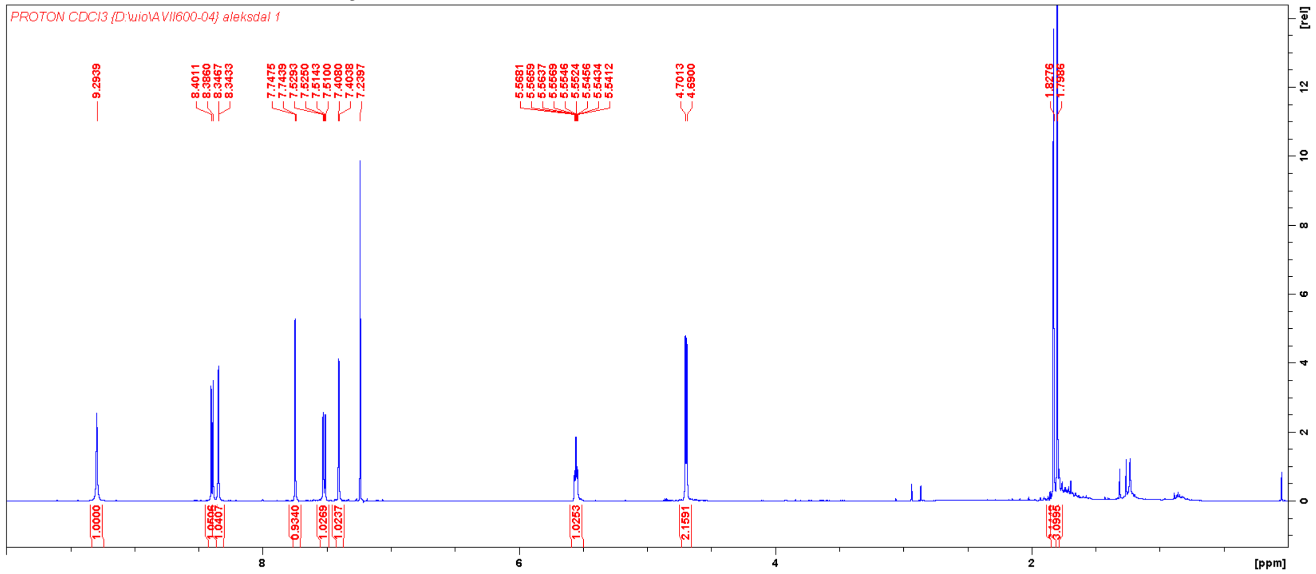

^{13}C NMR (150 MHz, $CDCl_3$)

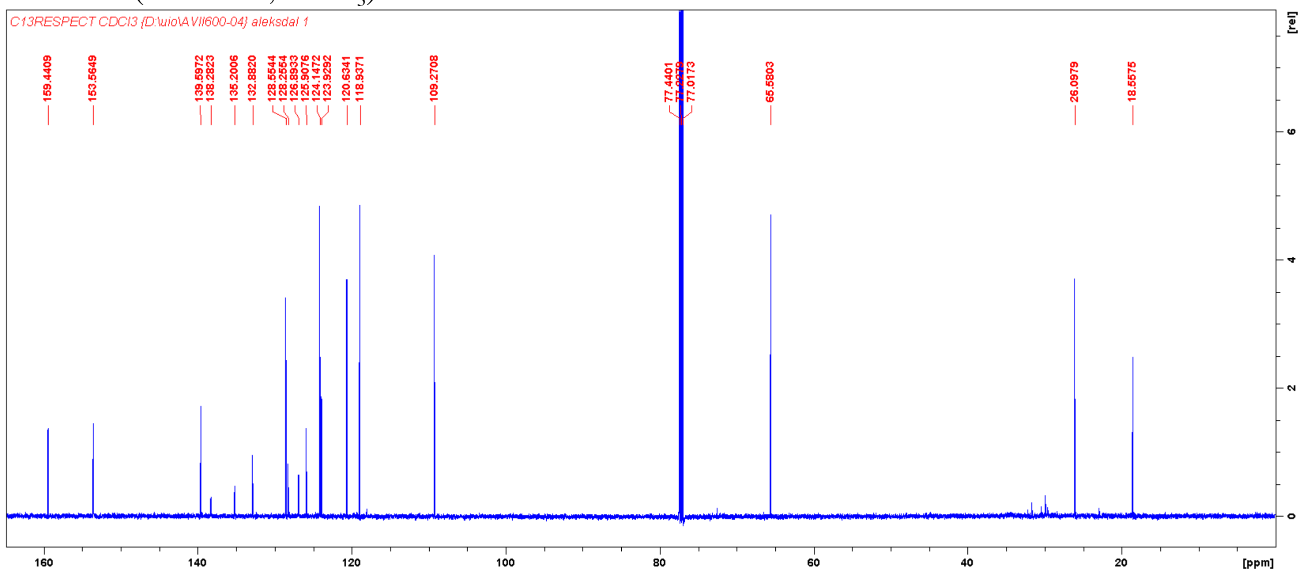

2,4-Dichloro-8-[(2-methylallyl)oxy]phenanthridine (2f).

^{1}H NMR (600 MHz, $CDCl_3$)

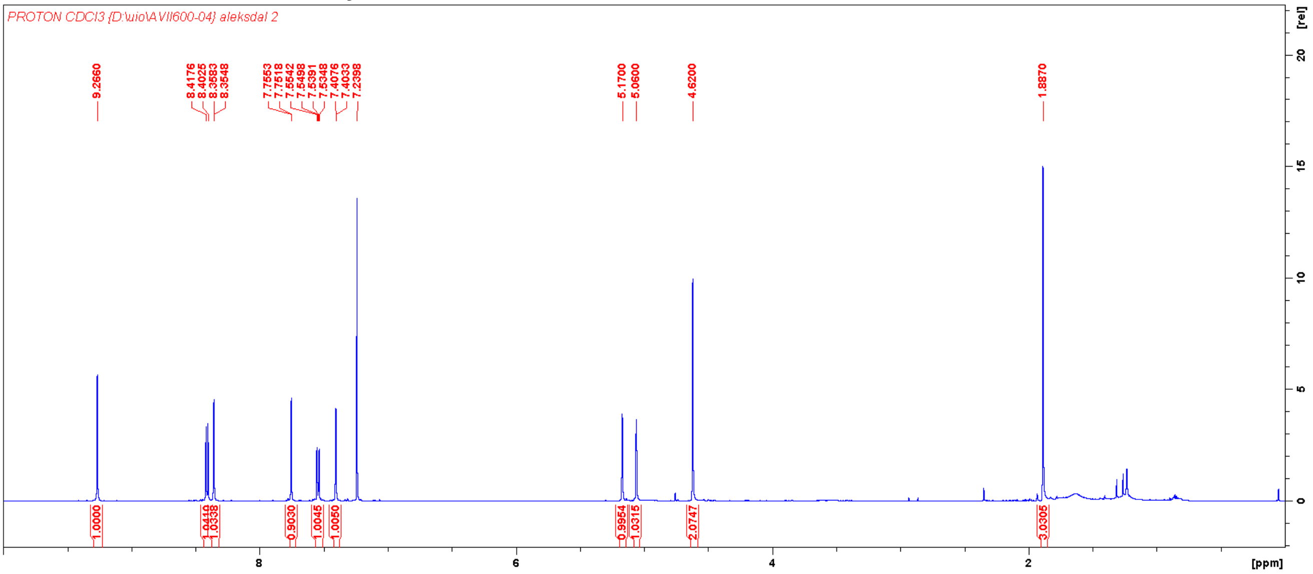

^{13}C NMR (150 MHz, $CDCl_3$)

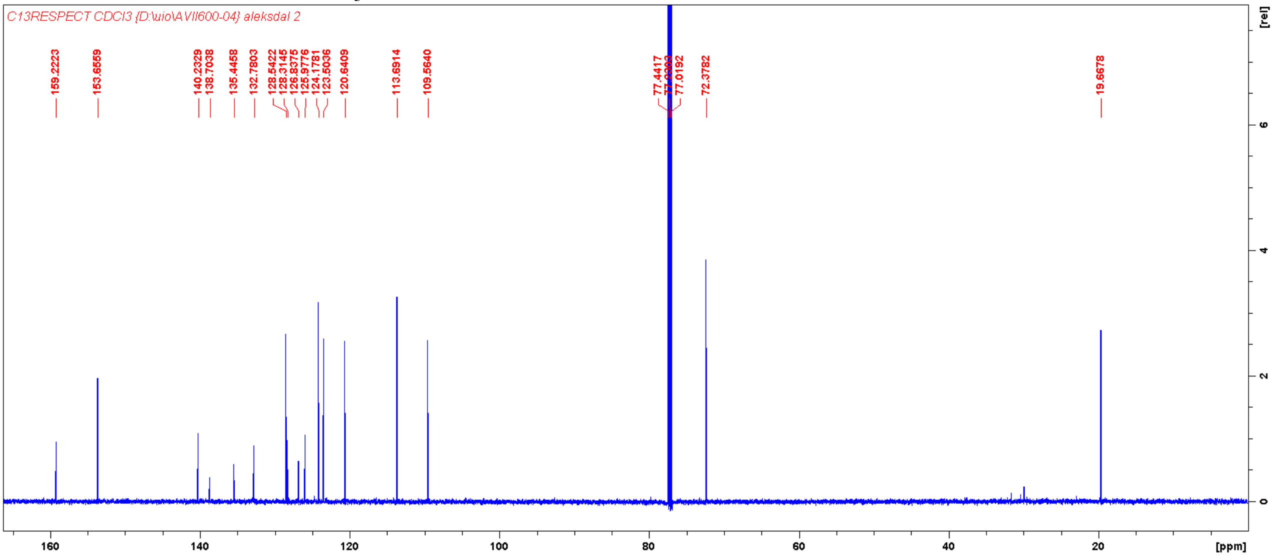

2,4-Dichloro-8-[(2-chloroallyl)oxy]phenanthridine (2g).

^{1}H NMR (600 MHz, $CDCl_3$)

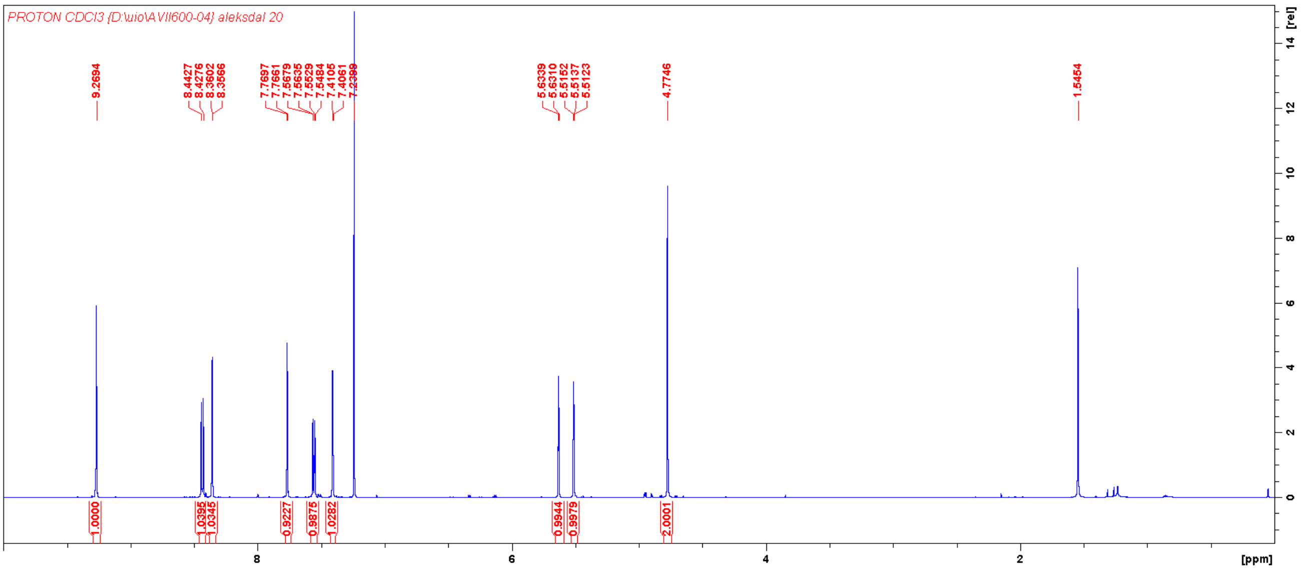

^{13}C NMR (150 MHz, $CDCl_3$)

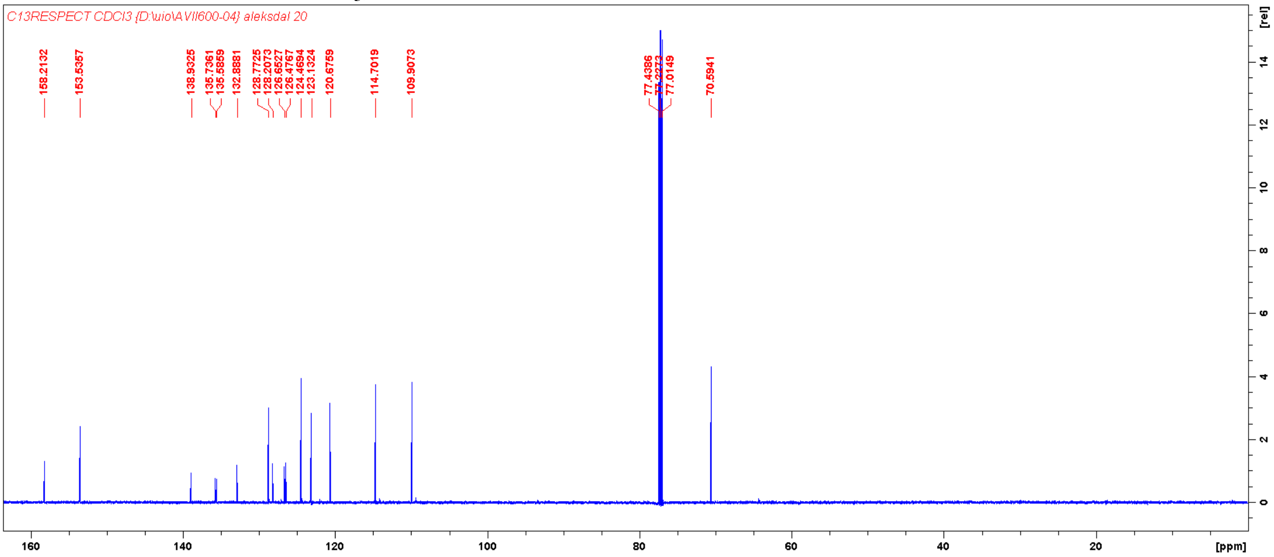

8-(Allyloxy)-2,4-dichloro-7-methylphenanthridine (2h).

^{1}H NMR (600 MHz, $CDCl_3$)

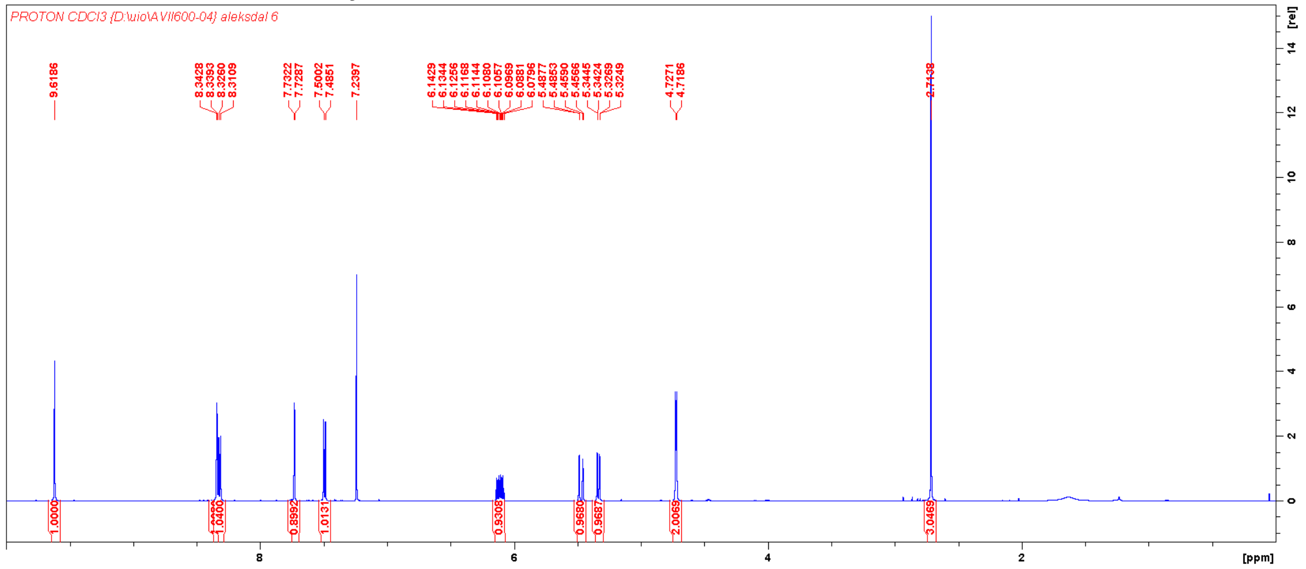

^{13}C NMR (150 MHz, $CDCl_3$)

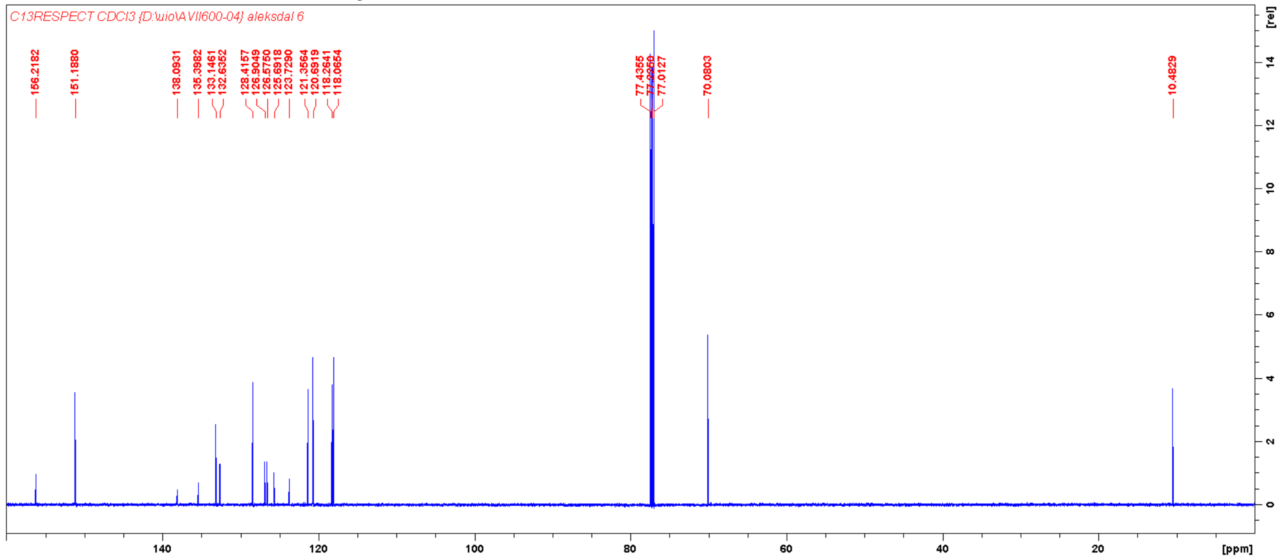

7-Allyl-2,4-dichlorophenanthridin-8-ol (3a).

^{1}H NMR (600 MHz, acetone-d_6)

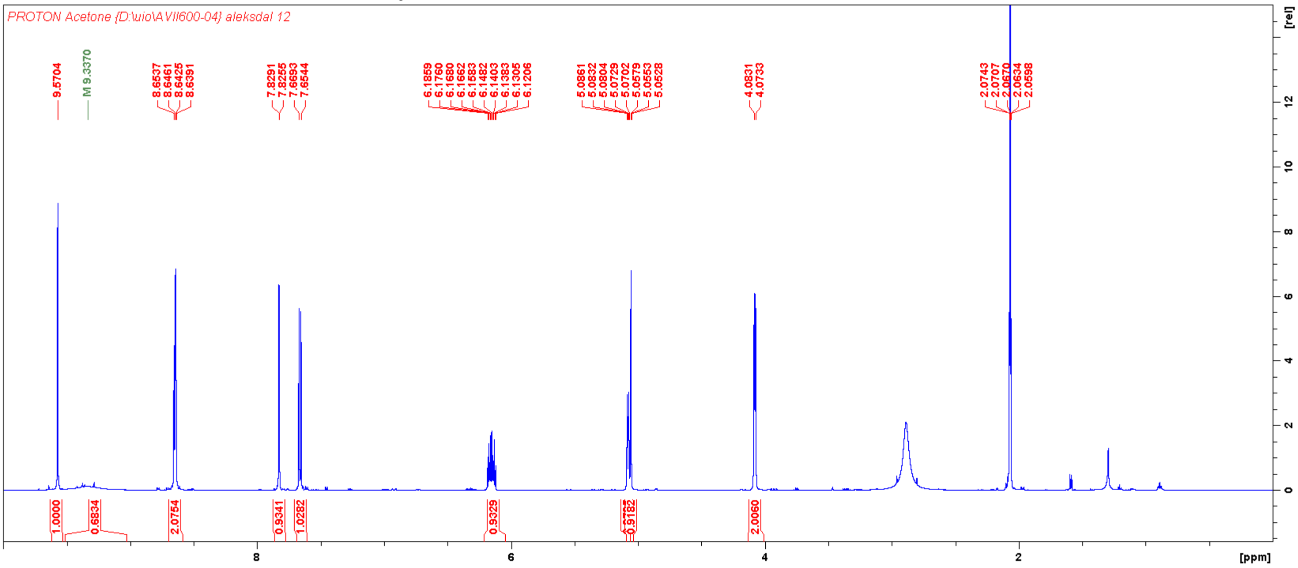

^{13}C NMR (150 MHz, acetone-d_6)

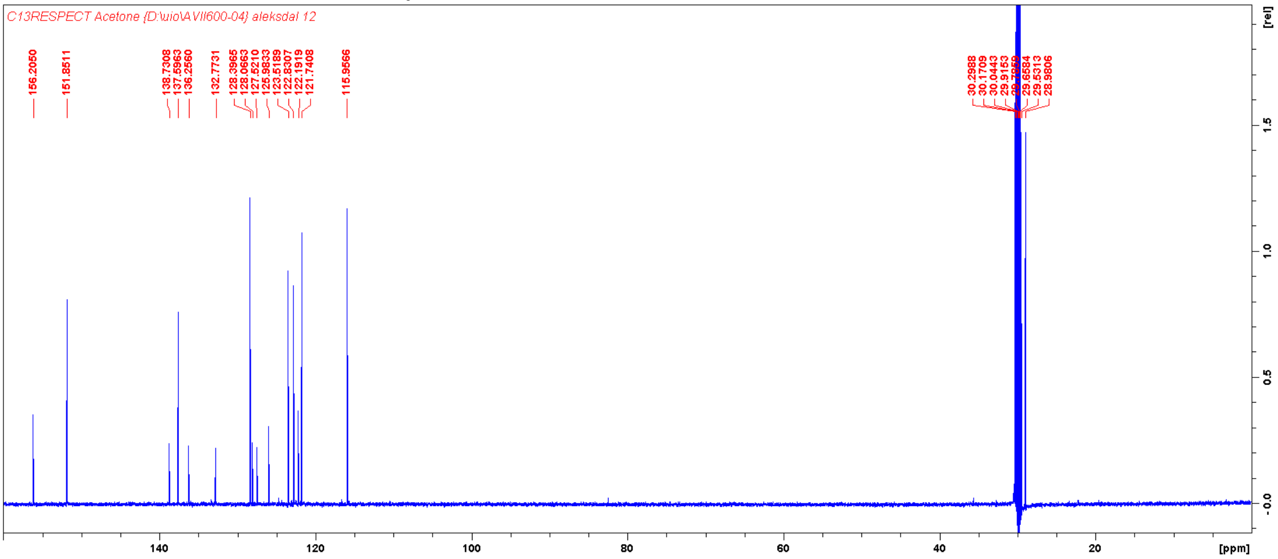

7-Allyl-4-chlorophenanthridin-8-ol (3b).

^{1}H NMR (400 MHz, acetone-d_6)

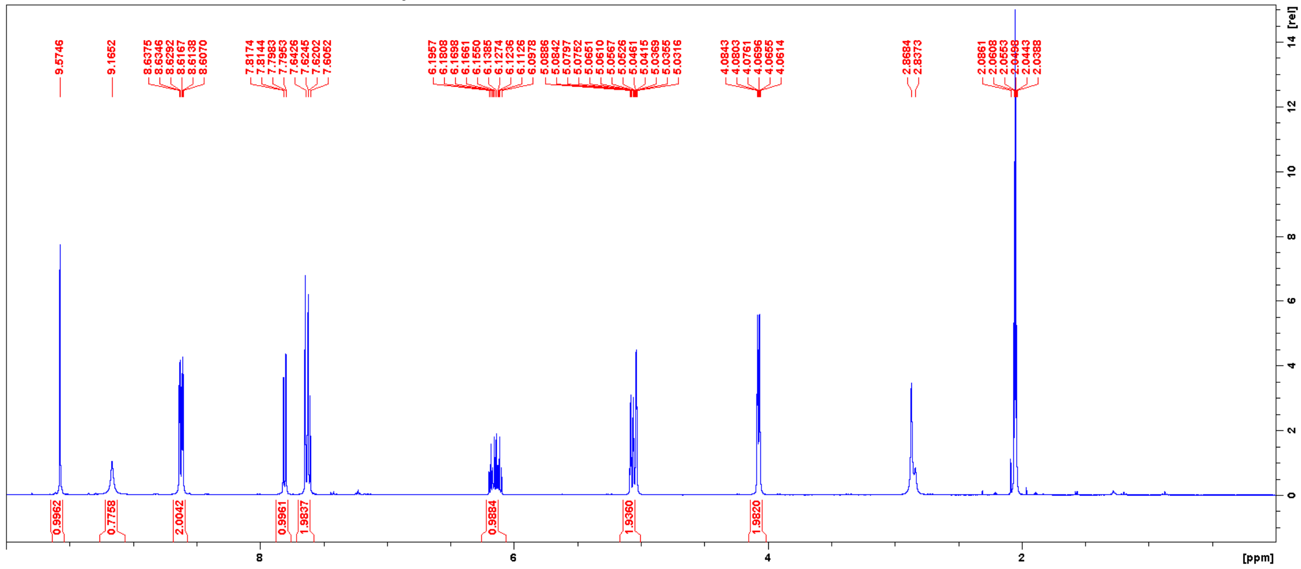

^{13}C NMR (100 MHz, acetone-d_6)

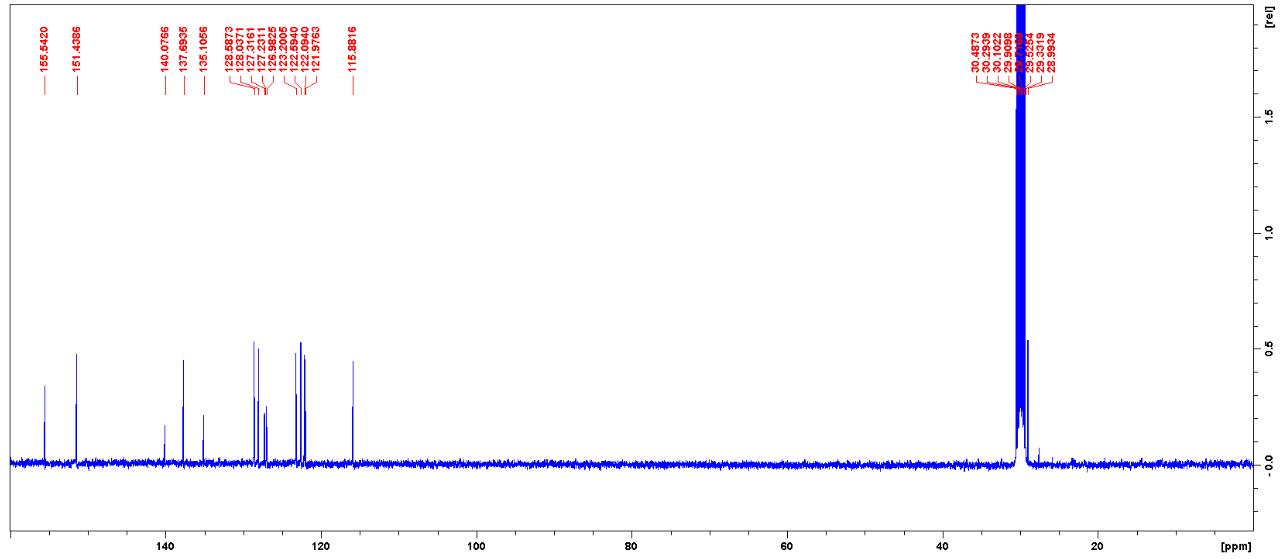

7-Allyl-4-chloro-2-nitrophenanthridin-8-ol (3c).

^{1}H NMR (400 MHz, acetone-d_6)

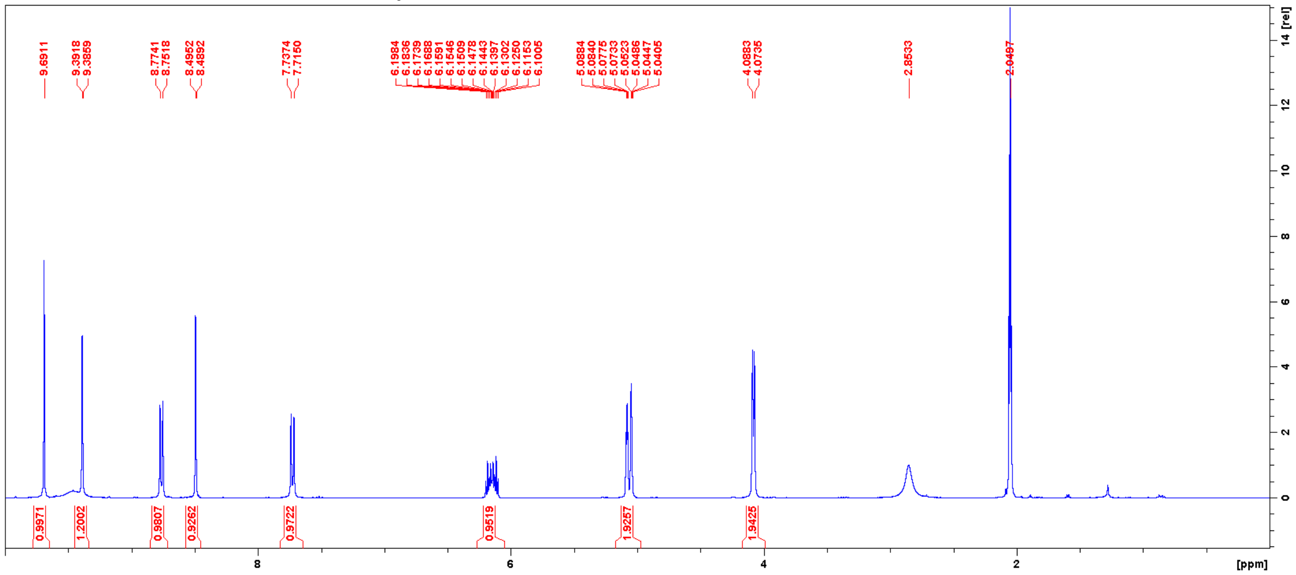

^{13}C NMR (100 MHz, acetone-d_6)

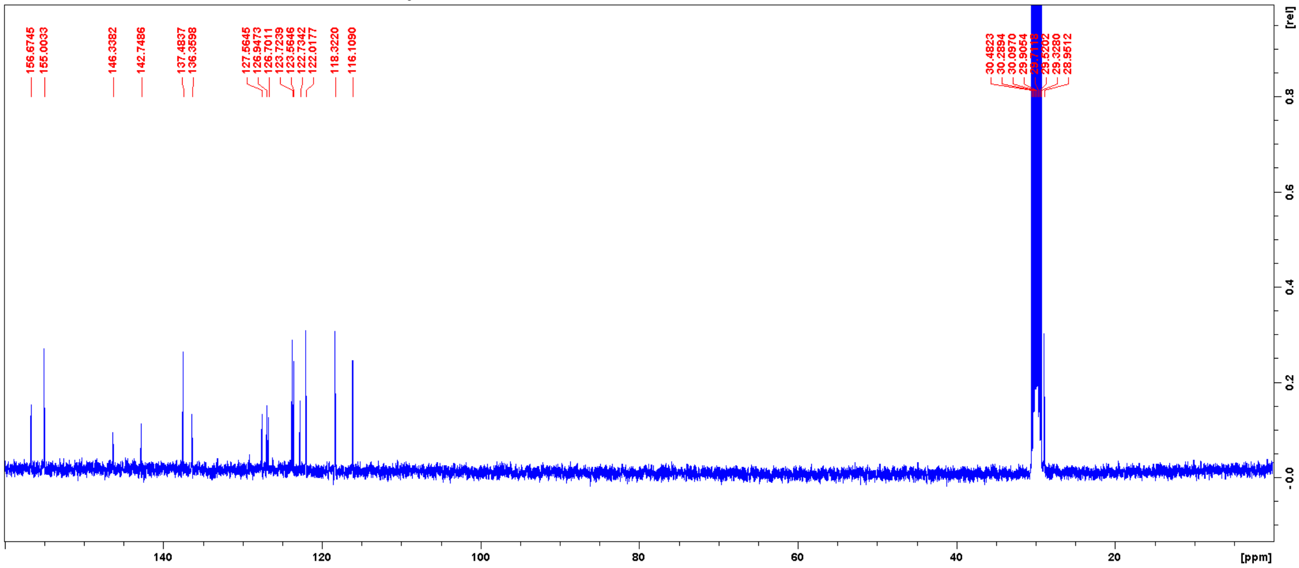

7-(But-3-en-2-yl)-2,4-dichlorophenanthridin-8-ol (3d).

^{1}H NMR (400 MHz, acetone-d_6)

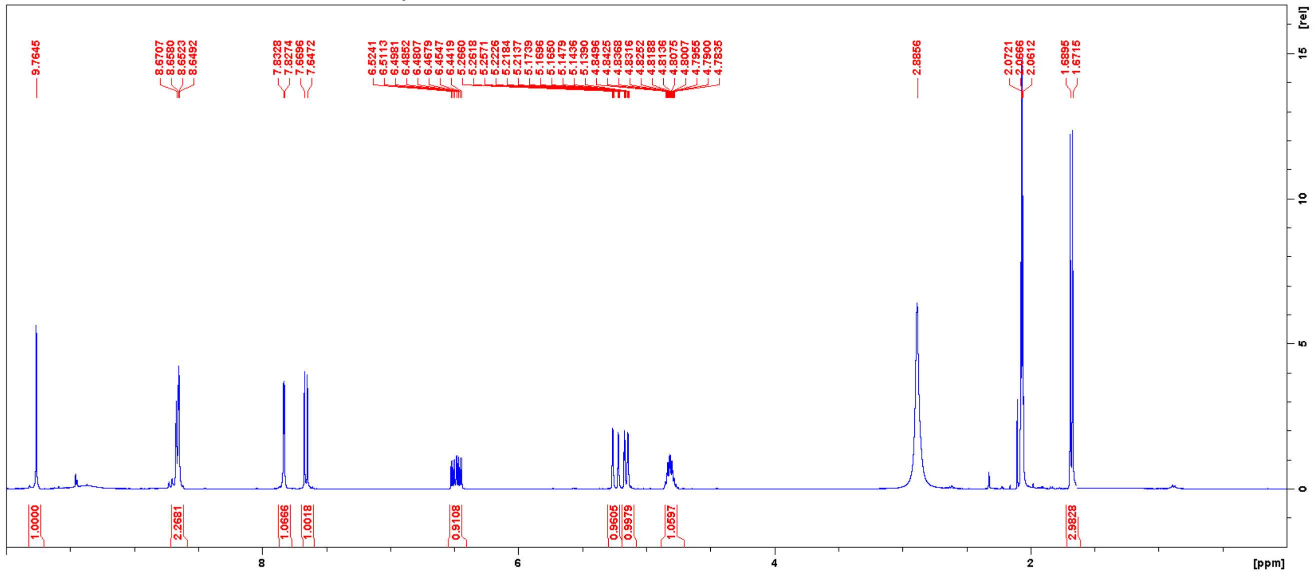

^{13}C NMR (100 MHz, acetone-d_6)

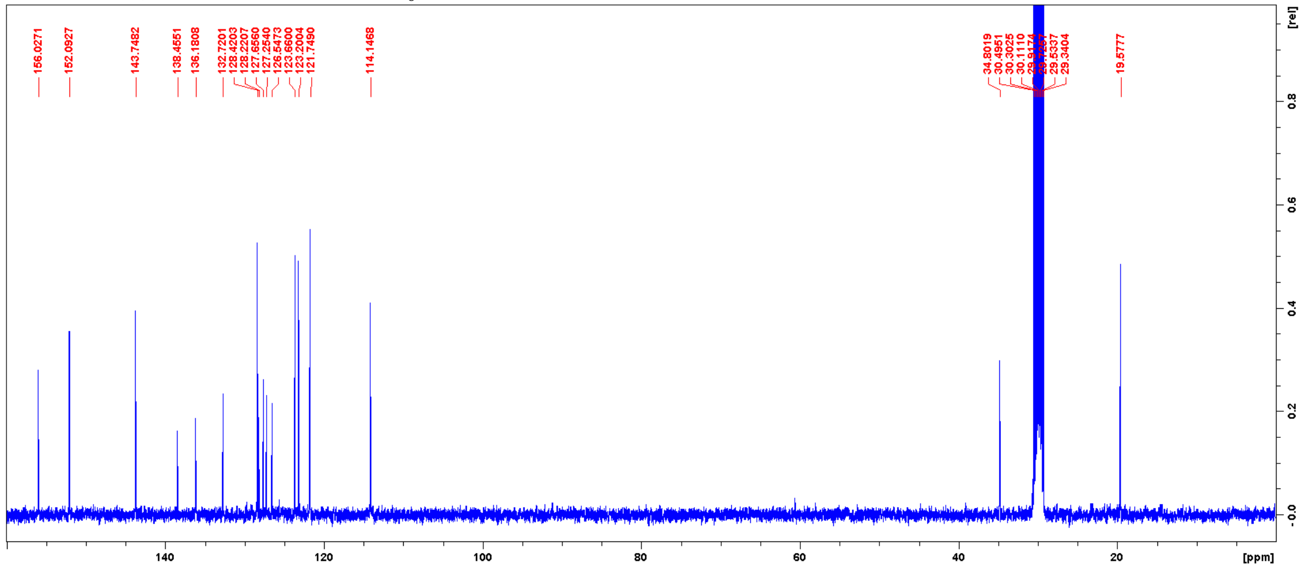

2,4-Dichloro-7-(2-methylallyl)phenanthridin-8-ol (3f).

^{1}H NMR (400 MHz, DMSO-d_6)

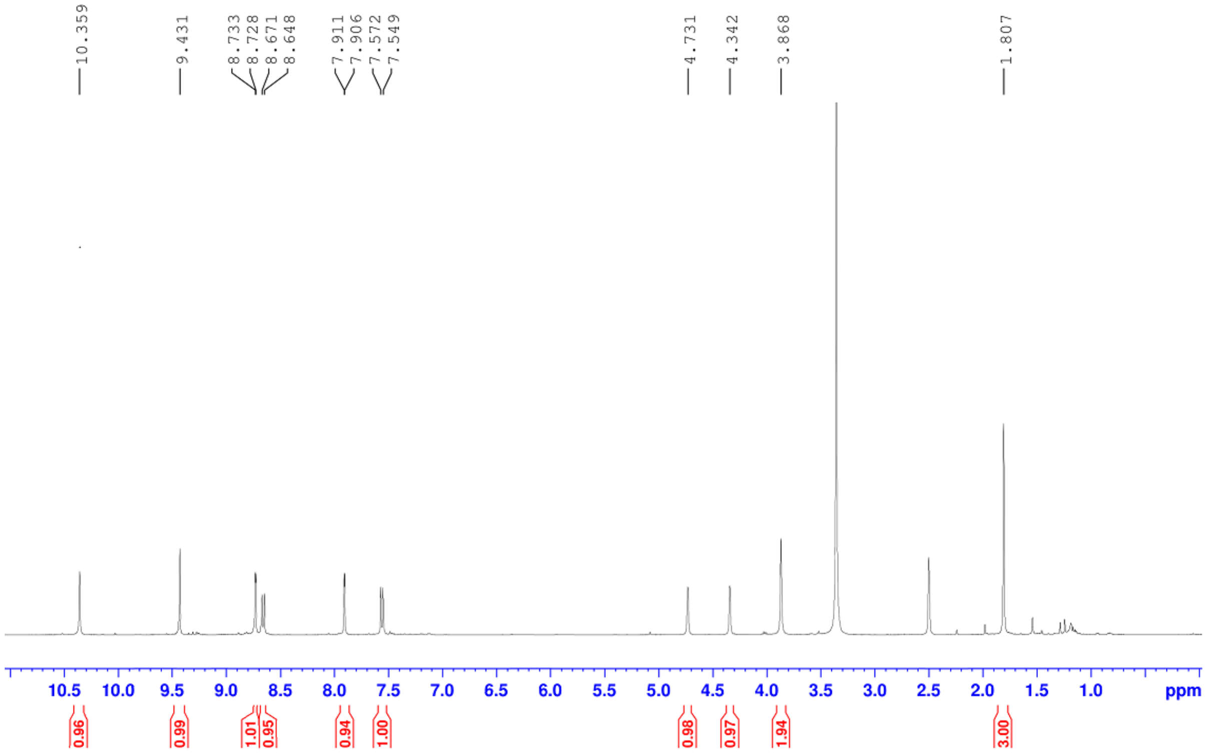

^{13}C NMR (100 MHz, DMSO-d_6)

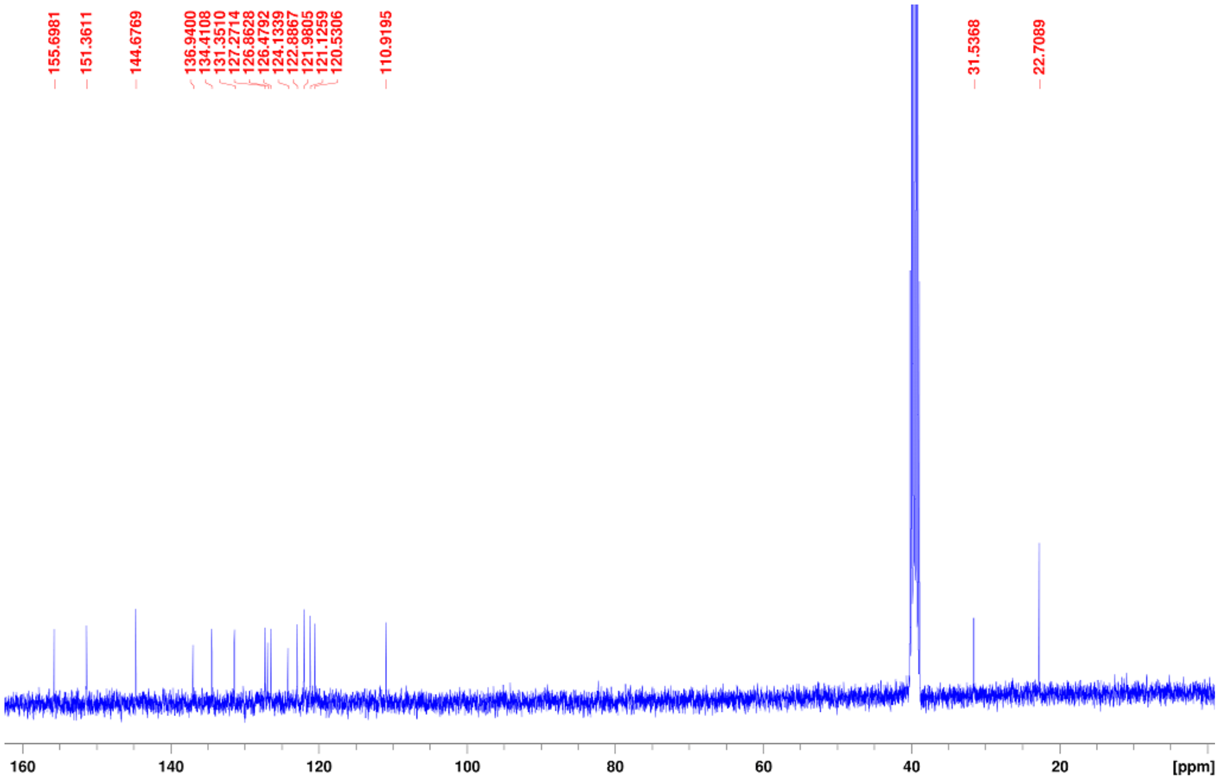

2,4-Dichloro-7-(2-chloroallyl)phenanthridin-8-ol (3g).

^{1}H NMR (400 MHz, acetone-d_6)

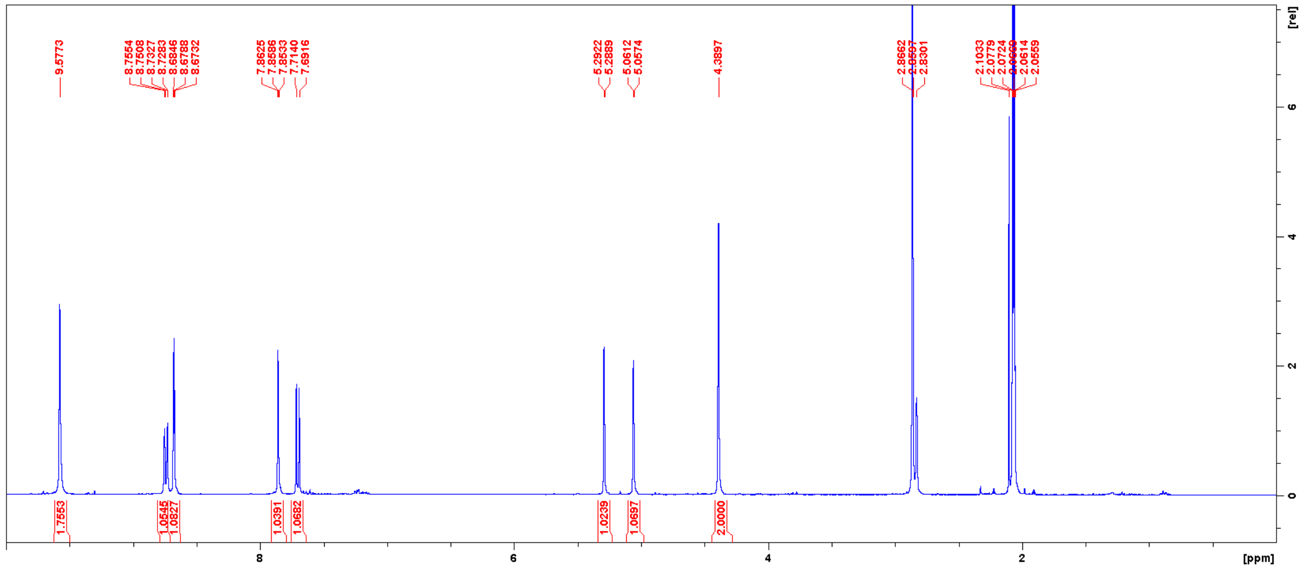

^{13}C NMR (100 MHz, acetone-d_6)

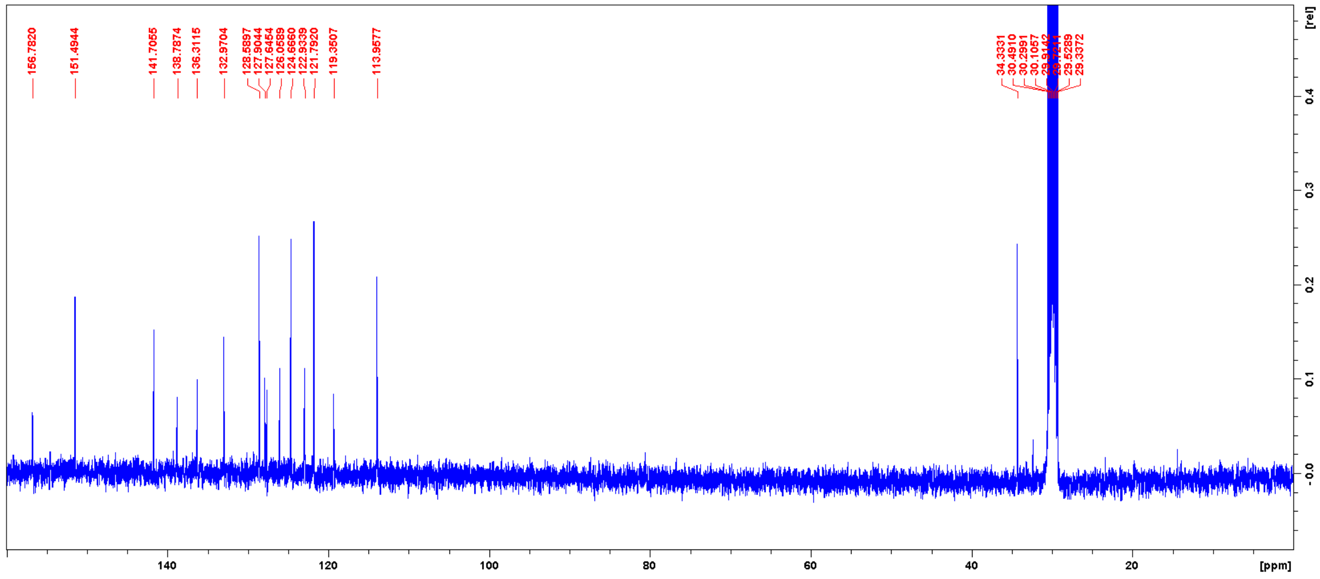

6,8-Dichloro-2,2-dimethyl-2,3-dihydrofuro[3,2-*i*]phenanthridine (4).

^{1}H NMR (400 MHz, DMSO-d_6)

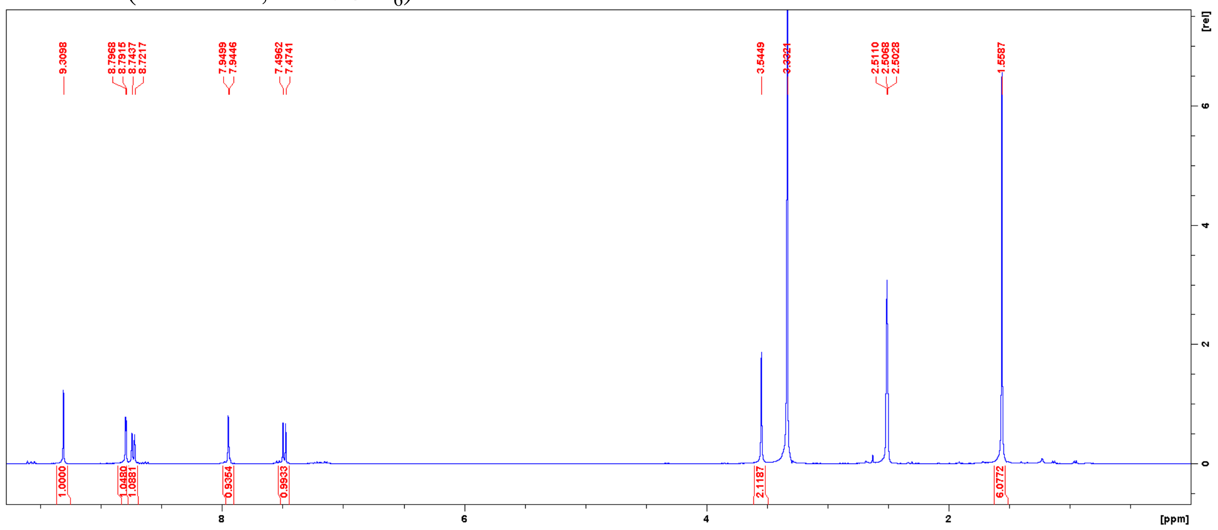

^{13}C NMR (100 MHz, DMSO-d_6)

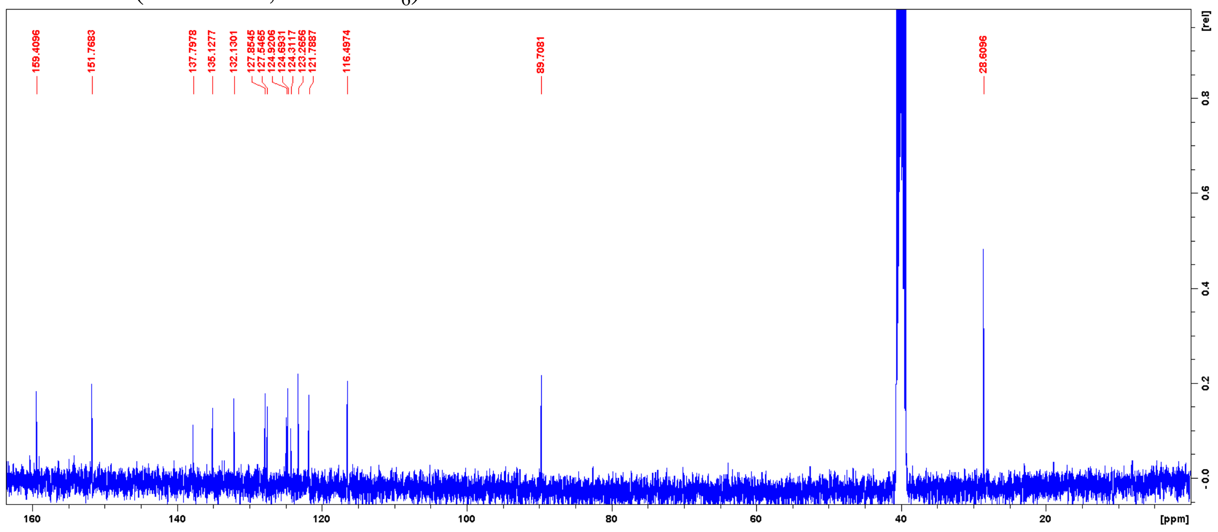

9-Allyl-2,4-dichloro-7-methylphenanthridin-8-ol (5).

^{1}H NMR (400 MHz, acetone-d_6)

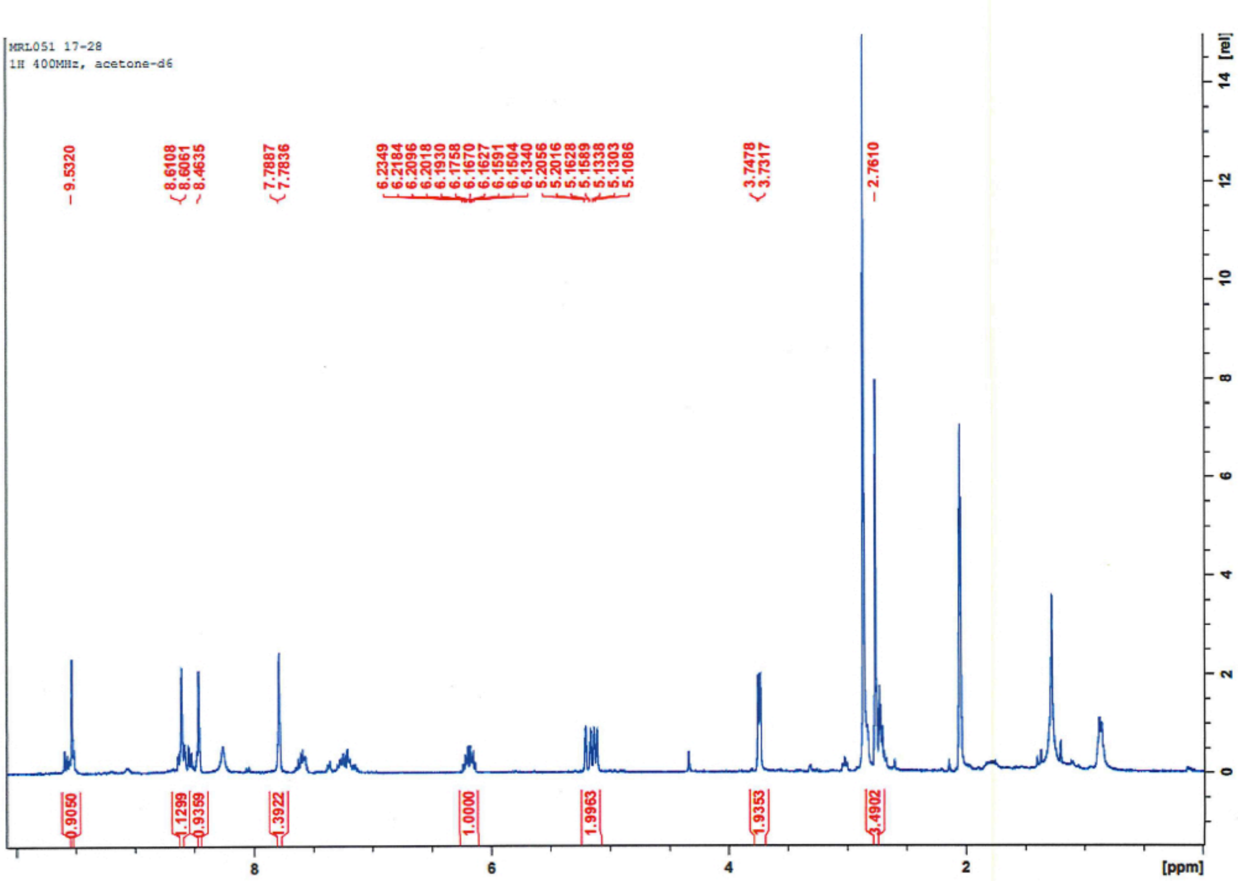

_N_-(But-2-ynyl)-2,4-dichloro-6-(furan-2-yl)aniline (7b).

^{1}H NMR (600 MHz, $CDCl_3$)

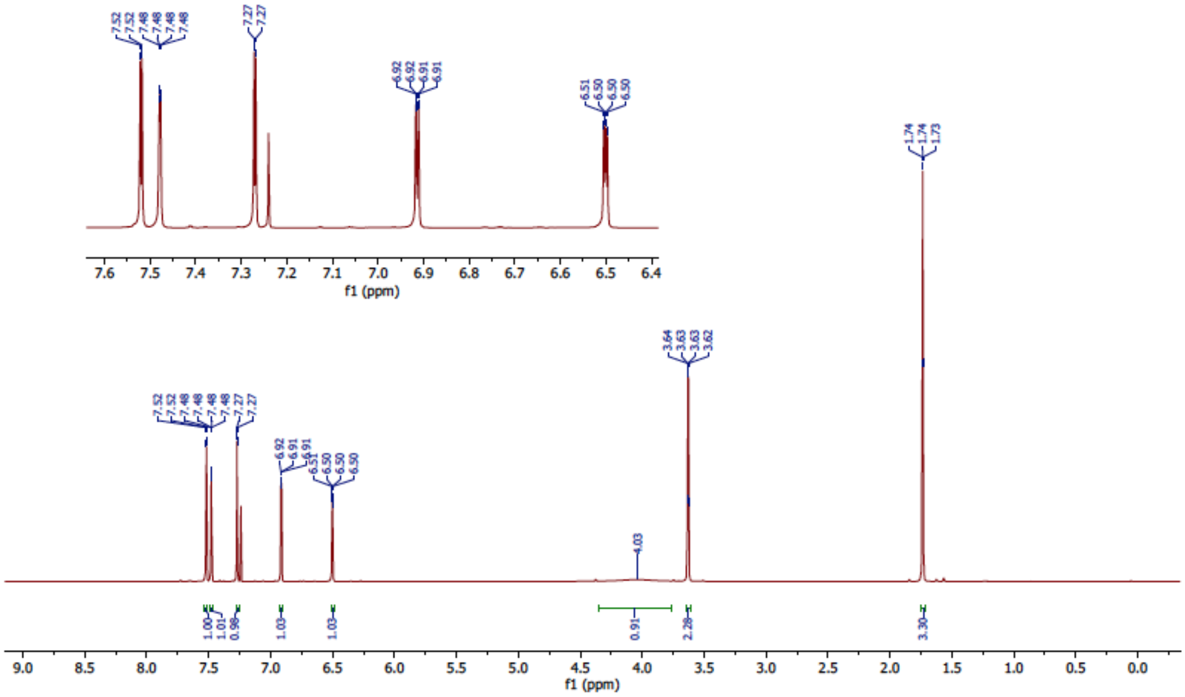

^{13}C NMR (150 MHz, $CDCl_3$)

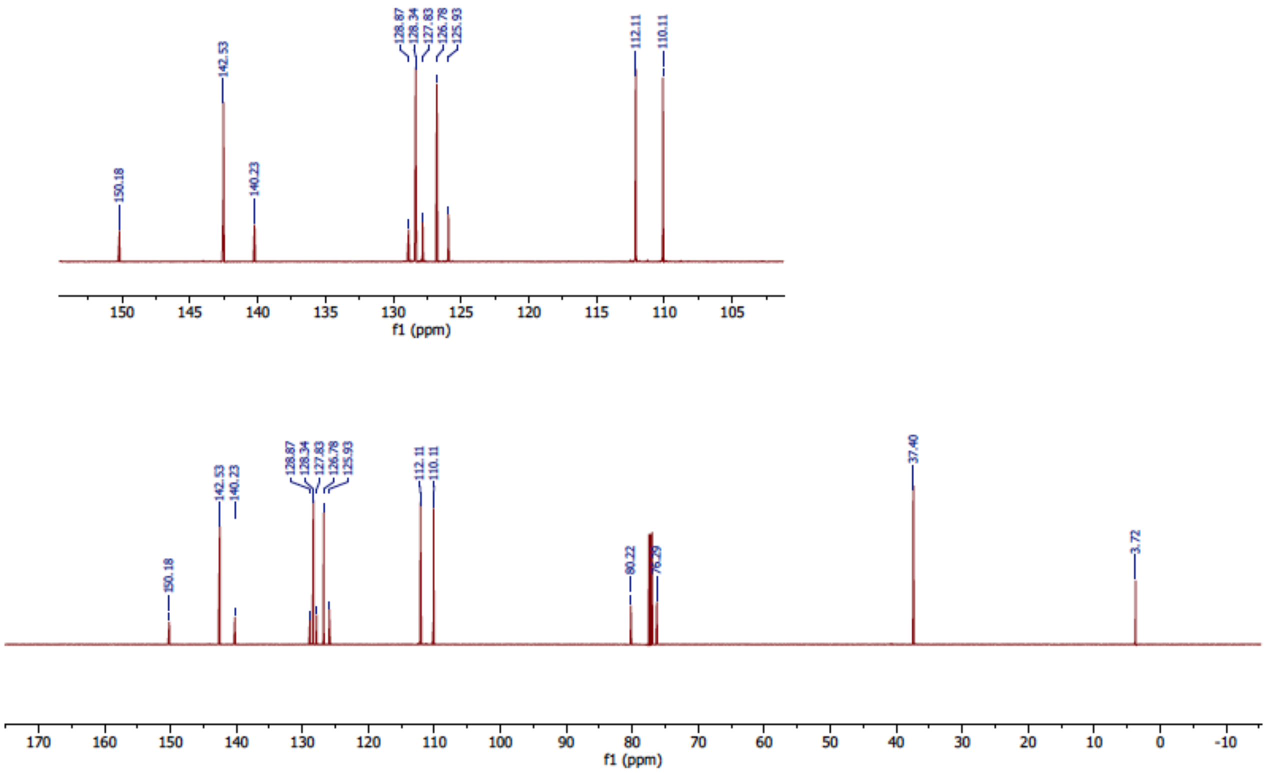

2,4-Dichloro-6-(furan-2-yl)-*N*-methyl-*N*-(prop-2-yn-1-yl)aniline (8a).

^{1}H NMR, 300 MHz, $CDCl_3$

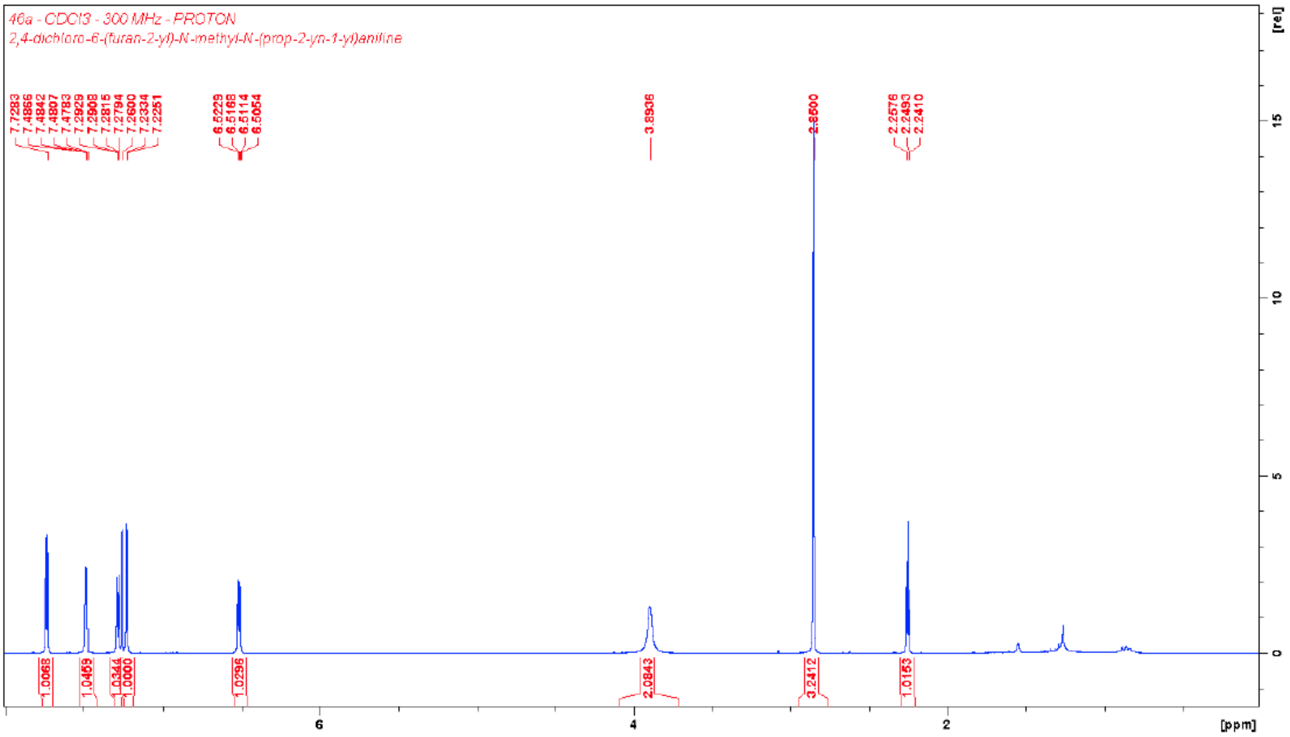

^{13}C NMR, 100 MHz, $CDCl_3$

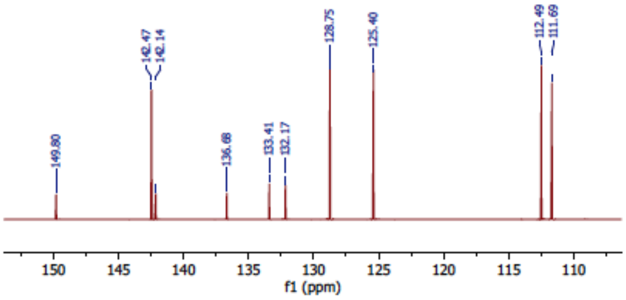

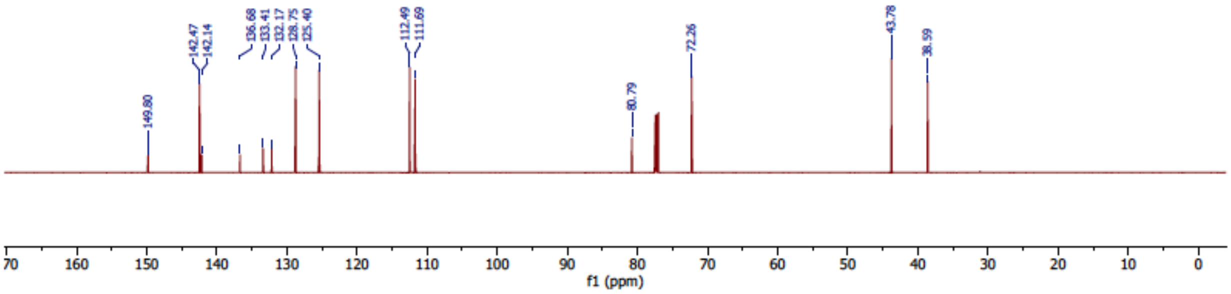

N-(But-2-ynyl)-2,4-dichloro-6-(furan-2-yl)-N-methylaniline (8b).

^{1}H NMR (600 MHz, $CDCl_3$)

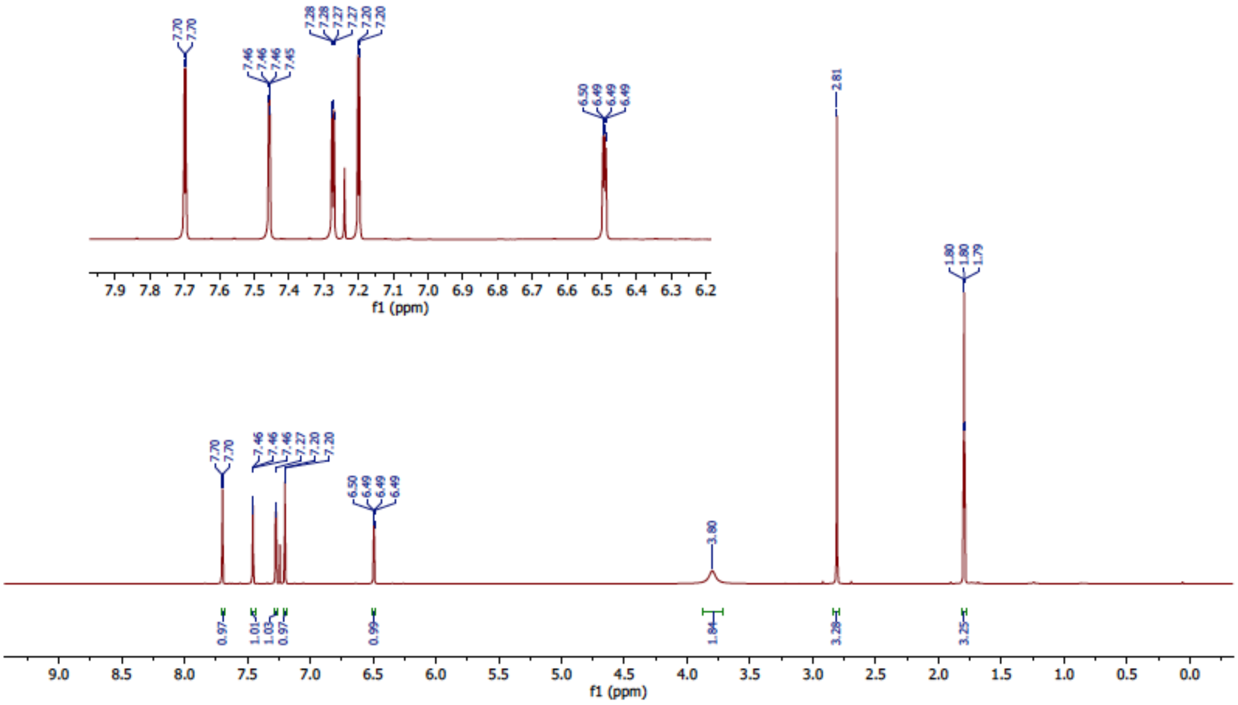

^{13}C NMR (150 MHz, $CDCl_3$)

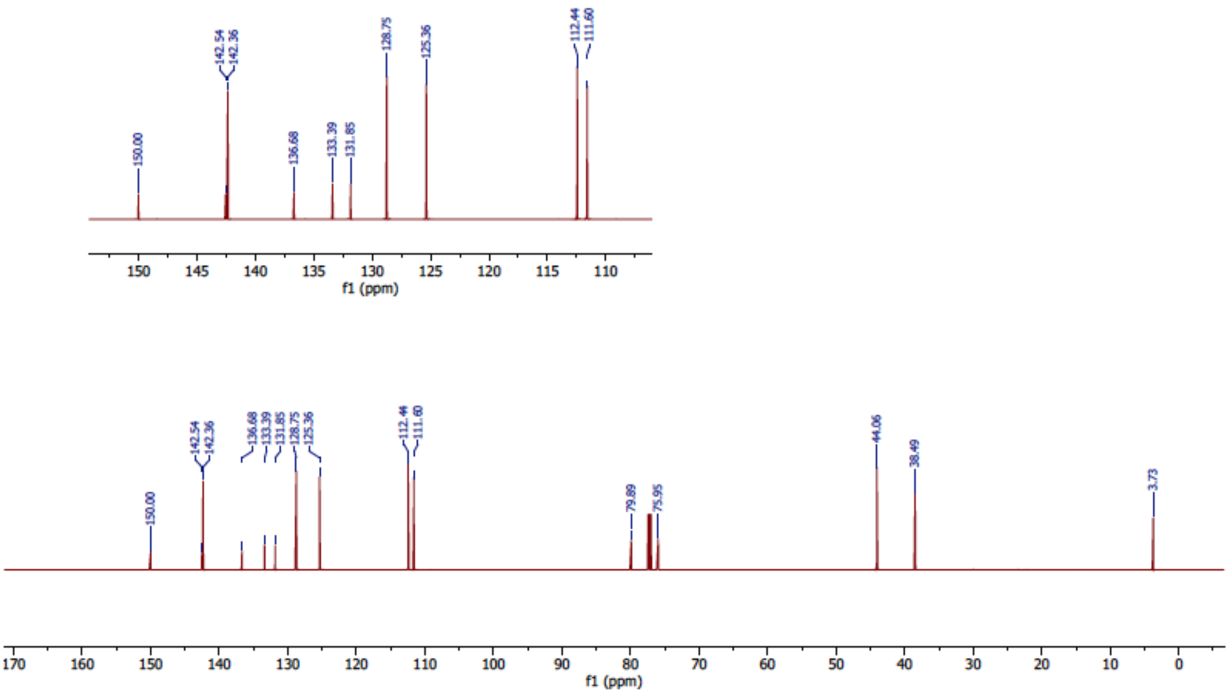

2,4-Dichloro-5-methyl-5,6-dihydrophenanthridin-8-ol (9a).

^{1}H NMR (400 MHz, DMSO-d_6)

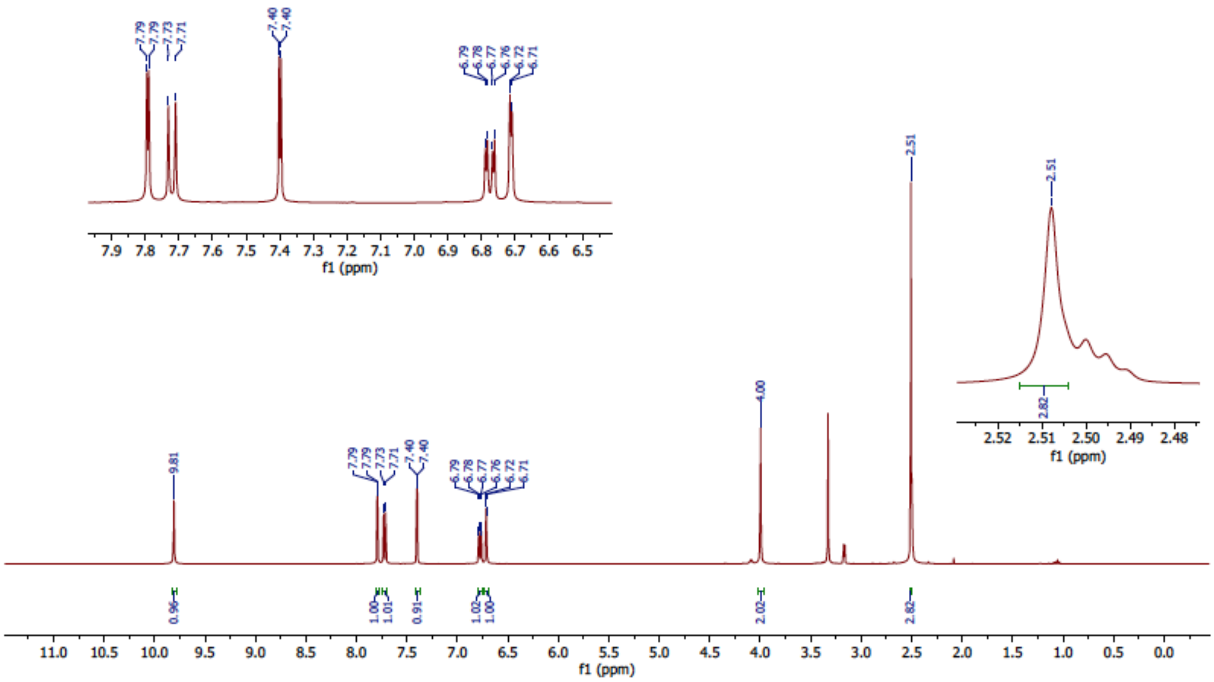

^{13}C NMR (100 MHz, DMSO-d_6) δ

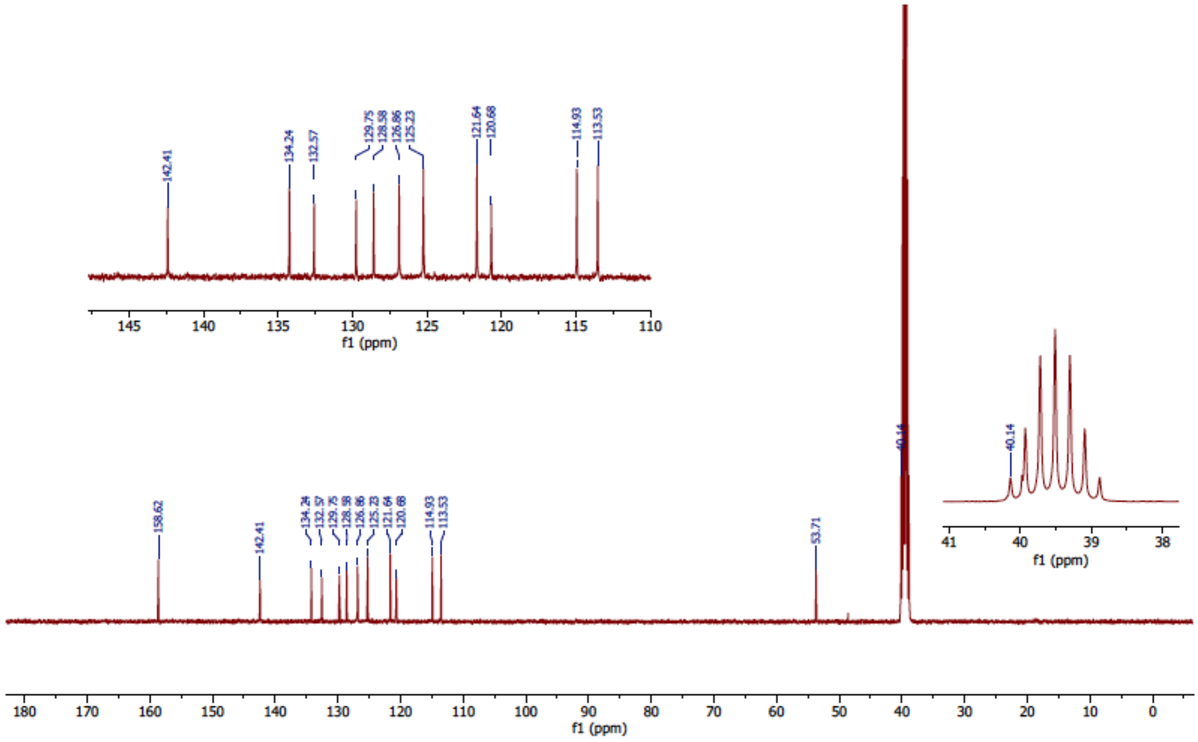

2,4-Dichloro-5,7-dimethyl-5,6-dihydrophenanthridin-8-ol (9b).

^{1}H NMR (400 MHz, DMSO-d_6)

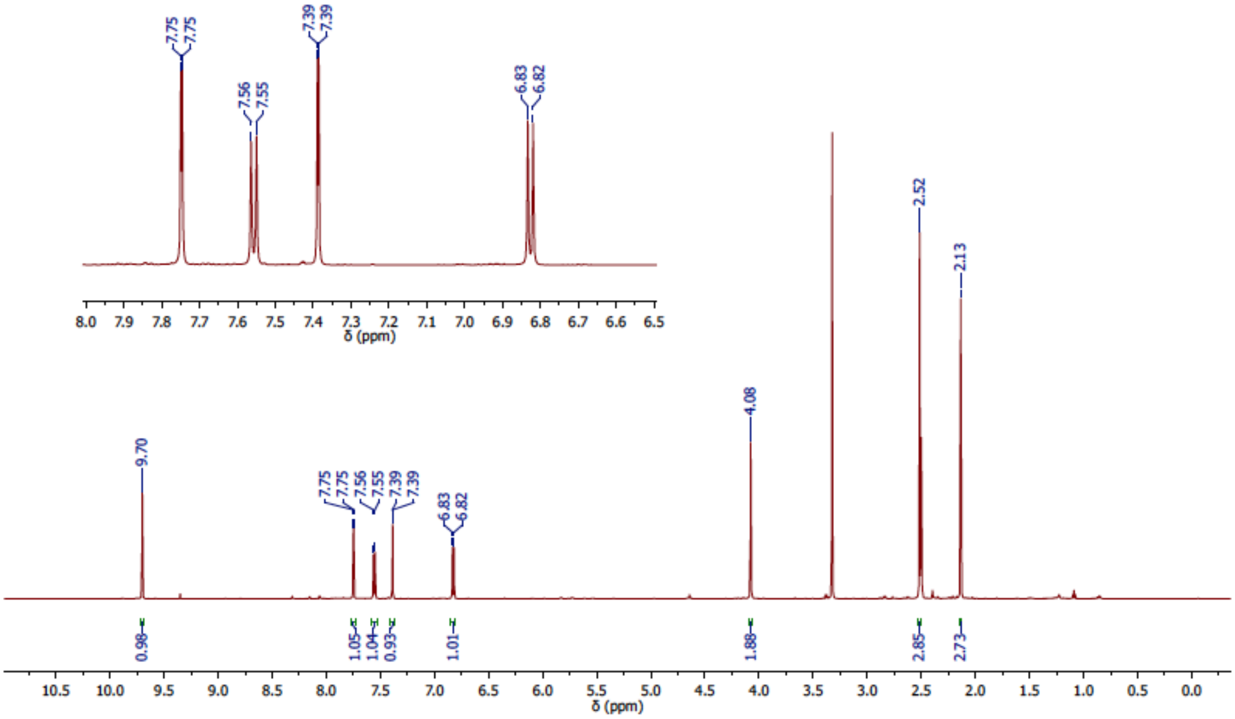

^{13}C NMR (100 MHz, DMSO-d_6)

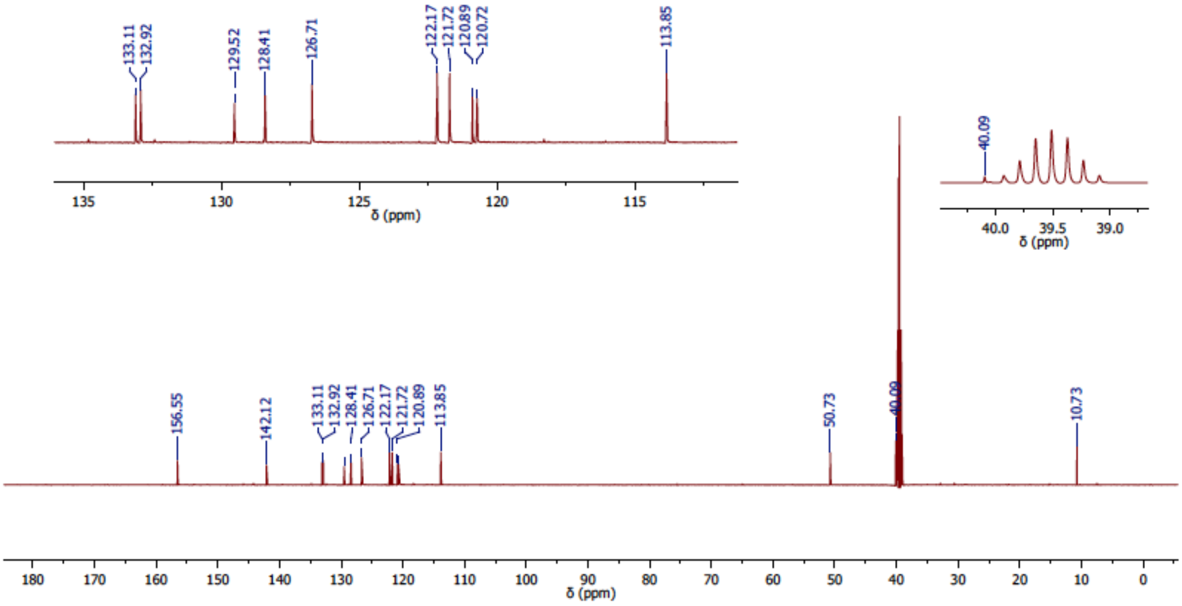

8-(Allyloxy)-2,4-dichloro-5-methyl-5,6-dihydrophenanthridine (10a).

^{1}H NMR (400 MHz, $CDCl_3$)

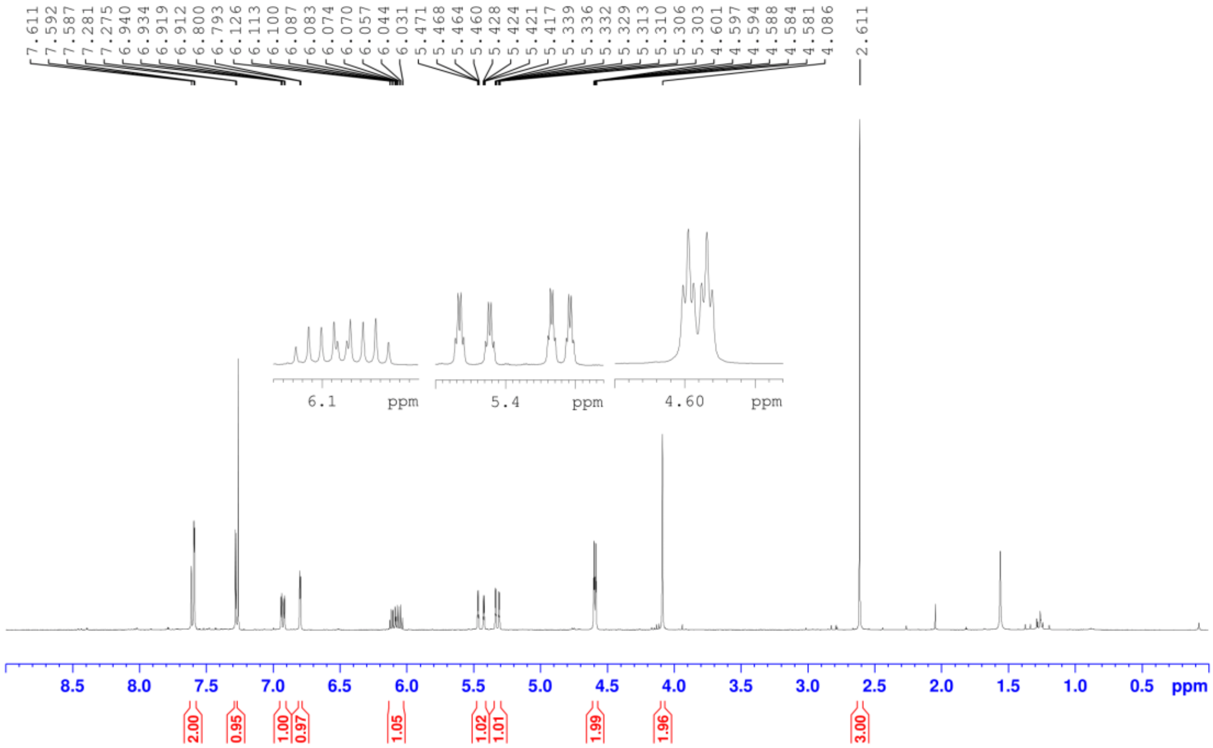

^{13}C NMR (100 MHz, $CDCl_3$)

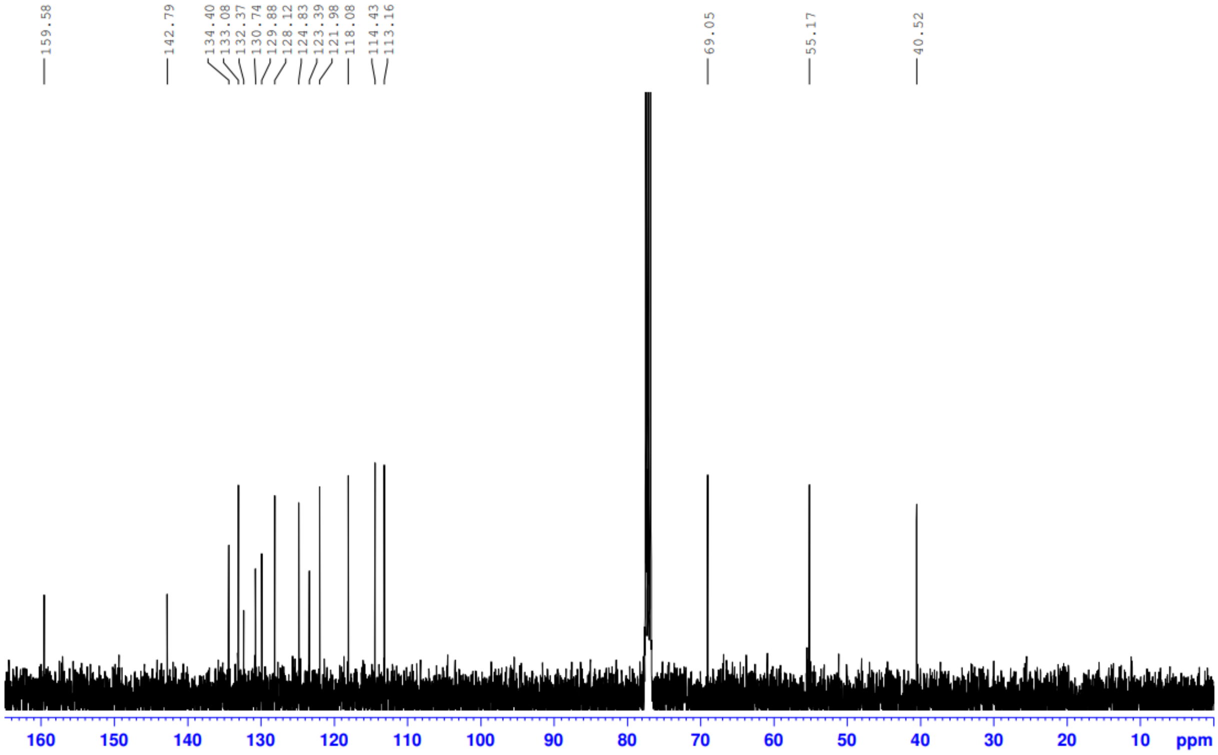

2,4-Dichloro-5-methyl-8-[(2-methylallyl)oxy]- 5,6-dihydrophenanthridine (10b).

^{1}H NMR (400 MHz, $CDCl_3$)

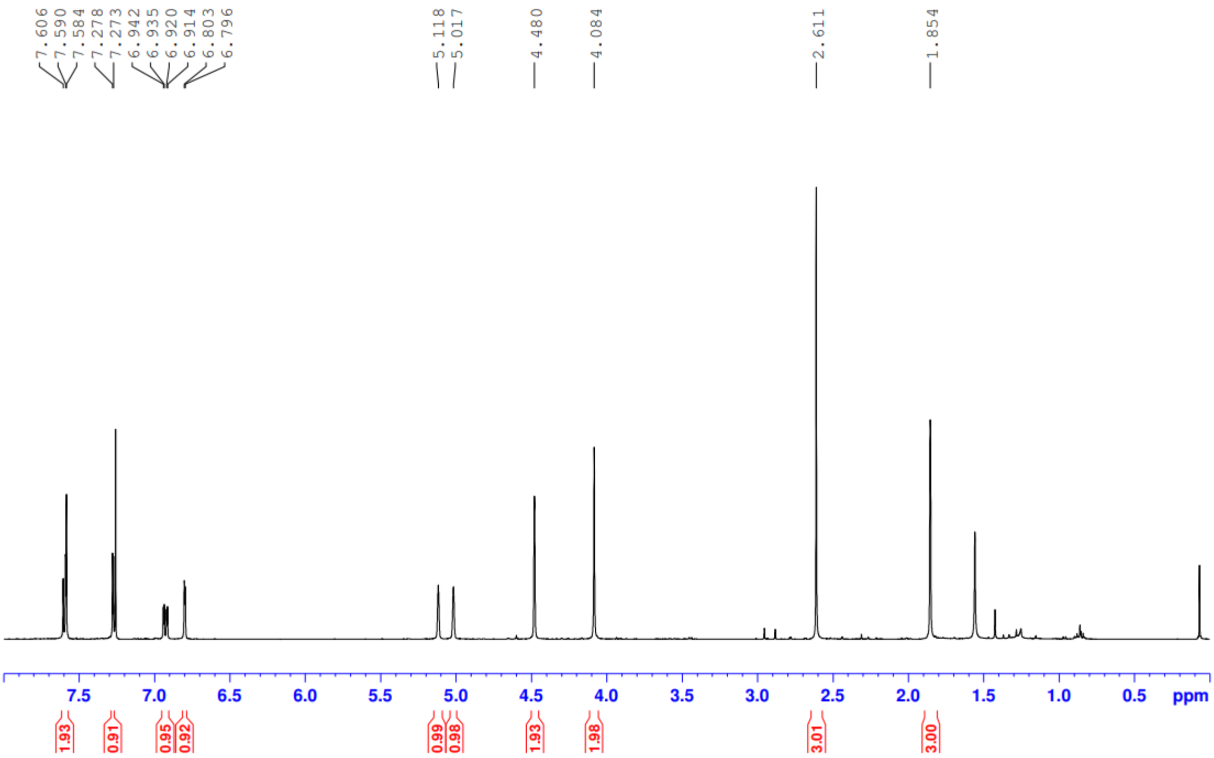

^{13}C NMR (100 MHz, $CDCl_3$)

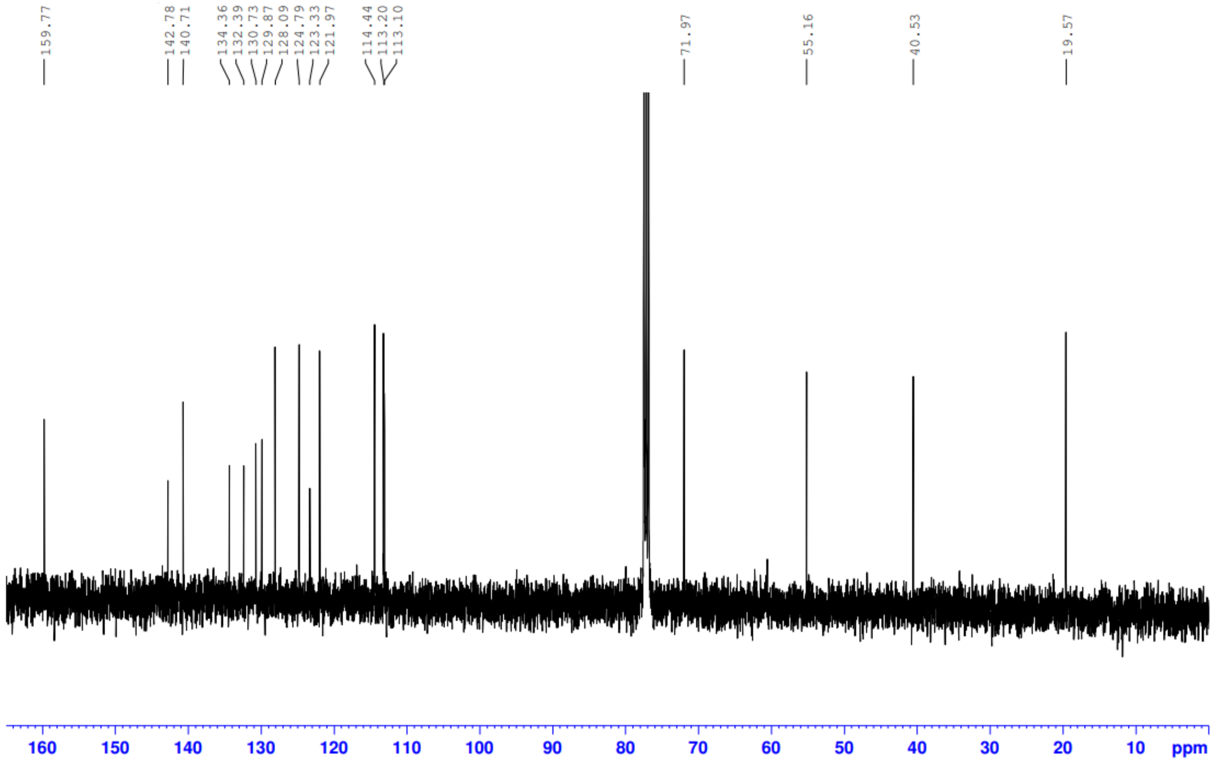

8-(Allyloxy)-2,4-dichloro-5,7-dimethyl-5,6-dihydrophenanthridine (10c).

^{1}H NMR (400 MHz, $CDCl_3$)

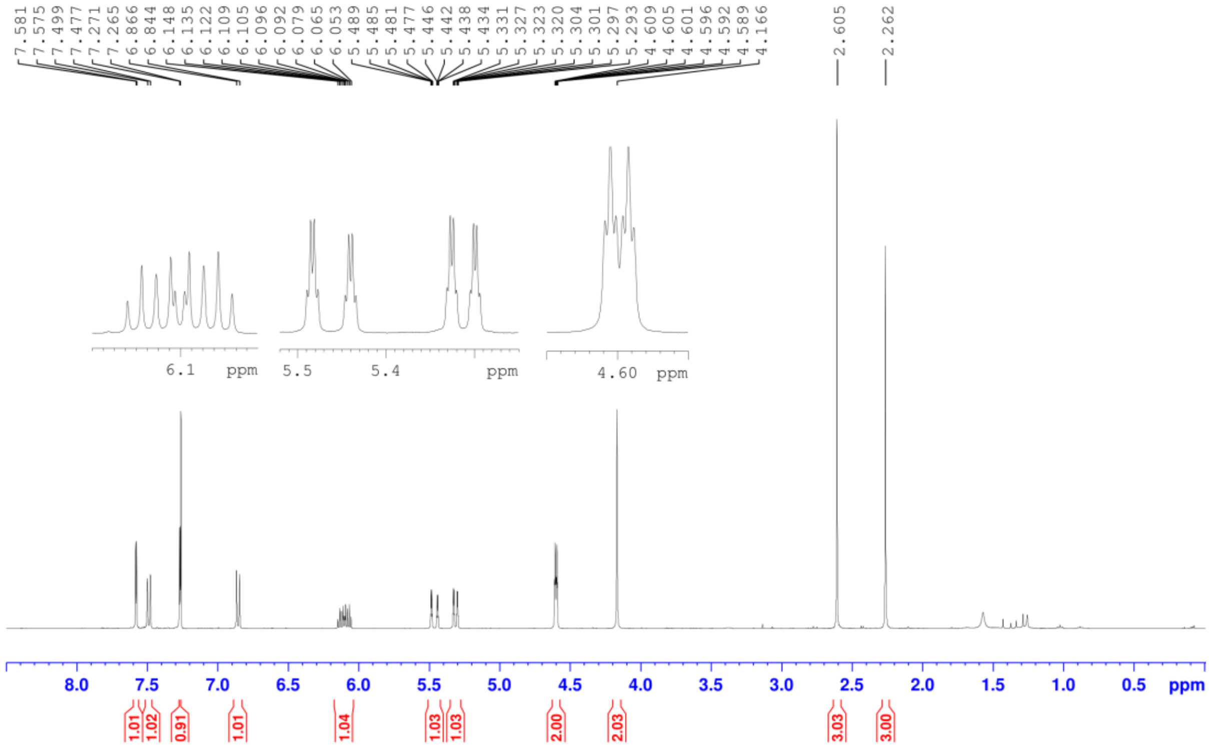

^{13}C NMR (100 MHz, $CDCl_3$)

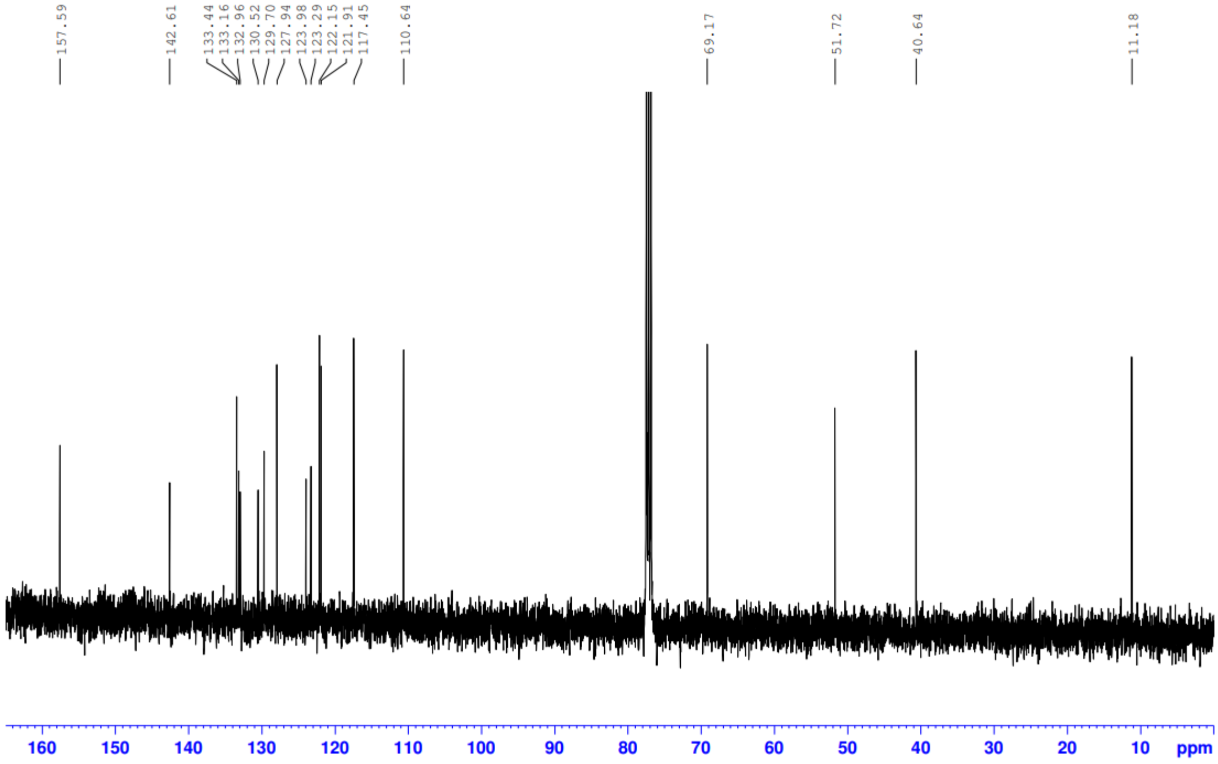

2,4-Dichloro-5,7-dimethyl-8-[(2-methylallyl)oxy]- 5,6-dihydrophenanthridine (10d).

^{1}H NMR (400 MHz, $CDCl_3$)

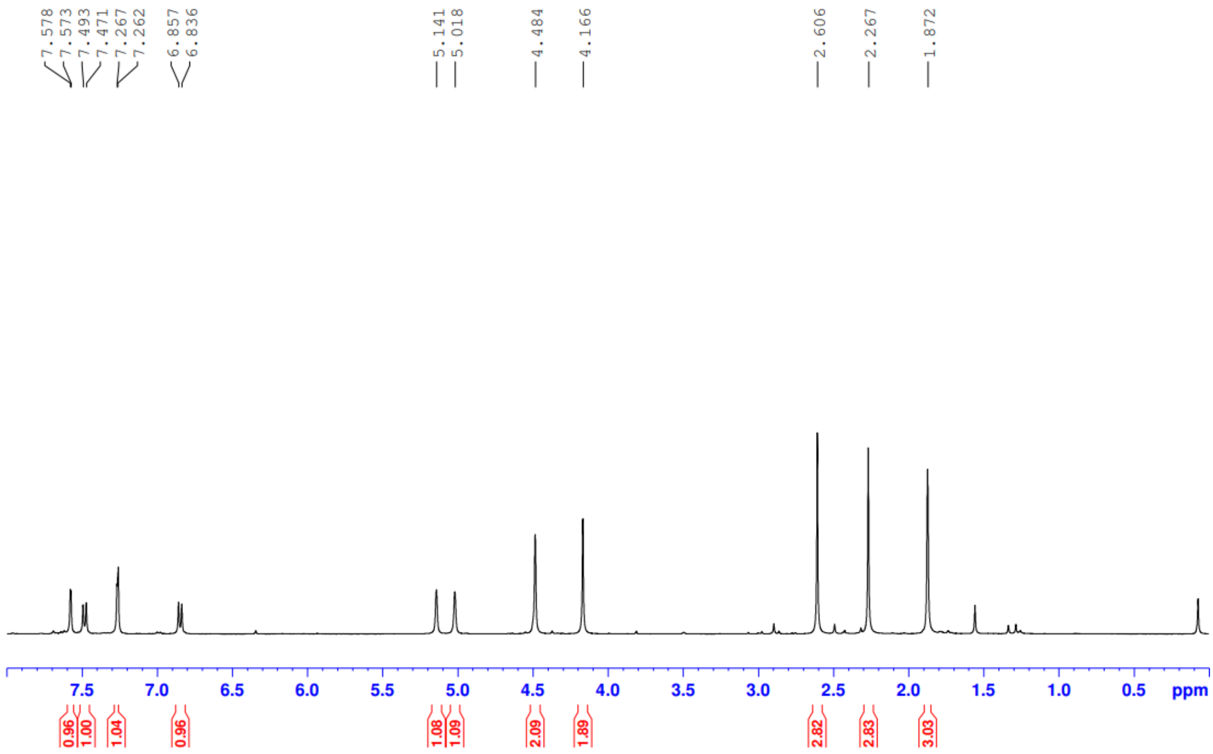

^{13}C NMR (100 MHz, $CDCl_3$)

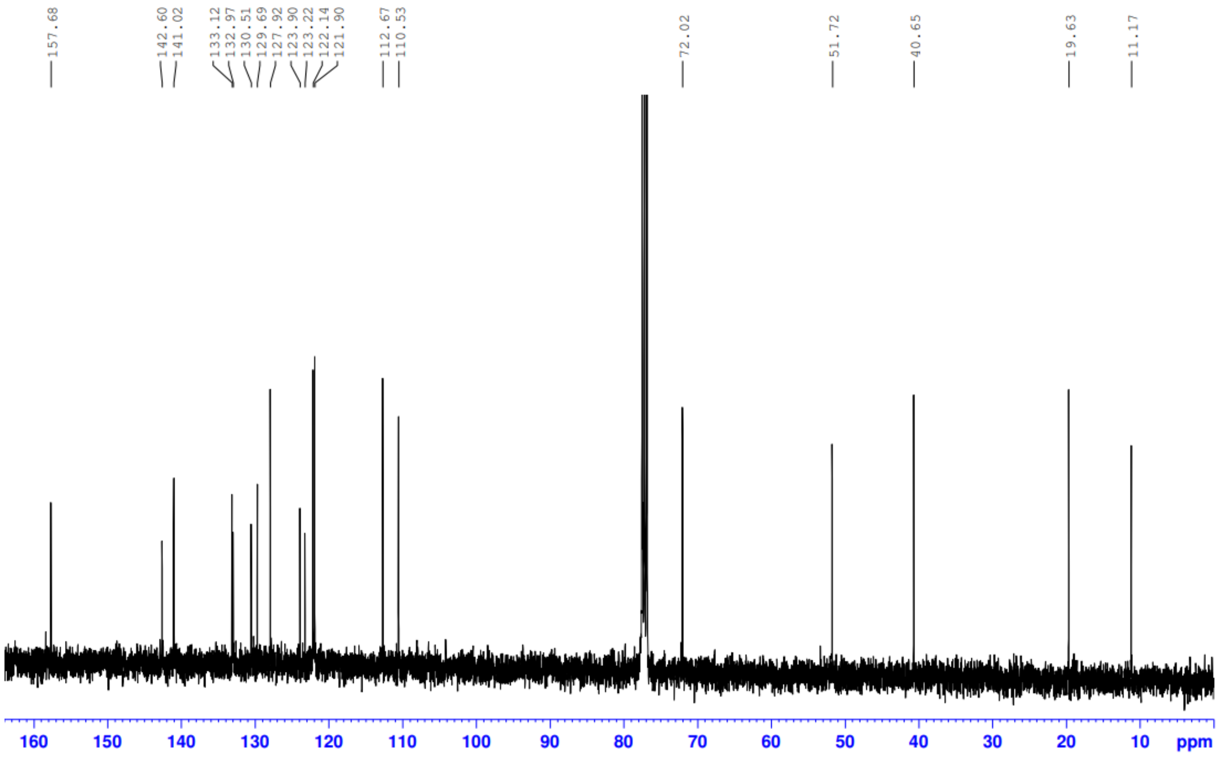

7-Allyl-2,4-dichloro-5-methyl-5,6-dihydrophenanthridin-8-ol (11a).

^{1}H NMR (400 MHz, acetone-d_6)

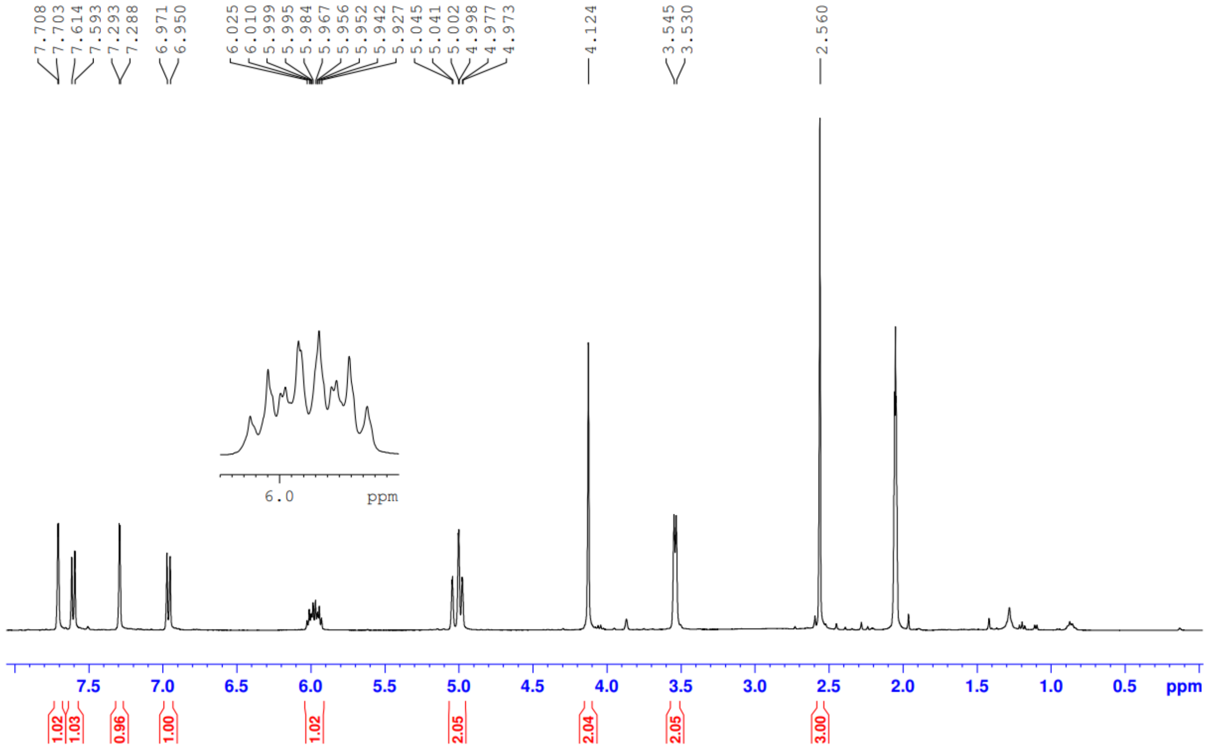

^{13}C NMR (100 MHz, acetone-d_6)

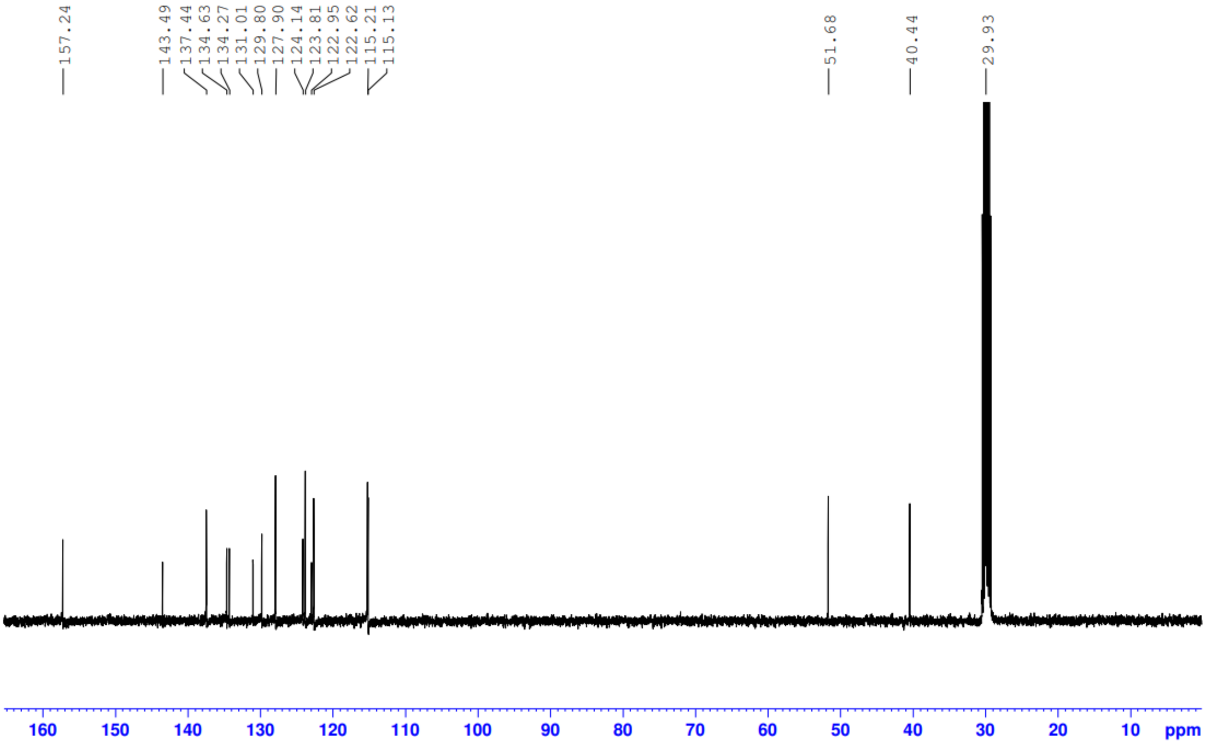

2,4-Dichloro-5-methyl-7-(2-methylallyl)-5,6-dihydrophenanthridin-8-ol (11b).

^{1}H NMR (400 MHz, $CDCl_3$)

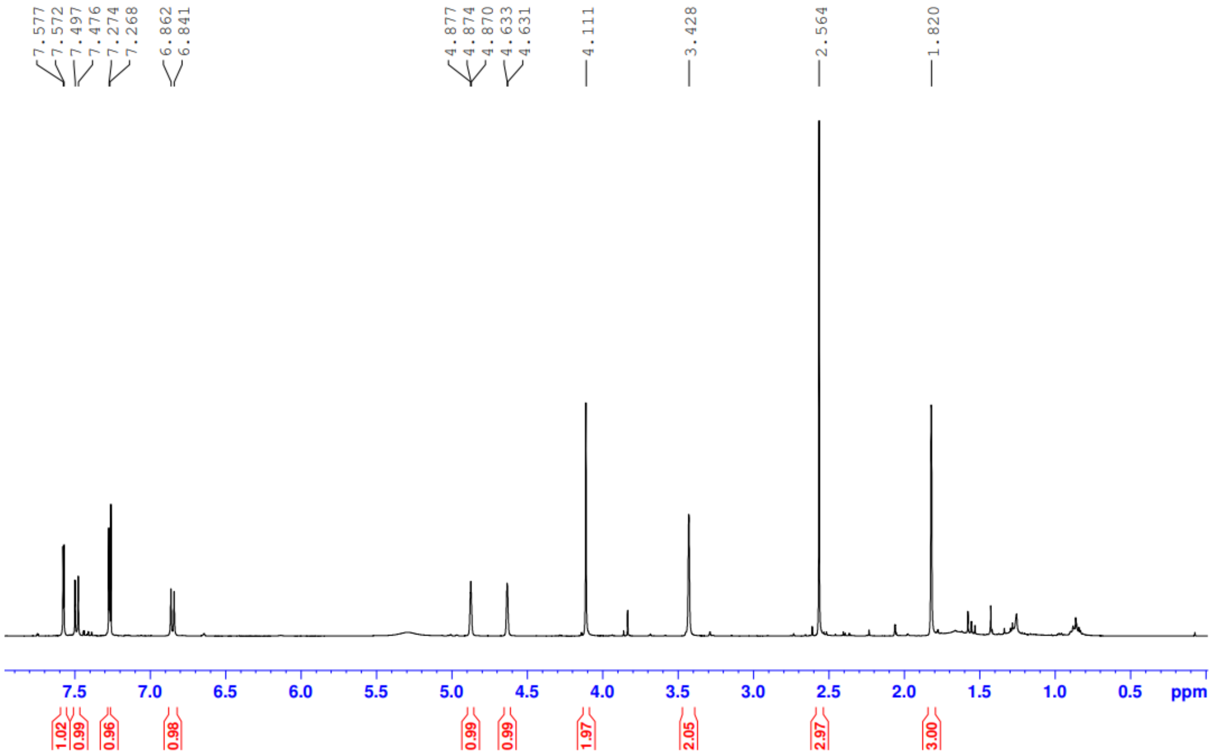

^{13}C NMR (100 MHz, $CDCl_3$)

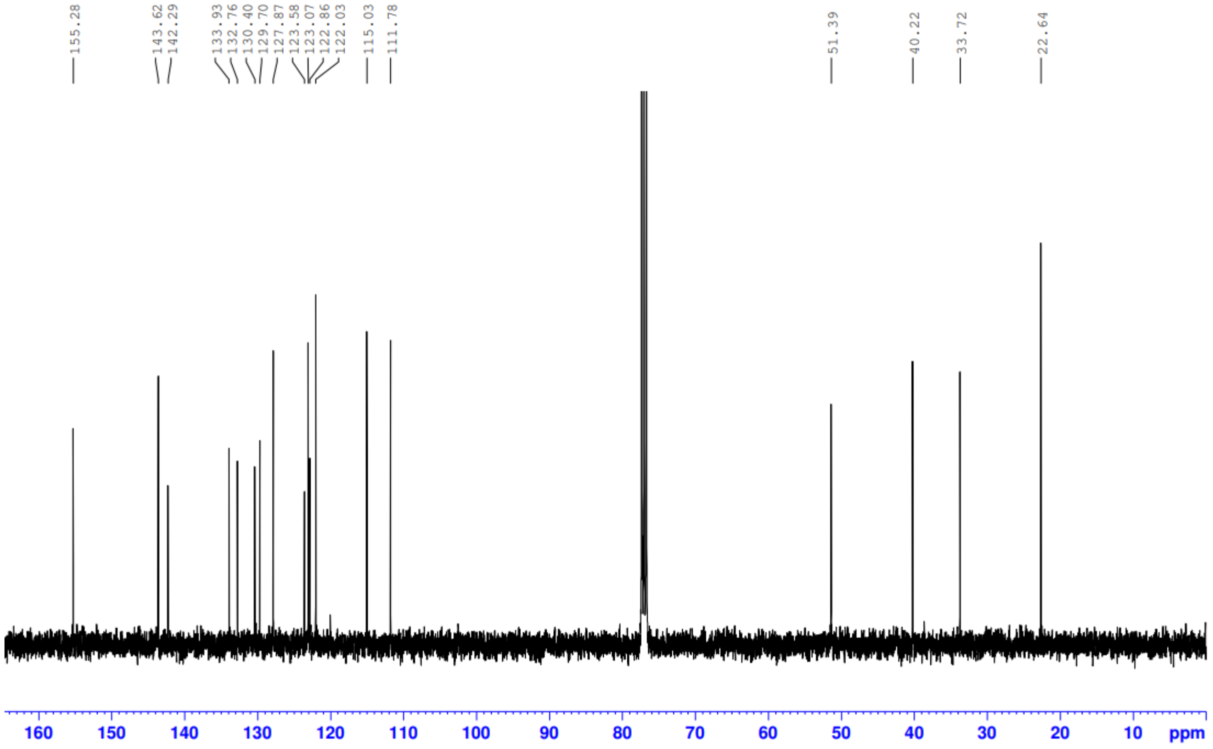

9-Allyl-2,4-dichloro-5-methyl-5,6-dihydrophenanthridin-8-ol (12a).

^{1}H NMR (400 MHz, $CDCl_3$)

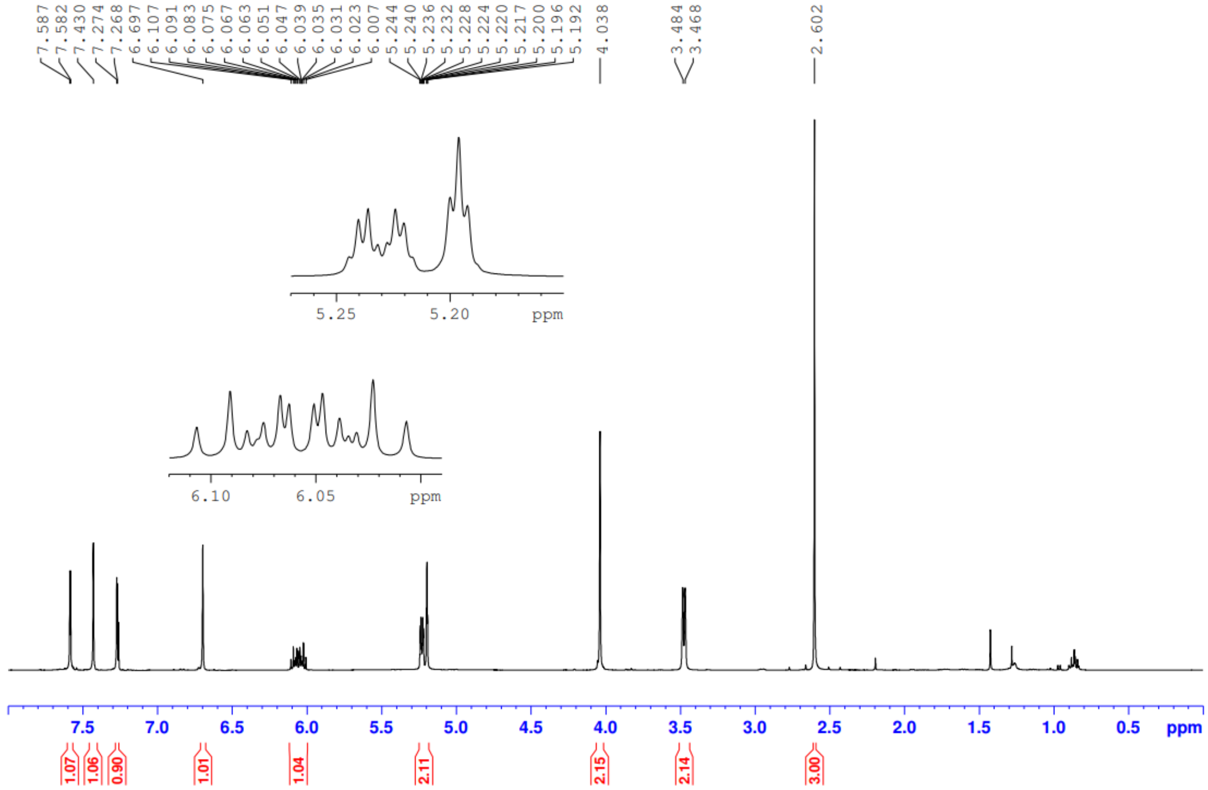

^{13}C NMR (100 MHz, $CDCl_3$)

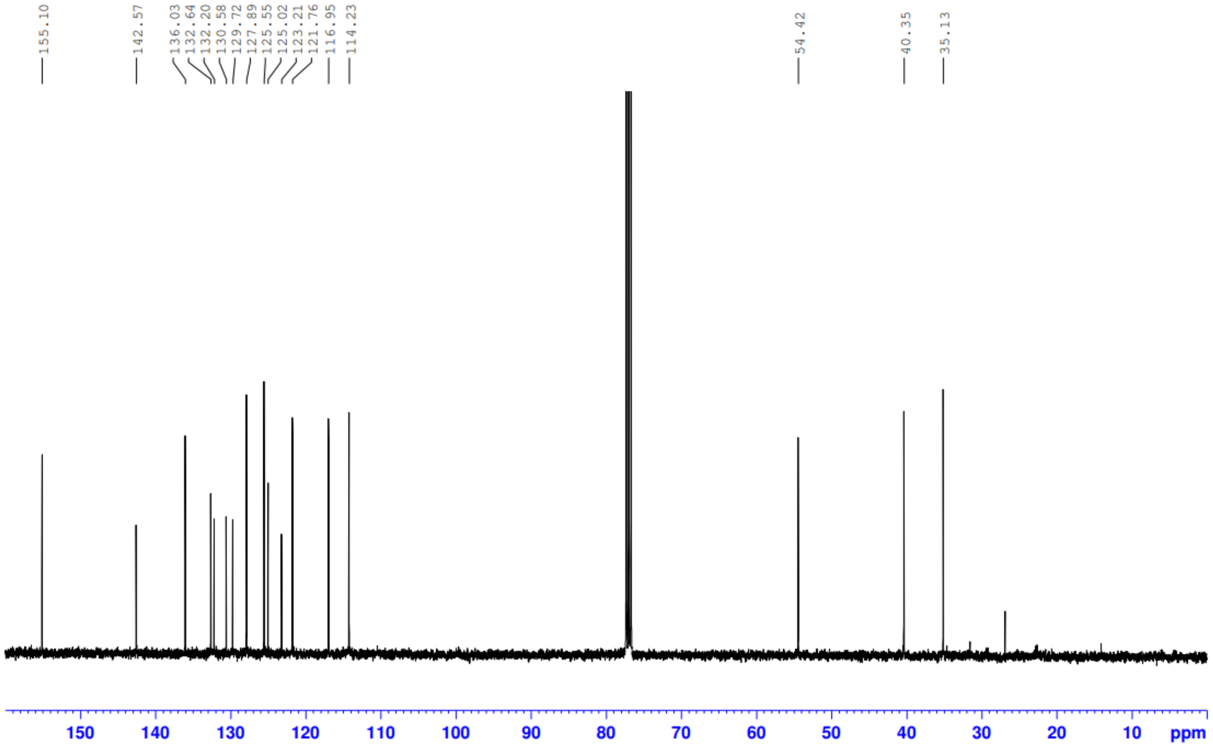

2,4-Dichloro-5-methyl-9-(2-methylallyl)-5,6-dihydrophenanthridin-8-ol (12b).

^{1}H NMR (400 MHz, $CDCl_3$)

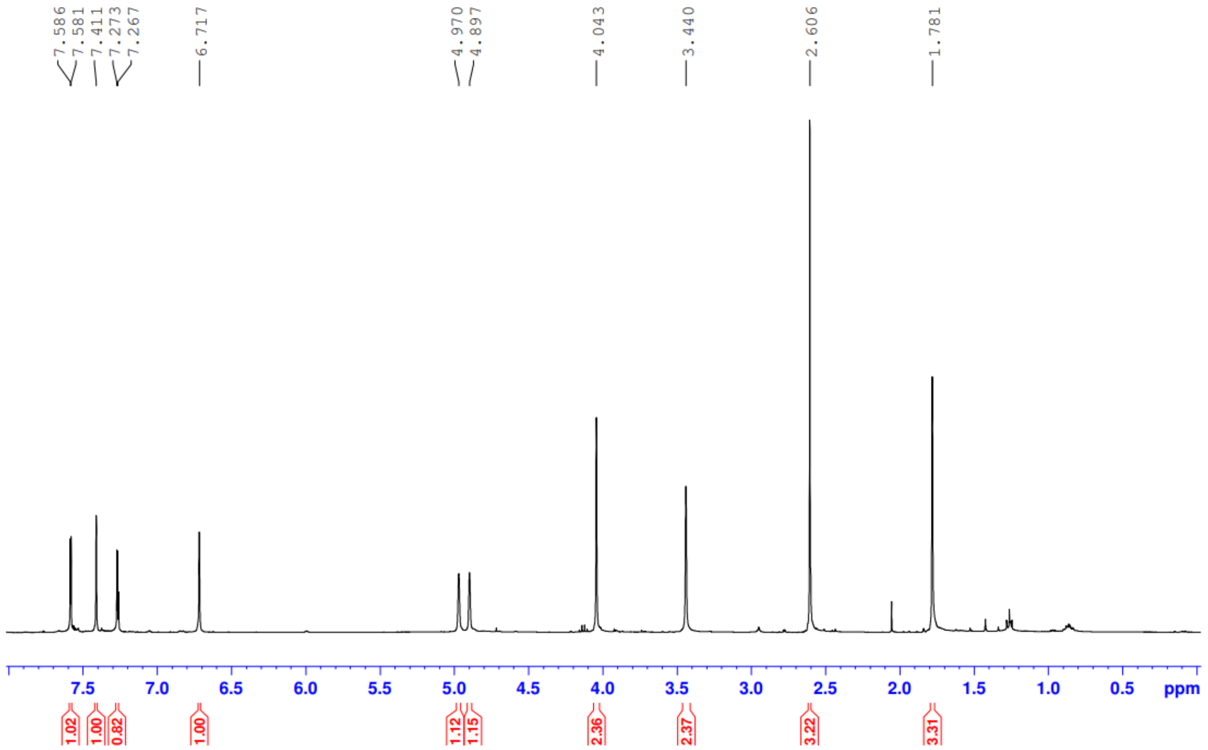

^{13}C NMR (100 MHz, $CDCl_3$)

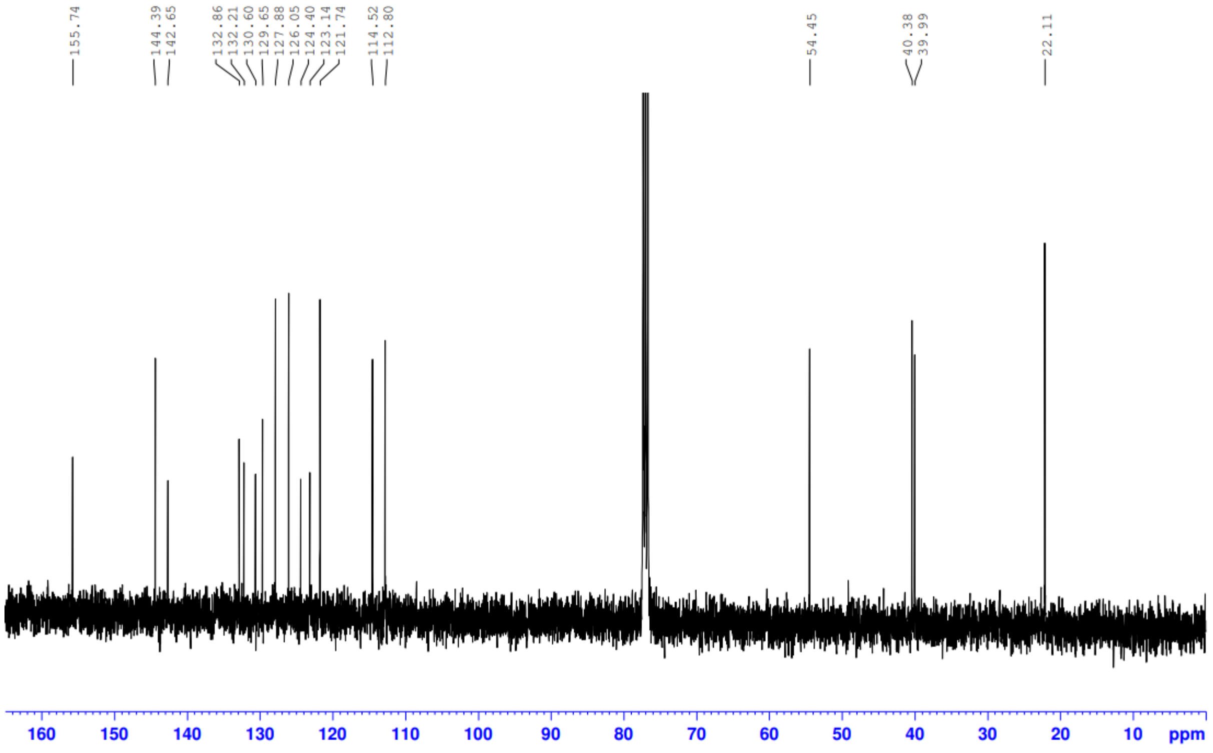

7-Allyl-2,4-dichloro-8-hydroxy-5-methylphenanthridin-6(5*H*)-one (13a).

^{1}H NMR (400 MHz, acetone-d_6)

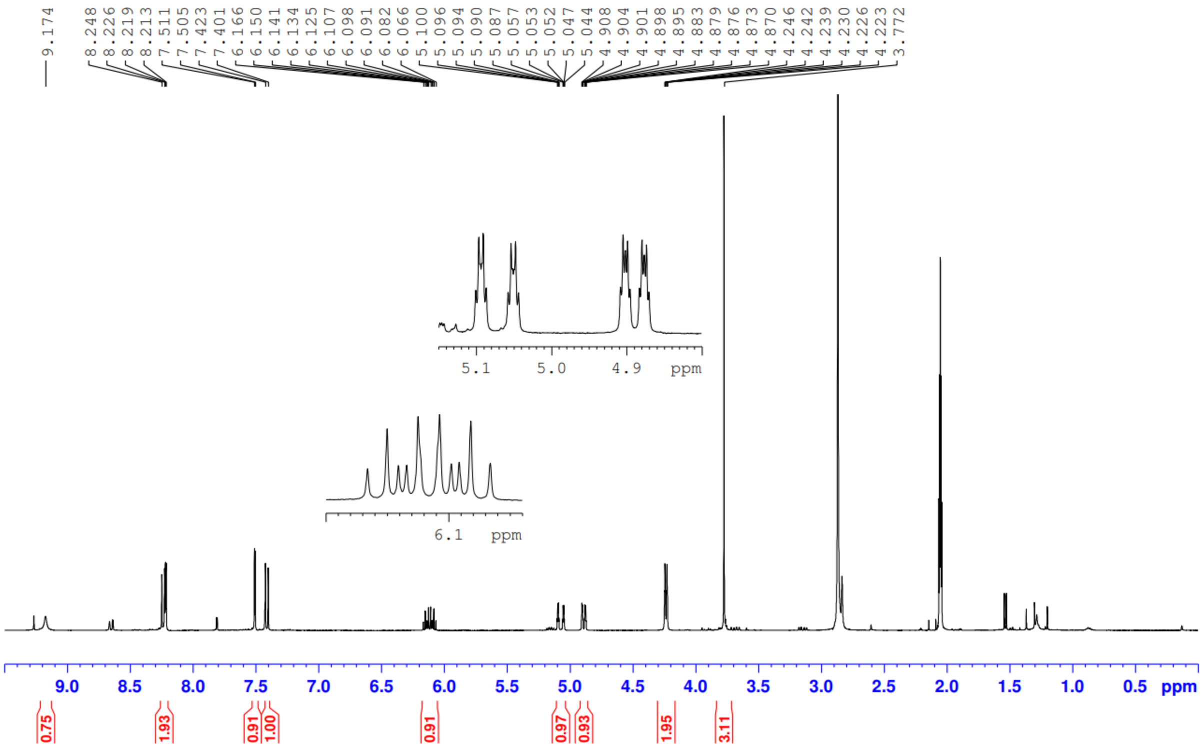

^{13}C NMR (100 MHz, acetone-d_6)

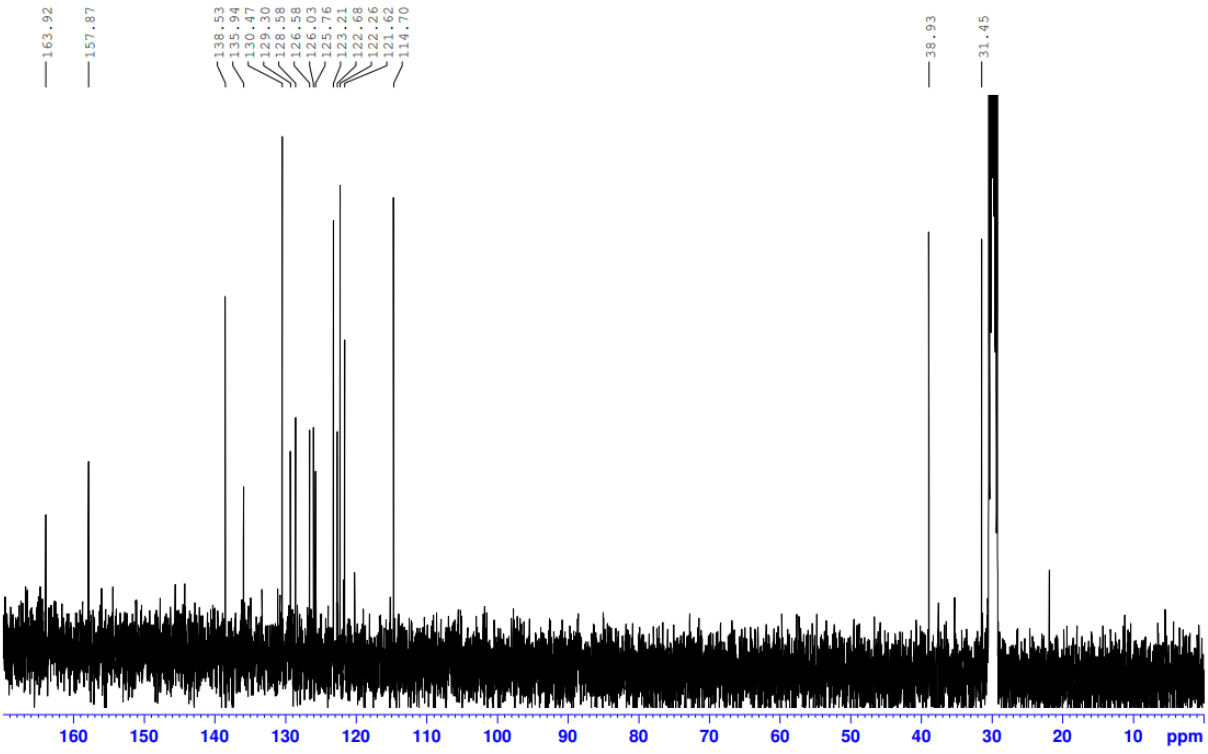

6,8-Dichloro-2,2,5-trimethyl-2,3,4,5-tetrahydrofuro[3,2-*i*]phenanthridine (14b).

^{1}H NMR (400 MHz, $CDCl_3$)

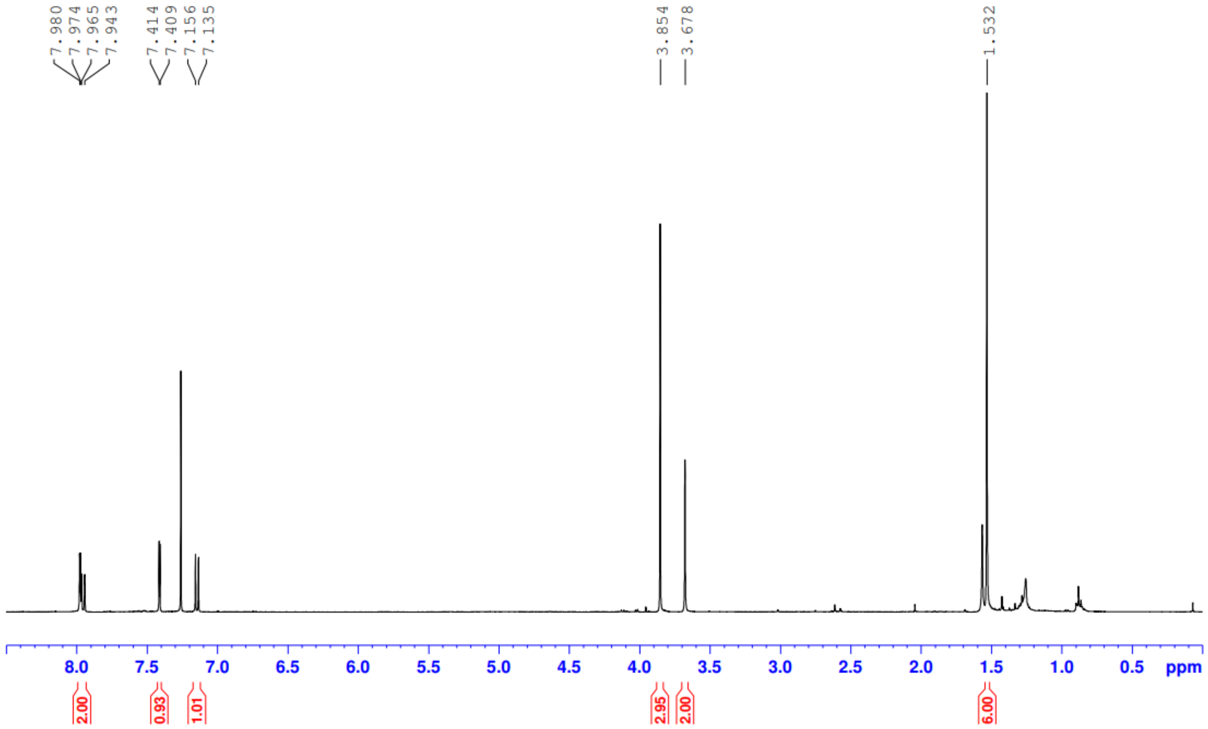

^{13}C NMR (100 MHz, $CDCl_3$)

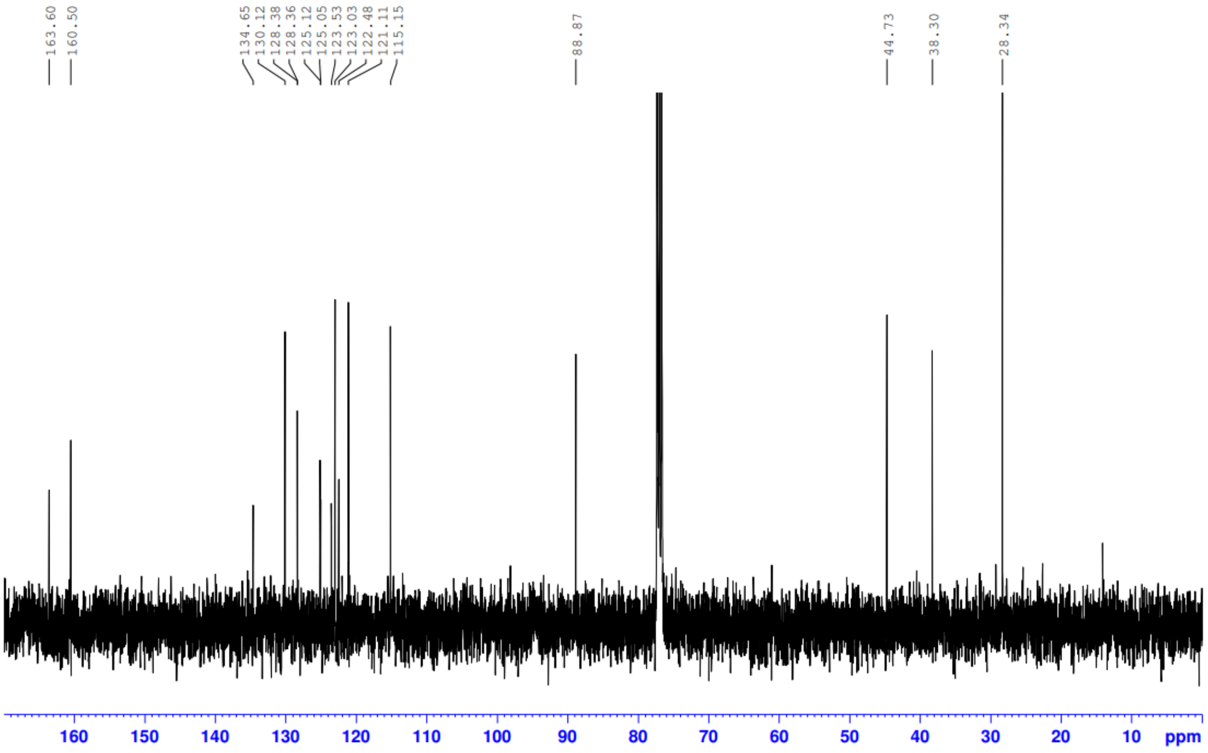

Supplement: Supplementary file 1 — Supporting Information [file OPEN-12-e202300095-s001.pdf]
